# Supplementary material for: PPNet: Identifying Functional Association Networks by Phylogenetic Profiling of Prokaryotic Genomes
Source: Microbiol Spectr. 2023 Jan 5;11(1):e03871-22. doi: 10.1128/spectrum.03871-22 (PMC9927313; doi:10.1128/spectrum.03871-22)
Supplement: Supplemental file 1 — Supplemental material. Download spectrum.03871-22-s0001.pdf, PDF file, 4.7 MB [file spectrum.03871-22-s0001.pdf]

Supplemental Figures and Tables

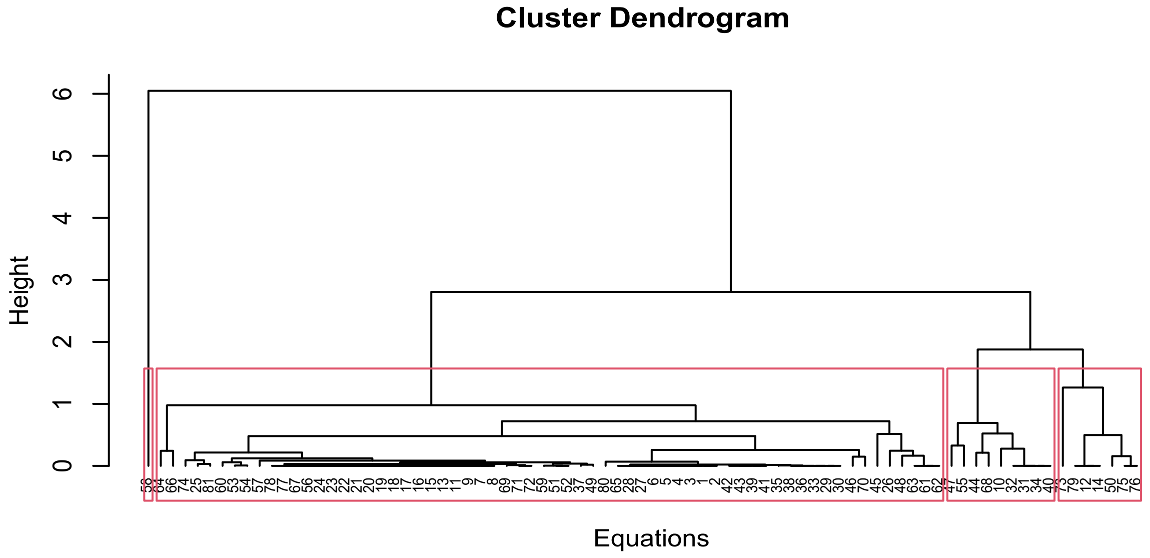

Figure S1 Dendrogram of the 81 binary similarity and distance measures. The 81 equations were divided into four groups by hierarchical clustering based on the minimum distance of the ROC curve to the theoretical optimum point and AUROC (red boxes).

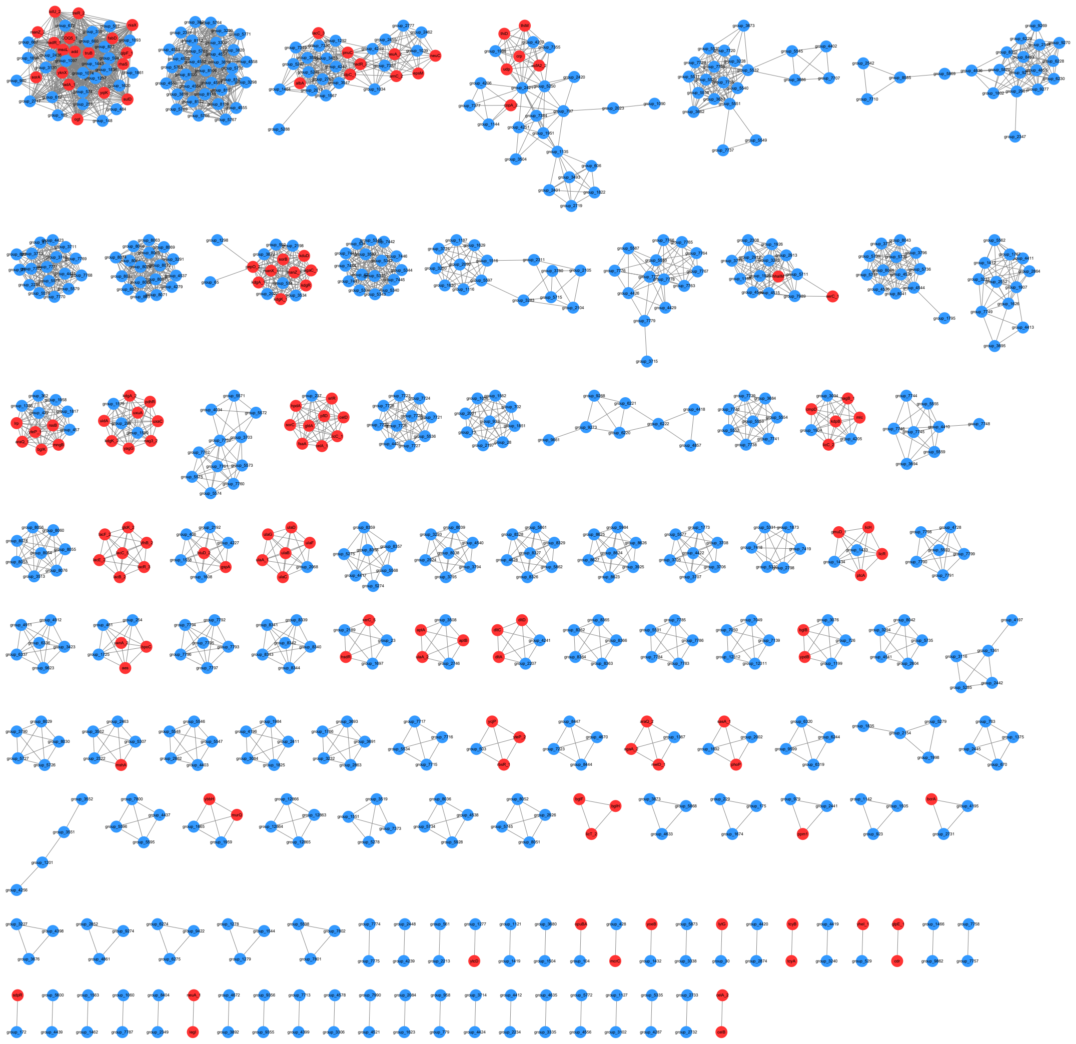

Figure S2 VRDGs association network of *S. suis* inferred by PPNet. The VRDGs association network of *S. suis* connecting 753 genes with 3215 interactions for *S. suis*, which was inferred by PPNet based on OCHIAI-II binary similarity coefficient and visualized by Cytoscape. Each node represents a gene, and the edge between nodes represent the interactions. The red node indicates that the gene has an annotated name.

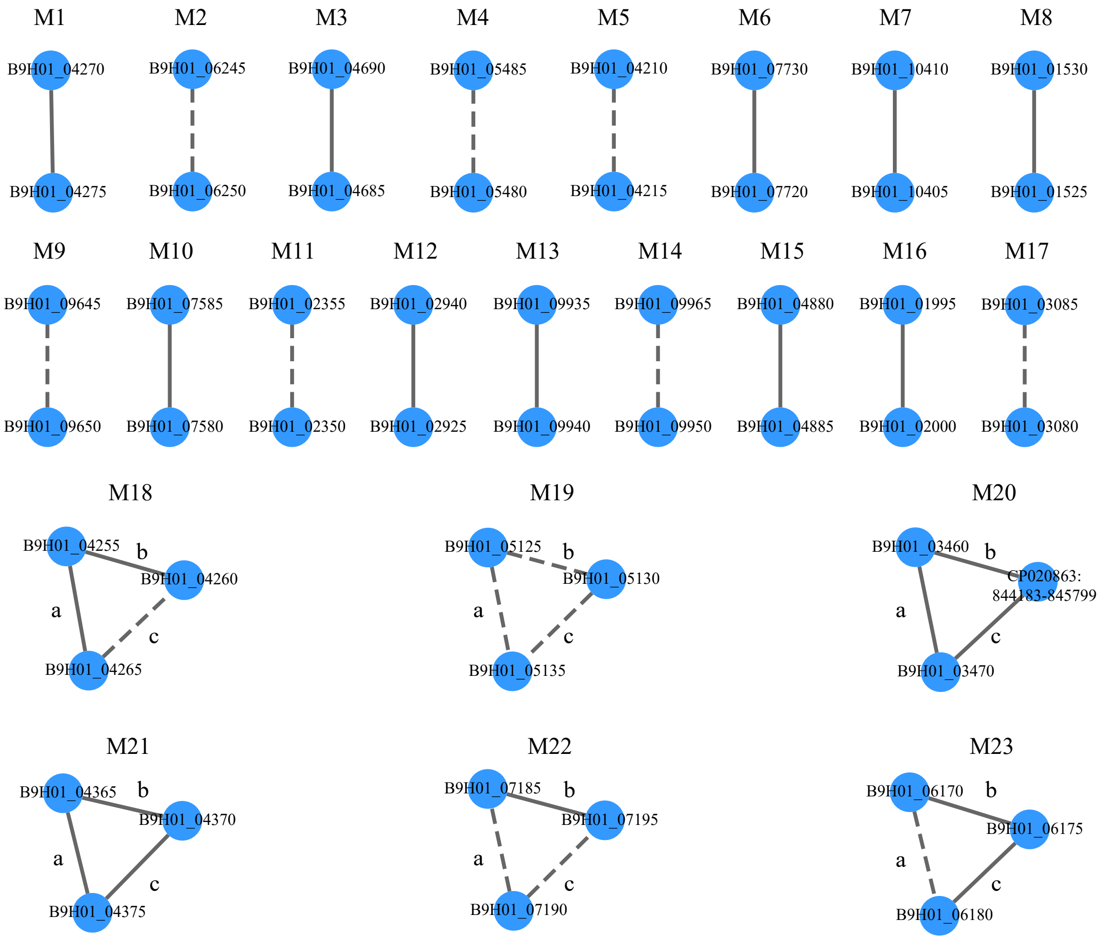

Figure S3 Selected modules to be verified. All two-gene modules and the three-gene interaction modules in the predicted virulence related differential gene interaction network based on high-virulent field strain *S. suis* SC19 strain constructed by OCHIAI-II similarity. The solid line indicates the interaction was verified as positive in the bacterial two-hybrid experiment, and the dashed line indicates that the interaction was negative.

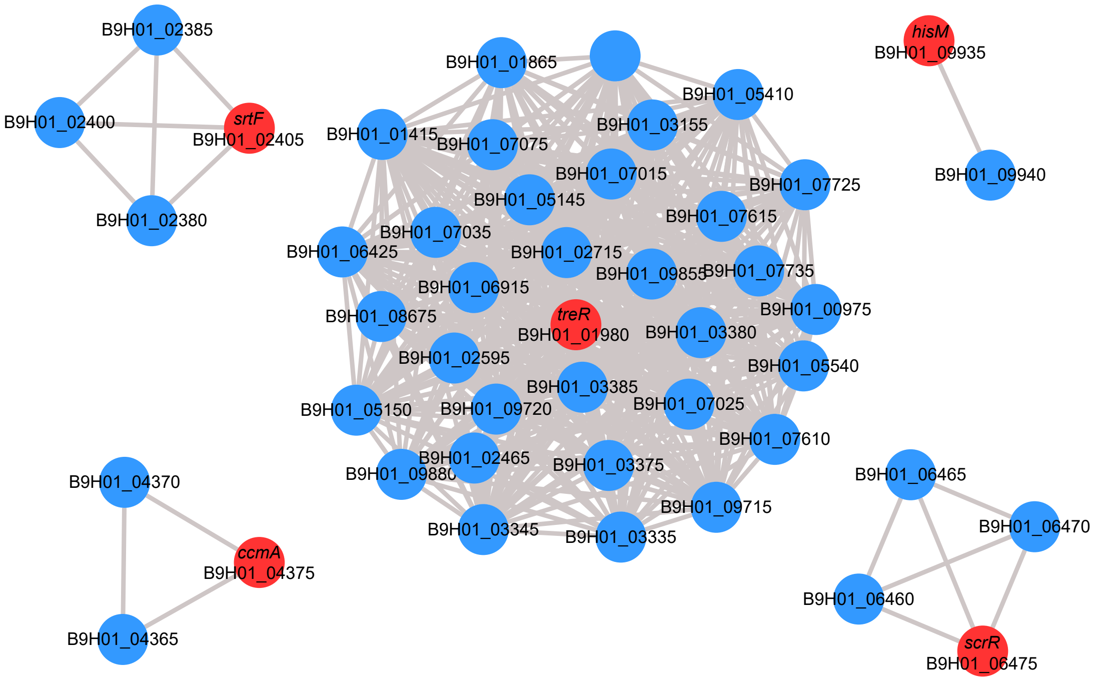

Figure S4 VRDGs summarized before in the network we constructed. Red colored VRDGs in the virulence-related gene association network based on high-virulent *S. suis* SC19 strain constructed by OCHIAI-II similarity summarized as *S. suis* virulence factors previously.

Table S1 The background of 1288 *Streptococcus suis* strains used in this study

Strains	ReName	Assembly	Size (Mb)	GC%	N50	Molecular Serotypes	Country	Level	Release Date	Note
SC218	SS1000	GCA_002759905.1	1.70851	42.1	1143	2&1/2	China	Contig	2017/11/7	Low quality (N50<10000)
CQ1	SS1002	GCA_002760005.1	1.80102	41.9	1152	2&1/2	China	Contig	2017/11/7	Low quality (N50<10000)
SC5	SS1004	GCA_002759475.1	1.79795	41.9	1155	2&1/2	China	Contig	2017/11/7	Low quality (N50<10000)
YS178	SS1005	GCA_002760295.1	1.97264	42.1	1155	NCL10	China	Contig	2017/11/7	Low quality (N50<10000)
SC39	SS1007	GCA_002759555.1	1.79906	41.9	1155	2&1/2	China	Contig	2017/11/7	Low quality (N50<10000)
SC153	SS1009	GCA_002759805.1	1.80125	41.9	1155	2&1/2	China	Contig	2017/11/7	Low quality (N50<10000)
JXS1	SS1010	GCA_002759595.1	1.80203	41.9	1155	2&1/2	China	Contig	2017/11/7	Low quality (N50<10000)
YS145	SS1011	GCA_002760205.1	1.99401	42.1	1152	NCL8	China	Contig	2017/11/7	Low quality (N50<10000)
SC129	SS1012	GCA_002759485.1	1.78107	42	1152	2&1/2	China	Contig	2017/11/7	Low quality (N50<10000)
JX1	SS1013	GCA_002759585.1	1.79969	41.9	1161	2&1/2	China	Contig	2017/11/7	Low quality (N50<10000)
YS159	SS1015	GCA_002759715.1	1.93721	42.1	1158	NCL8	China	Contig	2017/11/7	Low quality (N50<10000)
YS108	SS1016	GCA_002760085.1	2.02669	42.3	1149	NCL9	China	Contig	2017/11/7	Low quality (N50<10000)
GZ2	SS1019	GCA_002759615.1	1.79851	41.9	1149	2&1/2	China	Contig	2017/11/7	Low quality (N50<10000)
GD1	SS1020	GCA_002759995.1	1.80105	41.9	1155	2&1/2	China	Contig	2017/11/7	Low quality (N50<10000)
SC220	SS978	GCA_002759875.1	1.797	41.9	1152	2&1/2	China	Contig	2017/11/7	Low quality (N50<10000)
SC17	SS979	GCA_002759925.1	1.7984	41.9	1152	2&1/2	China	Contig	2017/11/7	Low quality (N50<10000)
SC112	SS980	GCA_002759535.1	1.80068	41.9	1155	2&1/2	China	Contig	2017/11/7	Low quality (N50<10000)

Strains	ReName	Assembly	Size (Mb)	GC%	N50	Molecular Serotypes	Country	Level	Release Date	Note
SC16	SS981	GCA_002759525.1	1.79896	41.9	1155	2&1/2	China	Contig	2017/11/7	Low quality (N50<10000)
SC3	SS982	GCA_002759795.1	1.80012	41.9	1152	2&1/2	China	Contig	2017/11/7	Low quality (N50<10000)
SC22	SS983	GCA_002759975.1	1.79713	41.9	1152	2&1/2	China	Contig	2017/11/7	Low quality (N50<10000)
SC333	SS985	GCA_002759985.1	1.80177	41.9	1155	2&1/2	China	Contig	2017/11/7	Low quality (N50<10000)
YS173	SS989	GCA_002760285.1	2.06088	42.2	1170	NCL8	China	Contig	2017/11/7	Low quality (N50<10000)
SC160	SS994	GCA_002759815.1	1.79817	41.9	1155	2&1/2	China	Contig	2017/11/7	Low quality (N50<10000)
YS172	SS995	GCA_002760265.1	1.91118	42.3	1143	30	China	Contig	2017/11/7	Low quality (N50<10000)
SC248	SS997	GCA_002759915.1	1.80079	41.9	1155	2&1/2	China	Contig	2017/11/7	Low quality (N50<10000)
SC211	SS998	GCA_002759895.1	1.79977	41.9	1152	2&1/2	China	Contig	2017/11/7	Low quality (N50<10000)
SC149	SS999	GCA_002759825.1	1.80034	41.9	1155	2&1/2	China	Contig	2017/11/7	Low quality (N50<10000)
SRD478	SS772	GCA_001293375.1	1.71045	41.8	9865	not hit	USA	Contig	2015/9/17	Low quality (N50<10000)
88-1861	SS270	GCA_000440875.1	2.27225	39.7	35704	22	Canada	Contig	2013/7/25	Non-*S. suis* (ANI<95%)
89-4109-1	SS631	GCA_000440655.1	2.17673	39.9	64745	26	Canada	Contig	2013/7/25	Non-*S. suis* (ANI<95%)
86-5192	SS764	GCA_000440555.1	2.11017	39.9	86346	20	Canada	Contig	2013/7/25	Non-*S. suis* (ANI<95%)
EA1832.92	SS720	GCA_000478745.1	1.98633	40	35588	33	Canada	Scaffold	2013/10/22	Non-*S. suis* (ANI<95%)
YS56	SS139	GCA_000440115.1	2.23011	41.2	50822	NCL1	China	Contig	2013/7/25	Non-*S. suis* (ANI<95%)
YS161	SS1003	GCA_002760245.1	2.29457	41.1	48504	22	China	Contig	2017/11/7	Non-*S. suis* (ANI<95%)
S33	SS1008	GCA_002759445.1	2.025	40.1	63919	33	China	Contig	2017/11/7	Non-*S. suis* (ANI<95%)
2774	SS1063	GCA_002960165.1	3.00966	41	49260	NCL8	China	Contig	2018/2/28	Non-*S. suis* (ANI<95%)

Strains	ReName	Assembly	Size (Mb)	GC%	N50	Molecular Serotypes	Country	Level	Release Date	Note
1463	SS1064	GCA_002960955.1	2.87479	41.2	24601	21	China	Contig	2018/2/28	Non-*S. suis* (ANI<95%)
1776	SS1068	GCA_002962605.1	3.01958	42.7	59358	16	China	Contig	2018/2/28	Non-*S. suis* (ANI<95%)
2843	SS1071	GCA_002960005.1	2.26703	43.3	46219	not hit	China	Contig	2018/2/28	Non-*S. suis* (ANI<95%)
2847	SS1074	GCA_002960055.1	2.57868	41.4	41541	31	China	Contig	2018/2/28	Non-*S. suis* (ANI<95%)
2578	SS1080	GCA_002960245.1	2.74736	41	38924	not hit	China	Contig	2018/2/28	Non-*S. suis* (ANI<95%)
2256	SS1089	GCA_002960425.1	2.09612	42.3	73292	not hit	China	Contig	2018/2/28	Non-*S. suis* (ANI<95%)
2219	SS1090	GCA_002960445.1	2.2025	42.2	102783	12	China	Contig	2018/2/28	Non-*S. suis* (ANI<95%)
2231	SS1092	GCA_002960485.1	2.16322	42.2	61641	not hit	China	Contig	2018/2/28	Non-*S. suis* (ANI<95%)
2741	SS1096	GCA_002960525.1	2.51839	42.9	91744	not hit	China	Contig	2018/2/28	Non-*S. suis* (ANI<95%)
2674	SS1097	GCA_002960535.1	2.49517	39.7	37173	not hit	China	Contig	2018/2/28	Non-*S. suis* (ANI<95%)
2187	SS1101	GCA_002960625.1	2.13843	42.1	88171	not hit	China	Contig	2018/2/28	Non-*S. suis* (ANI<95%)
1953	SS1110	GCA_002960845.1	2.72095	42.7	78941	not hit	China	Contig	2018/2/28	Non-*S. suis* (ANI<95%)
1225	SS1132	GCA_002961305.1	2.52739	41.1	73153	16	China	Contig	2018/2/28	Non-*S. suis* (ANI<95%)
666	SS1187	GCA_002962445.1	2.10723	43.7	60008	not hit	China	Contig	2018/2/28	Non-*S. suis* (ANI<95%)
614	SS1262	GCA_002964045.1	2.51023	41.3	29877	not hit	China	Contig	2018/2/28	Non-*S. suis* (ANI<95%)
15	SS1282	GCA_002964575.1	2.2671	41.8	116218	not hit	China	Contig	2018/2/28	Non-*S. suis* (ANI<95%)
WUSS424	SS1025	GCA_004922885.1	2.21703	43.2	485228	not hit	China	Scaffold	2019/4/29	Non-*S. suis* (ANI<95%)
PP422	SS1040	GCA_003934335.1	2.07566	43.7	101925	not hit	Switzerland	Contig	2018/12/10	Non-*S. suis* (ANI<95%)
UBA1166	SS382	GCA_002311075.1	1.81846	39.4	105566	not hit	USA	Scaffold	2017/9/19	Non-*S. suis* (ANI<95%)

Strains	ReName	Assembly	Size (Mb)	GC%	N50	Molecular Serotypes	Country	Level	Release Date	Note
UBA1638	SS221	GCA_002321175.1	1.71799	39.3	18621	not hit	USA	Scaffold	2017/9/21	Non-*S. suis* (ANI<95%)
UBA2099	SS318	GCA_002331205.1	1.7196	39.7	17206	not hit	USA	Scaffold	2017/9/21	Non-*S. suis* (ANI<95%)
UBA5655	SS783	GCA_002420965.1	1.77783	39.3	72039	not hit	USA	Scaffold	2017/10/3	Non-*S. suis* (ANI<95%)
UBA5922	SS421	GCA_002431745.1	1.92262	39.5	65835	not hit	USA	Scaffold	2017/10/4	Non-*S. suis* (ANI<95%)
UBA7628	SS069	GCA_002484025.1	1.76397	39.5	30228	not hit	USA	Scaffold	2017/10/6	Non-*S. suis* (ANI<95%)
UBA7340	SS250	GCA_002471125.1	1.85599	39.5	59582	not hit	USA	Scaffold	2017/10/6	Non-*S. suis* (ANI<95%)
UBA7036	SS895	GCA_002494085.1	1.23313	39.2	10773	not hit	USA	Scaffold	2017/10/10	Non-*S. suis* (ANI<95%)
161_00P5	SS416	GCA_000440375.1	2.26721	41.4	48080	NCL1	Argentina	Contig	2013/7/25	
89/1591	SS255	GCA_000167375.1	2.14325	41.1	54360	2&1/2	Canada	Contig	2004/7/23	
93A	SS088	GCA_000440755.1	2.3269	41.1	23977	17	Canada	Contig	2013/7/25	
89-3576-3	SS108	GCA_000440635.1	2.12613	41.3	66619	25	Canada	Contig	2013/7/25	
89-590	SS207	GCA_000440915.1	2.16164	41.2	58552	28	Canada	Contig	2013/7/25	
92-1191	SS211	GCA_000440695.1	2.35428	41.3	26198	29	Canada	Contig	2013/7/25	
88-5299A	SS278	GCA_000440895.1	2.33337	41.3	45671	24	Canada	Contig	2013/7/25	
NT77	SS329	GCA_000440775.1	2.31631	41	27567	18	Canada	Contig	2013/7/25	
14A	SS392	GCA_000440355.1	2.34792	41.4	37081	21	Canada	Contig	2013/7/25	
42A	SS598	GCA_000440855.1	2.29008	41.2	21076	19	Canada	Contig	2013/7/25	
89-1591	SS618	GCA_000440595.1	2.14688	41.1	32487	2&1/2	Canada	Contig	2013/7/25	
89-2479	SS684	GCA_000440615.1	2.16808	41	141868	23	Canada	Contig	2013/7/25	

Strains	ReName	Assembly	Size (Mb)	GC%	N50	Molecular Serotypes	Country	Level	Release Date	Note
92-4172	SS738	GCA_000440735.1	2.3039	41.3	50993	31	Canada	Contig	2013/7/25	
89-5259	SS835	GCA_000440675.1	2.45846	41.3	39482	27	Canada	Contig	2013/7/25	
92-1400	SS881	GCA_000440715.1	2.30598	40.8	60330	30	Canada	Contig	2013/7/25	
NSUI002	SS964	GCA_001272635.1	2.25535	41.1	2255345	2&1/2	Canada	Complete Genome	2015/8/21	
NSUI060	SS913	GCA_001572685.1	2.30221	41.09	2285232	2&1/2	Canada	Complete Genome	2016/2/25	
90-1330	SS267	GCA_001610935.1	2.14615	41.1	2146151	2&1/2	Canada	Complete Genome	2016/4/5	
98HAH33	SS125	GCA_000014325.1	2.0957	41.1	2095698	2&1/2	China	Complete Genome	2007/4/24	
05ZYH33	SS444	GCA_000014305.1	2.09631	41.1	2096309	2&1/2	China	Complete Genome	2007/4/24	
SC84	SS571	GCA_000026725.1	2.0959	41.1	2095898	2&1/2	China	Complete Genome	2009/7/7	
GZ1	SS049	GCA_000018185.1	2.03803	41.4	2038034	2&1/2	China	Complete Genome	2010/3/30	
JS14	SS548	GCA_000186405.1	2.13743	41.2	2137435	1&14	China	Complete Genome	2011/1/20	
ST3	SS960	GCA_000204625.1	2.02881	41.3	2028815	3	China	Complete Genome	2011/4/19	
R61	SS754	GCA_000231325.2	2.39091	41.2	97080	12	China	Contig	2011/10/21	
D12	SS465	GCA_000231905.1	2.18306	41.3	2183059	9	China	Complete Genome	2011/10/25	
D9	SS541	GCA_000231885.1	2.17766	41	2177656	7	China	Complete Genome	2011/10/25	
ST1	SS544	GCA_000231925.1	2.03432	41.4	2034321	1&14	China	Complete Genome	2011/10/25	
SS12	SS590	GCA_000231865.1	2.09687	41.2	2096866	2&1/2	China	Complete Genome	2011/10/25	
A7	SS375	GCA_000233575.1	2.03841	41.2	2038409	2&1/2	China	Complete Genome	2011/11/1	
SC070731	SS064	GCA_000344765.1	2.13857	41.2	2138568	2&1/2	China	Complete Genome	2013/3/6	

Strains	ReName	Assembly	Size (Mb)	GC%	N50	Molecular Serotypes	Country	Level	Release Date	Note
TL13	SS692	GCA_000390245.1	2.03815	41.3	2038146	16	China	Complete Genome	2013/5/13	
YS77	SS060	GCA_000441475.1	2.26225	41.4	38176	29	China	Contig	2013/7/25	
YS12	SS076	GCA_000440975.1	2.07854	41.1	70322	7	China	Contig	2013/7/25	
YS66	SS103	GCA_000441435.1	2.05962	41.2	90465	7	China	Contig	2013/7/25	
YS39	SS122	GCA_000441235.1	2.23988	41	74051	12	China	Contig	2013/7/25	
YS54	SS134	GCA_000440095.1	2.31479	41.2	39866	29	China	Contig	2013/7/25	
YS53	SS151	GCA_000441375.1	2.20743	41.1	111182	11	China	Contig	2013/7/25	
YS4	SS176	GCA_000441335.1	2.1096	41.1	65500	2&1/2	China	Contig	2013/7/25	
YS72	SS219	GCA_000440175.1	2.35965	41.4	57103	NCL2	China	Contig	2013/7/25	
YS27	SS280	GCA_000441155.1	2.42529	41.2	38696	NCL3	China	Contig	2013/7/25	
YS3	SS296	GCA_000441255.1	2.16903	41	85411	11	China	Contig	2013/7/25	
RC1	SS299	GCA_000440815.1	2.19643	41	66898	8	China	Contig	2013/7/25	
07SC3	SS393	GCA_000440215.1	2.06109	41	130857	2&1/2	China	Contig	2013/7/25	
YS7	SS402	GCA_000441495.1	2.35108	41.3	45259	NCL1	China	Contig	2013/7/25	
YS50	SS429	GCA_000441355.1	2.41721	41.3	36041	29	China	Contig	2013/7/25	
YS23	SS437	GCA_000441115.1	2.34587	41.4	52940	NCL2	China	Contig	2013/7/25	
YS14	SS449	GCA_000440995.1	2.17152	41	78424	8	China	Contig	2013/7/25	
YS21	SS494	GCA_000441095.1	2.46878	41.3	29489	NCL1	China	Contig	2013/7/25	
YS16	SS506	GCA_000441015.1	2.32931	41	82793	31	China	Contig	2013/7/25	

Strains	ReName	Assembly	Size (Mb)	GC%	N50	Molecular Serotypes	Country	Level	Release Date	Note
YS64	SS511	GCA_000441415.1	2.06667	41.1	55972	11	China	Contig	2013/7/25	
YS34	SS517	GCA_000441195.1	2.37469	41	61738	NCL4	China	Contig	2013/7/25	
YS10	SS573	GCA_000440955.1	2.22046	41	102188	12	China	Contig	2013/7/25	
YS43	SS597	GCA_000441275.1	2.0518	41.2	56301	NCL6	China	Contig	2013/7/25	
YS31	SS604	GCA_000441175.1	2.22714	41	81321	12	China	Contig	2013/7/25	
YS74	SS662	GCA_000440195.1	2.46635	41.2	37496	NCL3	China	Contig	2013/7/25	
YS6	SS663	GCA_000440155.1	2.03884	41.1	91202	2&1/2	China	Contig	2013/7/25	
YS17	SS669	GCA_000441035.1	2.33768	41	65182	31	China	Contig	2013/7/25	
YS24	SS719	GCA_000441135.1	2.10712	41.2	64280	10	China	Contig	2013/7/25	
YS57	SS766	GCA_000440135.1	2.51791	41.2	35828	NCL7	China	Contig	2013/7/25	
YS49	SS798	GCA_000440075.1	2.40826	41.3	32243	NCL1	China	Contig	2013/7/25	
YS59	SS804	GCA_000441395.1	2.20079	41	100499	12	China	Contig	2013/7/25	
YS35	SS853	GCA_000441215.1	2.3201	40.9	76376	NCL5	China	Contig	2013/7/25	
YS19	SS883	GCA_000441055.1	2.1088	41.1	62423	2&1/2	China	Contig	2013/7/25	
YS46	SS894	GCA_000441315.1	2.42666	41.2	29549	NCL7	China	Contig	2013/7/25	
YS67	SS899	GCA_000441455.1	2.18055	41.1	84895	11	China	Contig	2013/7/25	
R735	SS910	GCA_000440795.1	1.94957	41.4	81905	2&1/2	China	Contig	2013/7/25	
YS44	SS940	GCA_000441295.1	2.20716	41.1	122627	11	China	Contig	2013/7/25	
YS1	SS950	GCA_000441075.1	2.03025	41.3	107606	2&1/2	China	Contig	2013/7/25	

Strains	ReName	Assembly	Size (Mb)	GC%	N50	Molecular Serotypes	Country	Level	Release Date	Note
YB51	SS445	GCA_000471985.1	2.04366	41.3	2043655	3	China	Complete Genome	2013/9/27	
T15	SS840	GCA_000494895.1	2.24023	41	2240234	2&1/2	China	Complete Genome	2013/11/1	
05HAS68	SS955	GCA_000168355.3	2.17607	41.2	2176073	2&1/2	China	Complete Genome	2014/1/9	
ZY05719	SS936	GCA_000993745.1	2.0949	41.1	2094898	2&1/2	China	Complete Genome	2015/5/7	
DN13	SS092	GCA_001640765.1	2.14218	41.3	2142184	9	China	Complete Genome	2016/5/9	
GZ0565	SS387	GCA_001721625.1	2.13188	41.3	2131882	9	China	Complete Genome	2016/9/8	
SC19	SS713	GCA_001866735.1	2.07331	41	223803	2&1/2	China	Contig	2016/11/4	
SS2-1	SS876	GCA_001936415.1	2.09403	41.1	2094030	2&1/2	China	Complete Genome	2017/1/5	
YY060816	SS356	GCA_001945645.1	2.08922	41.2	75133	2&1/2	China	Contig	2017/1/11	
SC19	SS706	GCA_002111405.1	2.09597	41.1	2095966	2&1/2	China	Complete Genome	2017/4/26	
LSM102	SS574	GCA_002189695.1	2.06793	41.2	2067932	2&1/2	China	Complete Genome	2017/6/12	
CS100322	SS733	GCA_002688665.1	2.13765	41.2	2137649	2&1/2	China	Complete Genome	2017/10/24	
HA0609	SS988	GCA_002736265.1	2.24735	41.1	2247350	2&1/2	China	Complete Genome	2017/10/30	
YS146	SS1001	GCA_002760225.1	2.42597	41.2	54569	NCL8	China	Contig	2017/11/7	
YS111	SS1006	GCA_002760125.1	2.59131	41.2	33923	NCL8	China	Contig	2017/11/7	
YS104	SS1014	GCA_002759725.1	2.44505	41.3	24179	NCL8	China	Contig	2017/11/7	
YS107	SS1017	GCA_002760055.1	2.34945	41.4	21480	NCL8	China	Contig	2017/11/7	
YS131	SS1018	GCA_002760195.1	2.57841	41.2	35326	NCL8	China	Contig	2017/11/7	
YS93	SS976	GCA_002759695.1	2.29793	41.2	29967	NCL3	China	Contig	2017/11/7	

Strains	ReName	Assembly	Size (Mb)	GC%	N50	Molecular Serotypes	Country	Level	Release Date	Note
YS85	SS977	GCA_002760075.1	2.45108	41.3	30035	NCL8	China	Contig	2017/11/7	
YS106	SS984	GCA_002759735.1	2.37008	41.3	24251	5	China	Contig	2017/11/7	
YS122	SS986	GCA_002759675.1	2.46737	41.4	37721	NCL8	China	Contig	2017/11/7	
YS98	SS987	GCA_002759775.1	2.3118	41.4	44071	NCL2	China	Contig	2017/11/7	
YS117	SS990	GCA_002760135.1	2.38788	41.3	35959	NCL8	China	Contig	2017/11/7	
YS87	SS991	GCA_002759625.1	2.44279	41.2	21615	NCL8	China	Contig	2017/11/7	
YS123	SS992	GCA_002760165.1	2.57468	41.2	33723	NCL8	China	Contig	2017/11/7	
YS110	SS993	GCA_002760105.1	2.6157	41.2	33587	NCL8	China	Contig	2017/11/7	
YS95	SS996	GCA_002760305.1	2.21678	41.3	24928	NCL2	China	Contig	2017/11/7	
CZ130302	SS1288	GCA_002803825.1	2.53889	41.2	2538894	Chz	China	Complete Genome	2017/12/4	
AH681	SS1287	GCA_002812525.1	2.40401	41.5	2404013	Chz	China	Complete Genome	2017/12/6	
HN136	SS1286	GCA_002831545.1	2.71924	41.2	2719245	Chz	China	Complete Genome	2017/12/11	
NJAU-TF-202	SS1285	GCA_002896475.1	2.383	41.2	125053	2&1/2	China	Contig	2018/1/24	
850	SS1062	GCA_002963285.1	2.76128	40.8	61982	12	China	Contig	2018/2/28	
1102	SS1065	GCA_002961545.1	2.77802	41.2	44076	24	China	Contig	2018/2/28	
809	SS1066	GCA_002962285.1	2.86872	40.8	65146	15	China	Contig	2018/2/28	
1612	SS1067	GCA_002962585.1	2.79797	40.9	69907	28	China	Contig	2018/2/28	
1371	SS1069	GCA_002962705.1	2.81528	40.9	78765	4	China	Contig	2018/2/28	
1248	SS1070	GCA_002962895.1	2.79373	41	43075	12	China	Contig	2018/2/28	

Strains	ReName	Assembly	Size (Mb)	GC%	N50	Molecular Serotypes	Country	Level	Release Date	Note
3096	SS1072	GCA_002960015.1	2.34513	41.3	57069	NCL7	China	Contig	2018/2/28	
2873	SS1073	GCA_002960045.1	2.3295	41.1	82568	15	China	Contig	2018/2/28	
2829	SS1075	GCA_002960085.1	2.50893	41.3	35847	NCL8	China	Contig	2018/2/28	
2838	SS1076	GCA_002960105.1	2.62564	41.4	37706	NCL8	China	Contig	2018/2/28	
2828	SS1077	GCA_002960125.1	2.4992	41.3	35841	NCL8	China	Contig	2018/2/28	
2797	SS1078	GCA_002960145.1	2.47593	41.3	36950	NCL8	China	Contig	2018/2/28	
2737	SS1079	GCA_002960185.1	2.46574	41.4	34936	NCL8	China	Contig	2018/2/28	
2464	SS1081	GCA_002960265.1	2.22835	41.1	174519	5	China	Contig	2018/2/28	
2481	SS1082	GCA_002960275.1	2.63015	41	83871	31	China	Contig	2018/2/28	
2355	SS1083	GCA_002960305.1	2.21755	41	92285	5	China	Contig	2018/2/28	
2283	SS1084	GCA_002960325.1	2.11937	41.1	88976	2&1/2	China	Contig	2018/2/28	
2440	SS1085	GCA_002960345.1	2.27229	41.1	55587	16	China	Contig	2018/2/28	
2261	SS1086	GCA_002960355.1	2.16939	40.9	59899	15	China	Contig	2018/2/28	
2254	SS1087	GCA_002960385.1	2.2832	41.5	31843	30	China	Contig	2018/2/28	
2233	SS1088	GCA_002960395.1	2.41373	41.2	44692	NCL2	China	Contig	2018/2/28	
2215	SS1091	GCA_002960455.1	2.28298	41.2	112525	16	China	Contig	2018/2/28	
2214	SS1093	GCA_002960495.1	2.20052	41.2	118898	31	China	Contig	2018/2/28	
2089	SS1094	GCA_002960745.1	2.20621	40.9	64898	15	China	Contig	2018/2/28	
2764	SS1095	GCA_002960195.1	2.40986	41	71514	NCL12	China	Contig	2018/2/28	

Strains	ReName	Assembly	Size (Mb)	GC%	N50	Molecular Serotypes	Country	Level	Release Date	Note
2795	SS1098	GCA_002960555.1	2.48282	41.3	36950	NCL8	China	Contig	2018/2/28	
2761	SS1099	GCA_002960565.1	2.59622	41.3	47581	not hit	China	Contig	2018/2/28	
3-Jan	SS1100	GCA_002960605.1	2.60976	41.3	48301	NCL3	China	Contig	2018/2/28	
2169	SS1102	GCA_002960635.1	2.46376	41.2	53392	12	China	Contig	2018/2/28	
2101	SS1103	GCA_002960665.1	2.36903	41.5	39783	21	China	Contig	2018/2/28	
2100	SS1104	GCA_002960685.1	2.45381	41.3	25328	29	China	Contig	2018/2/28	
2052	SS1105	GCA_002960695.1	2.1256	41.2	95640	30	China	Contig	2018/2/28	
Jan-49	SS1106	GCA_002960725.1	2.20921	41	81350	16	China	Contig	2018/2/28	
1980	SS1107	GCA_002960775.1	2.60462	41	70481	28	China	Contig	2018/2/28	
1961	SS1108	GCA_002960805.1	2.28323	41	88393	31	China	Contig	2018/2/28	
1782	SS1109	GCA_002960825.1	2.19776	41.1	66740	16	China	Contig	2018/2/28	
1721	SS1111	GCA_002960865.1	2.19577	41.2	114918	31	China	Contig	2018/2/28	
1781	SS1112	GCA_002960885.1	2.19361	41.1	62466	16	China	Contig	2018/2/28	
1722	SS1113	GCA_002960905.1	2.2084	41.2	122806	31	China	Contig	2018/2/28	
1700	SS1114	GCA_002960925.1	2.40474	41	51558	31	China	Contig	2018/2/28	
1496	SS1115	GCA_002960945.1	2.40058	40.8	47034	21	China	Contig	2018/2/28	
1416	SS1116	GCA_002960965.1	2.39792	41	160414	not hit	China	Contig	2018/2/28	
1380	SS1117	GCA_002961005.1	2.10855	41.1	143157	12	China	Contig	2018/2/28	
1383	SS1118	GCA_002961025.1	2.24205	41.1	136195	12	China	Contig	2018/2/28	

Strains	ReName	Assembly	Size (Mb)	GC%	N50	Molecular Serotypes	Country	Level	Release Date	Note
1369	SS1119	GCA_002961045.1	2.61983	40.8	71803	4	China	Contig	2018/2/28	
1367	SS1120	GCA_002961055.1	2.53279	41.1	64922	4	China	Contig	2018/2/28	
1358	SS1121	GCA_002961075.1	2.22343	41	92218	NCL4	China	Contig	2018/2/28	
1356	SS1122	GCA_002961105.1	2.51314	40.9	55478	NCL4	China	Contig	2018/2/28	
1318	SS1123	GCA_002961125.1	2.23346	41.1	60739	16	China	Contig	2018/2/28	
1342	SS1124	GCA_002961145.1	2.33188	40.9	73097	11	China	Contig	2018/2/28	
1276	SS1125	GCA_002961165.1	2.33414	40.9	52474	16	China	Contig	2018/2/28	
1314	SS1126	GCA_002961175.1	2.23039	41	64800	16	China	Contig	2018/2/28	
1279	SS1127	GCA_002961205.1	2.55792	41.1	46215	12	China	Contig	2018/2/28	
1275	SS1128	GCA_002961225.1	2.67303	41	50120	15	China	Contig	2018/2/28	
1267	SS1129	GCA_002961235.1	2.19685	40.9	88234	15	China	Contig	2018/2/28	
1251	SS1130	GCA_002961265.1	2.23179	41.1	65758	16	China	Contig	2018/2/28	
1247	SS1131	GCA_002961275.1	2.30272	41	68807	16	China	Contig	2018/2/28	
1219	SS1133	GCA_002961315.1	2.43474	41.4	30774	NCL8	China	Contig	2018/2/28	
1215	SS1134	GCA_002961325.1	2.28661	41.1	48468	16	China	Contig	2018/2/28	
1214	SS1135	GCA_002961355.1	2.18728	41.2	47738	16	China	Contig	2018/2/28	
1200	SS1136	GCA_002961385.1	2.19562	41.1	47792	16	China	Contig	2018/2/28	
1188	SS1137	GCA_002961395.1	2.34611	41.3	50155	NCL7	China	Contig	2018/2/28	
1171	SS1138	GCA_002961425.1	2.36728	41.3	61259	NCL8	China	Contig	2018/2/28	

Strains	ReName	Assembly	Size (Mb)	GC%	N50	Molecular Serotypes	Country	Level	Release Date	Note
1170	SS1139	GCA_002961445.1	2.36116	41.3	61147	NCL8	China	Contig	2018/2/28	
1164	SS1140	GCA_002961465.1	2.64722	41.2	41043	NCL8	China	Contig	2018/2/28	
1141-1	SS1141	GCA_002961485.1	2.42744	41.5	47565	NCL8	China	Contig	2018/2/28	
1140	SS1142	GCA_002961505.1	2.18432	40.9	69168	NCL8	China	Contig	2018/2/28	
1123	SS1143	GCA_002961525.1	2.64311	41.1	46873	12	China	Contig	2018/2/28	
1113	SS1144	GCA_002961555.1	2.57604	41	112254	NCL5	China	Contig	2018/2/28	
1101	SS1145	GCA_002961565.1	2.4679	40.9	53158	not hit	China	Contig	2018/2/28	
1076	SS1146	GCA_002961605.1	2.25755	41	147561	NCL5	China	Contig	2018/2/28	
1070	SS1147	GCA_002961625.1	2.42623	41.3	26118	29	China	Contig	2018/2/28	
1060	SS1148	GCA_002961635.1	2.23507	41.1	110127	31	China	Contig	2018/2/28	
1059	SS1149	GCA_002961645.1	2.24945	41.1	111626	31	China	Contig	2018/2/28	
1053	SS1150	GCA_002961685.1	2.43078	41.3	26611	29	China	Contig	2018/2/28	
1051	SS1151	GCA_002961705.1	2.43332	41.3	26617	29	China	Contig	2018/2/28	
1052	SS1152	GCA_002961715.1	2.42577	41.3	26129	29	China	Contig	2018/2/28	
1050	SS1153	GCA_002961725.1	2.4264	41.3	26765	29	China	Contig	2018/2/28	
1044	SS1154	GCA_002961765.1	2.22735	41	85651	4	China	Contig	2018/2/28	
1034	SS1155	GCA_002961785.1	2.24273	41.1	84501	31	China	Contig	2018/2/28	
1012	SS1156	GCA_002961795.1	2.20209	41.1	63278	16	China	Contig	2018/2/28	
1008	SS1157	GCA_002961825.1	2.50396	41.3	39546	NCL3	China	Contig	2018/2/28	

Strains	ReName	Assembly	Size (Mb)	GC%	N50	Molecular Serotypes	Country	Level	Release Date	Note
970	SS1158	GCA_002961845.1	2.45374	41.3	42865	30	China	Contig	2018/2/28	
969	SS1159	GCA_002961865.1	2.3881	41.3	26636	29	China	Contig	2018/2/28	
965	SS1160	GCA_002961885.1	2.2036	41.1	63351	16	China	Contig	2018/2/28	
963	SS1161	GCA_002961905.1	2.48807	41.3	40618	NCL3	China	Contig	2018/2/28	
944	SS1162	GCA_002961925.1	2.21648	41	85651	4	China	Contig	2018/2/28	
927	SS1163	GCA_002961945.1	2.24336	41.1	88429	31	China	Contig	2018/2/28	
0915-1	SS1164	GCA_002961965.1	2.37786	40.9	123878	2&1/2	China	Contig	2018/2/28	
939	SS1165	GCA_002961975.1	2.26099	41.1	82909	31	China	Contig	2018/2/28	
907	SS1166	GCA_002962005.1	2.18967	40.9	94127	15	China	Contig	2018/2/28	
900	SS1167	GCA_002962025.1	2.26868	41.2	68562	15	China	Contig	2018/2/28	
899	SS1168	GCA_002962045.1	2.21142	41.3	77528	31	China	Contig	2018/2/28	
895	SS1169	GCA_002962065.1	2.21555	41	123985	15	China	Contig	2018/2/28	
893	SS1170	GCA_002962085.1	2.17271	41.1	124012	15	China	Contig	2018/2/28	
890	SS1171	GCA_002962105.1	2.60797	40.8	55588	8	China	Contig	2018/2/28	
869	SS1172	GCA_002962125.1	2.30924	40.9	59861	8	China	Contig	2018/2/28	
889	SS1173	GCA_002962145.1	2.3082	40.9	59861	8	China	Contig	2018/2/28	
874	SS1174	GCA_002962155.1	2.69294	41.1	37318	5	China	Contig	2018/2/28	
859	SS1175	GCA_002962185.1	2.30812	40.9	62438	8	China	Contig	2018/2/28	
849	SS1176	GCA_002962195.1	2.14991	40.9	94287	15	China	Contig	2018/2/28	

Strains	ReName	Assembly	Size (Mb)	GC%	N50	Molecular Serotypes	Country	Level	Release Date	Note
819	SS1177	GCA_002962225.1	2.485	41.3	40913	NCL3	China	Contig	2018/2/28	
812	SS1178	GCA_002962245.1	2.22248	41.2	89056	1&14	China	Contig	2018/2/28	
810	SS1179	GCA_002962265.1	2.21673	41.1	71297	28	China	Contig	2018/2/28	
801	SS1180	GCA_002962295.1	2.34212	41.1	73549	16	China	Contig	2018/2/28	
797	SS1181	GCA_002962305.1	2.55182	41.2	47230	NCL8	China	Contig	2018/2/28	
796	SS1182	GCA_002962345.1	2.53826	41.3	46679	NCL2	China	Contig	2018/2/28	
789	SS1183	GCA_002962365.1	2.23187	41	82837	15	China	Contig	2018/2/28	
786	SS1184	GCA_002962375.1	2.14675	41	71581	15	China	Contig	2018/2/28	
712	SS1185	GCA_002962405.1	2.40907	41.4	48004	NCL7	China	Contig	2018/2/28	
707	SS1186	GCA_002962425.1	2.34499	41.4	52093	NCL7	China	Contig	2018/2/28	
681	SS1188	GCA_002962465.1	2.51869	41.2	42854	30	China	Contig	2018/2/28	
2080	SS1189	GCA_002962515.1	2.44761	41.3	26144	29	China	Contig	2018/2/28	
2070	SS1190	GCA_002962545.1	2.13693	41.2	91673	28	China	Contig	2018/2/28	
2010	SS1191	GCA_002962565.1	2.13699	41.2	95294	28	China	Contig	2018/2/28	
1982	SS1192	GCA_002962595.1	2.64832	41.2	26429	28	China	Contig	2018/2/28	
1783	SS1193	GCA_002962615.1	2.31913	41	56097	16	China	Contig	2018/2/28	
1381	SS1194	GCA_002962665.1	2.11708	41.1	102681	12	China	Contig	2018/2/28	
1372	SS1195	GCA_002962685.1	2.36815	41.5	38582	21	China	Contig	2018/2/28	
1360	SS1196	GCA_002962715.1	2.64276	41	55329	NCL4	China	Contig	2018/2/28	

Strains	ReName	Assembly	Size (Mb)	GC%	N50	Molecular Serotypes	Country	Level	Release Date	Note
1324	SS1197	GCA_002962745.1	2.39311	40.8	52177	16	China	Contig	2018/2/28	
1317	SS1198	GCA_002962785.1	2.22912	41.1	70962	16	China	Contig	2018/2/28	
1263	SS1199	GCA_002962805.1	2.23027	41.1	65758	16	China	Contig	2018/2/28	
1313	SS1200	GCA_002962815.1	2.24246	41.1	52093	16	China	Contig	2018/2/28	
1261	SS1201	GCA_002962845.1	2.37122	40.8	67984	11	China	Contig	2018/2/28	
1262	SS1202	GCA_002962865.1	2.23051	41.1	67766	16	China	Contig	2018/2/28	
1259	SS1203	GCA_002962885.1	2.26345	41	70962	11	China	Contig	2018/2/28	
1194	SS1204	GCA_002962915.1	2.39535	41.3	56211	NCL7	China	Contig	2018/2/28	
1180	SS1205	GCA_002962945.1	2.087	41.1	72996	15	China	Contig	2018/2/28	
2151	SS1206	GCA_002962485.1	2.19086	41.2	124303	not hit	China	Contig	2018/2/28	
2041	SS1207	GCA_002962505.1	2.16627	41.2	82611	28	China	Contig	2018/2/28	
1264	SS1208	GCA_002962765.1	2.2435	41	65657	16	China	Contig	2018/2/28	
1176	SS1209	GCA_002962965.1	2.69916	41.1	54231	NCL8	China	Contig	2018/2/28	
1100	SS1210	GCA_002962975.1	2.08271	41	72093	not hit	China	Contig	2018/2/28	
1165	SS1211	GCA_002963005.1	2.39968	40.9	64886	not hit	China	Contig	2018/2/28	
1069	SS1212	GCA_002963025.1	2.35603	41.2	98903	NCL5	China	Contig	2018/2/28	
1024-1	SS1213	GCA_002963035.1	2.11928	41.2	223156	2&1/2	China	Contig	2018/2/28	
1047	SS1214	GCA_002963065.1	2.37477	41	62379	16	China	Contig	2018/2/28	
966	SS1215	GCA_002963085.1	2.20729	41.1	56098	16	China	Contig	2018/2/28	

Strains	ReName	Assembly	Size (Mb)	GC%	N50	Molecular Serotypes	Country	Level	Release Date	Note
985	SS1216	GCA_002963105.1	2.43644	41.3	26241	29	China	Contig	2018/2/28	
964	SS1217	GCA_002963125.1	2.43699	41.3	28401	29	China	Contig	2018/2/28	
957	SS1218	GCA_002963145.1	2.20966	41.1	56096	16	China	Contig	2018/2/28	
955	SS1219	GCA_002963155.1	2.42676	41.3	24684	29	China	Contig	2018/2/28	
942	SS1220	GCA_002963185.1	2.21856	41	82573	4	China	Contig	2018/2/28	
949	SS1221	GCA_002963205.1	2.53653	41.3	39675	29	China	Contig	2018/2/28	
862	SS1222	GCA_002963215.1	2.30755	40.9	59861	8	China	Contig	2018/2/28	
857	SS1223	GCA_002963245.1	2.30808	40.9	59861	8	China	Contig	2018/2/28	
863	SS1224	GCA_002963265.1	2.30861	40.9	58763	8	China	Contig	2018/2/28	
858	SS1225	GCA_002963295.1	2.30918	40.9	62215	8	China	Contig	2018/2/28	
847	SS1226	GCA_002963325.1	2.59304	40.9	60157	31	China	Contig	2018/2/28	
832	SS1227	GCA_002963345.1	2.10212	41.2	89056	1&14	China	Contig	2018/2/28	
811	SS1228	GCA_002963355.1	2.24886	41.1	71297	28	China	Contig	2018/2/28	
805	SS1229	GCA_002963385.1	2.48565	41.3	35884	5	China	Contig	2018/2/28	
803	SS1230	GCA_002963405.1	2.4198	41.3	42005	5	China	Contig	2018/2/28	
794	SS1231	GCA_002963425.1	2.50815	40.9	47433	NCL17	China	Contig	2018/2/28	
768	SS1232	GCA_002963435.1	2.32671	41.1	75516	16	China	Contig	2018/2/28	
667	SS1233	GCA_002963465.1	2.3397	40.8	106933	15	China	Contig	2018/2/28	
676	SS1234	GCA_002963485.1	2.35615	41.4	51484	NCL7	China	Contig	2018/2/28	

Strains	ReName	Assembly	Size (Mb)	GC%	N50	Molecular Serotypes	Country	Level	Release Date	Note
649	SS1235	GCA_002963505.1	2.55186	41.3	48593	NCL8	China	Contig	2018/2/28	
639	SS1236	GCA_002963525.1	2.39859	41	91324	NCL4	China	Contig	2018/2/28	
635	SS1237	GCA_002963535.1	2.23331	41.1	92914	NCL4	China	Contig	2018/2/28	
629	SS1238	GCA_002963565.1	2.23326	41.1	83242	NCL4	China	Contig	2018/2/28	
673	SS1239	GCA_002963585.1	2.72193	41.2	39127	30	China	Contig	2018/2/28	
0424-1	SS1240	GCA_002963595.1	2.47322	41	65657	16	China	Contig	2018/2/28	
615	SS1241	GCA_002963625.1	2.23504	41.1	83242	NCL4	China	Contig	2018/2/28	
393	SS1242	GCA_002963645.1	2.25062	40.9	110715	12	China	Contig	2018/2/28	
356	SS1243	GCA_002963655.1	2.21156	41	64030	16	China	Contig	2018/2/28	
380	SS1244	GCA_002963685.1	2.52731	40.8	47652	15	China	Contig	2018/2/28	
378	SS1245	GCA_002963695.1	2.63341	41.2	39112	NCL3	China	Contig	2018/2/28	
326	SS1246	GCA_002963705.1	2.26525	40.9	53861	16	China	Contig	2018/2/28	
355	SS1247	GCA_002963745.1	2.21219	41	63971	16	China	Contig	2018/2/28	
217	SS1248	GCA_002963765.1	2.45391	41	43306	16	China	Contig	2018/2/28	
192	SS1249	GCA_002963775.1	2.21889	41	55205	3	China	Contig	2018/2/28	
262	SS1250	GCA_002963805.1	2.33944	41.2	64662	16	China	Contig	2018/2/28	
189	SS1251	GCA_002963825.1	2.49208	41.3	56458	NCL1	China	Contig	2018/2/28	
179	SS1252	GCA_002963845.1	2.22194	41	60393	3	China	Contig	2018/2/28	
191	SS1253	GCA_002963865.1	2.21811	41	60944	3	China	Contig	2018/2/28	

Strains	ReName	Assembly	Size (Mb)	GC%	N50	Molecular Serotypes	Country	Level	Release Date	Note
180	SS1254	GCA_002963885.1	2.25342	41.2	106963	5	China	Contig	2018/2/28	
148	SS1255	GCA_002963905.1	2.15714	41.3	267391	9	China	Contig	2018/2/28	
143	SS1256	GCA_002963925.1	2.18524	41.2	49512	16	China	Contig	2018/2/28	
142	SS1257	GCA_002963945.1	2.18835	41.2	49514	16	China	Contig	2018/2/28	
139	SS1258	GCA_002963965.1	2.18669	41.2	48799	16	China	Contig	2018/2/28	
123	SS1259	GCA_002963985.1	2.32768	41.4	48827	12	China	Contig	2018/2/28	
83	SS1260	GCA_002964005.1	2.15179	41.1	47586	16	China	Contig	2018/2/28	
81	SS1261	GCA_002964015.1	2.18175	41.2	50949	16	China	Contig	2018/2/28	
77	SS1263	GCA_002964055.1	2.19415	41.2	49513	16	China	Contig	2018/2/28	
347	SS1264	GCA_002964085.1	2.17212	41	61985	25	China	Contig	2018/2/28	
324	SS1265	GCA_002964105.1	2.32683	40.9	55861	16	China	Contig	2018/2/28	
350	SS1266	GCA_002964115.1	2.39763	40.9	59087	25	China	Contig	2018/2/28	
293	SS1267	GCA_002964145.1	2.48494	40.8	55834	16	China	Contig	2018/2/28	
183	SS1268	GCA_002964165.1	2.34861	41.1	50034	16	China	Contig	2018/2/28	
207	SS1269	GCA_002964185.1	2.40859	40.9	60923	3	China	Contig	2018/2/28	
166	SS1270	GCA_002964205.1	2.49785	41.3	56854	NCL1	China	Contig	2018/2/28	
138	SS1271	GCA_002964225.1	2.39102	40.9	106873	15	China	Contig	2018/2/28	
82	SS1272	GCA_002964325.1	2.10406	41.4	82848	31	China	Contig	2018/2/28	
73	SS1273	GCA_002964345.1	2.18717	41.2	47282	16	China	Contig	2018/2/28	

Strains	ReName	Assembly	Size (Mb)	GC%	N50	Molecular Serotypes	Country	Level	Release Date	Note
61	SS1274	GCA_002964365.1	2.1052	41.4	82890	31	China	Contig	2018/2/28	
111	SS1275	GCA_002964375.1	2.1462	41.2	49529	16	China	Contig	2018/2/28	
53	SS1276	GCA_002964405.1	2.14766	41.2	47717	16	China	Contig	2018/2/28	
52	SS1277	GCA_002964415.1	2.14511	41.2	48534	16	China	Contig	2018/2/28	
25	SS1278	GCA_002964445.1	2.1438	41.1	48675	16	China	Contig	2018/2/28	
13	SS1279	GCA_002964465.1	2.18549	41.2	54498	16	China	Contig	2018/2/28	
36	SS1280	GCA_002964485.1	2.18259	41.2	50949	16	China	Contig	2018/2/28	
31	SS1281	GCA_002964525.1	2.18956	41.2	55465	16	China	Contig	2018/2/28	
2039	SS1283	GCA_002960765.1	2.15126	41.1	81350	16	China	Contig	2018/2/28	
30	SS1284	GCA_002964505.1	2.14492	41.1	45925	16	China	Contig	2018/2/28	
C2230	SS1056	GCA_003012385.1	2.05959	41.2	170348	2&1/2	China	Scaffold	2018/3/20	
C2227	SS1057	GCA_003012395.1	2.08691	41.2	170267	2&1/2	China	Scaffold	2018/3/20	
C2231	SS1058	GCA_003012425.1	2.09268	41.2	173088	2&1/2	China	Scaffold	2018/3/20	
C2232	SS1059	GCA_003012435.1	2.04681	41.2	144097	2&1/2	China	Scaffold	2018/3/20	
C2229	SS1060	GCA_003012465.1	2.04274	41.2	170443	2&1/2	China	Scaffold	2018/3/20	
C2228	SS1061	GCA_003012485.1	2.03915	41.2	170336	2&1/2	China	Scaffold	2018/3/20	
HN105	SS1054	GCA_003144175.1	2.21748	41.32	2196724	5	China	Complete Genome	2018/5/21	
61	SS1051	GCA_003285225.1	2.13842	41.5	2138420	31	China	Complete Genome	2018/7/2	
1081	SS1052	GCA_003285245.1	2.22809	41.5	2228089	31	China	Complete Genome	2018/7/2	

Strains	ReName	Assembly	Size (Mb)	GC%	N50	Molecular Serotypes	Country	Level	Release Date	Note
SH0104	SS1050	GCA_003288175.1	2.2436	41.2	2243598	2&1/2	China	Complete Genome	2018/7/5	
SH1510	SS1048	GCA_003352225.1	2.21469	41	2214693	4	China	Complete Genome	2018/8/1	
HA1003	SS1049	GCA_003352205.1	2.22918	41.4	2229183	4	China	Complete Genome	2018/8/1	
HN105	SS1029	GCA_004009965.1	2.06034	41.1	69408	2&1/2	China	Scaffold	2019/1/9	
WUSS351	SS1027	GCA_004843685.1	2.28329	41.4	2283286	NCL4	China	Complete Genome	2019/4/24	
WUSS328	SS1021	GCA_004922805.1	2.16684	41.2	82001	not hit	China	Contig	2019/4/29	
WUSS286	SS1022	GCA_004922815.1	2.12379	40.9	65904	28	China	Scaffold	2019/4/29	
WUSS327	SS1023	GCA_004922825.1	2.38429	41.3	46818	17	China	Scaffold	2019/4/29	
WUSS330	SS1024	GCA_004922855.1	2.24999	41	126629	not hit	China	Scaffold	2019/4/29	
WUSS425	SS1026	GCA_004922915.1	2.20858	41.1	155977	NCL20	China	Scaffold	2019/4/29	
4417	SS099	GCA_000440475.1	2.22117	41.1	58579	10	Denmark	Contig	2013/7/25	
8830	SS193	GCA_000440575.1	2.19679	40.8	94065	12	Denmark	Contig	2013/7/25	
5428	SS755	GCA_000440935.1	1.98647	41.4	44491	1&14	Denmark	Contig	2013/7/25	
10581	SS845	GCA_000440235.1	2.47816	41.1	70401	13	Denmark	Contig	2013/7/25	
12814	SS880	GCA_000440295.1	2.0675	41.1	71895	11	Denmark	Contig	2013/7/25	
8074	SS912	GCA_000440535.1	2.05903	41.2	33319	7	Denmark	Contig	2013/7/25	
6407	SS368	GCA_000732355.1	2.29236	41	2292360	4	Denmark	Complete Genome	2014/7/22	
11611	SS472	GCA_000440255.1	2.03893	41.2	130857	2&1/2	Germany	Contig	2013/7/25	
IMT40738	SS1045	GCA_003931695.1	2.45825	41.5	34794	NCL3	Germany	Contig	2018/12/8	

Strains	ReName	Assembly	Size (Mb)	GC%	N50	Molecular Serotypes	Country	Level	Release Date	Note
IMT40201	SS1046	GCA_003931705.1	2.34424	41.5	179911	21	Germany	Contig	2018/12/8	
IMT40343	SS1047	GCA_003931715.1	2.25456	41	155466	8	Germany	Contig	2018/12/8	
SsRC-1	SS1055	GCA_003028635.1	2.09097	41.3	94243	2&1/2	Italy	Contig	2018/4/1	
TANI1	SS647	GCA_002157605.1	2.20766	41.1	76565	5	Japan	Contig	2017/3/10	
Ss1223	SS965	GCA_002406355.1	1.98801	41.3	58727	2&1/2	Madagascar	Scaffold	2017/9/29	
S735	SS056	GCA_000294495.1	1.98089	41.4	1980887	2&1/2	Netherlands	Complete Genome	2012/9/4	
13730	SS101	GCA_000440315.1	1.96124	41.3	60278	1&14	Netherlands	Contig	2013/7/25	
6407	SS245	GCA_000440515.1	2.24827	40.9	72838	4	Netherlands	Contig	2013/7/25	
2651	SS487	GCA_000440435.1	1.98353	41.4	76701	2&1/2	Netherlands	Contig	2013/7/25	
14636	SS602	GCA_000440335.1	2.07957	41.2	84846	8	Netherlands	Contig	2013/7/25	
22083	SS610	GCA_000440395.1	2.02465	41.5	88677	9	Netherlands	Contig	2013/7/25	
11538	SS770	GCA_000440275.1	2.16627	40.9	121316	5	Netherlands	Contig	2013/7/25	
4961	SS820	GCA_000440495.1	2.01661	41.2	47196	3	Netherlands	Contig	2013/7/25	
NCTC 10446	SS837	GCA_000440835.1	2.0146	41.3	86543	15	Netherlands	Contig	2013/7/25	
2524	SS947	GCA_000440415.1	1.88224	41.5	60069	6	Netherlands	Contig	2013/7/25	
2726	SS949	GCA_000440455.1	2.22316	41.1	67004	16	Netherlands	Contig	2013/7/25	
GD-0027	SS006	GCA_900007935.1	2.17719	41.3	120512	16	Netherlands	Scaffold	2016/6/30	
2001171	SS018	GCA_900008865.1	2.15454	41	55630	2&1/2	Netherlands	Scaffold	2016/6/30	
GD-0012	SS044	GCA_900009585.1	2.32558	41	53068	9	Netherlands	Contig	2016/6/30	

Strains	ReName	Assembly	Size (Mb)	GC%	N50	Molecular Serotypes	Country	Level	Release Date	Note
890267	SS073	GCA_900011545.1	2.0439	41.2	170423	2&1/2	Netherlands	Contig	2016/6/30	
GD-0051	SS085	GCA_900010095.1	2.32381	41	64833	9	Netherlands	Contig	2016/6/30	
940430	SS091	GCA_900011205.1	1.96976	41.3	123027	2&1/2	Netherlands	Contig	2016/6/30	
GD-0120	SS097	GCA_900014835.1	2.17602	41	33472	8	Netherlands	Contig	2016/6/30	
2061410	SS098	GCA_900010445.1	2.0372	41.2	223456	2&1/2	Netherlands	Contig	2016/6/30	
GD-0087	SS117	GCA_900010195.1	2.21739	41.2	81556	9	Netherlands	Contig	2016/6/30	
GD-0070	SS123	GCA_900007975.1	2.17587	41.1	38731	7	Netherlands	Contig	2016/6/30	
6555	SS150	GCA_900007855.1	2.02217	41.4	44828	1&14	Netherlands	Contig	2016/6/30	
S3	SS160	GCA_900009645.1	2.04742	41.2	222557	2&1/2	Netherlands	Scaffold	2016/6/30	
GD-0034	SS162	GCA_900009605.1	2.22454	41.1	55519	9	Netherlands	Contig	2016/6/30	
6388	SS166	GCA_900009875.1	2.05534	41.2	223000	1&14	Netherlands	Contig	2016/6/30	
GD-0032	SS167	GCA_900009905.1	2.07425	41.3	67155	1&14	Netherlands	Contig	2016/6/30	
940056	SS171	GCA_900010435.1	2.04247	41.1	222558	2&1/2	Netherlands	Scaffold	2016/6/30	
GD-0065	SS172	GCA_900009185.1	2.05182	41.2	223379	2&1/2	Netherlands	Contig	2016/6/30	
C160	SS174	GCA_900009565.1	2.03887	41.2	223079	1&14	Netherlands	Contig	2016/6/30	
6112	SS178	GCA_900010075.1	2.05209	41.2	223077	1&14	Netherlands	Contig	2016/6/30	
GD-0094	SS200	GCA_900011485.1	1.98877	41.2	39539	7	Netherlands	Contig	2016/6/30	
GD-0076	SS231	GCA_900007985.1	2.21853	41.1	80680	9	Netherlands	Contig	2016/6/30	
GD-0101	SS232	GCA_900013305.1	2.04762	41.2	222899	2&1/2	Netherlands	Contig	2016/6/30	

Strains	ReName	Assembly	Size (Mb)	GC%	N50	Molecular Serotypes	Country	Level	Release Date	Note
9401240	SS233	GCA_900012395.1	2.17418	41.4	364896	9	Netherlands	Contig	2016/6/30	
GD-0025	SS237	GCA_900007925.1	2.04868	41.2	223156	2&1/2	Netherlands	Contig	2016/6/30	
GD-0057	SS241	GCA_900009175.1	2.06896	41.4	139694	4	Netherlands	Contig	2016/6/30	
GD-0107	SS242	GCA_900012665.1	2.03625	41.2	223156	2&1/2	Netherlands	Contig	2016/6/30	
GD-0081	SS254	GCA_900009195.1	2.27435	41.2	63745	9	Netherlands	Scaffold	2016/6/30	
GD-0085	SS258	GCA_900010275.1	2.33914	41.2	77329	9	Netherlands	Contig	2016/6/30	
9501632	SS262	GCA_900009285.1	2.23115	41	108480	8	Netherlands	Contig	2016/6/30	
GD-0007	SS263	GCA_900009315.1	2.32248	41.1	80046	9	Netherlands	Contig	2016/6/30	
9402372	SS268	GCA_900009755.1	2.20831	41.1	62143	9	Netherlands	Contig	2016/6/30	
GD-0098	SS284	GCA_900008015.1	2.10172	41.1	114712	4	Netherlands	Contig	2016/6/30	
2012092	SS288	GCA_900007825.1	2.10111	41.1	71187	2&1/2	Netherlands	Scaffold	2016/6/30	
GD-0015	SS324	GCA_900007915.1	2.18301	41.2	80178	31	Netherlands	Contig	2016/6/30	
931260	SS325	GCA_900009985.1	2.0972	41.1	85940	2&1/2	Netherlands	Contig	2016/6/30	
GD-0114	SS345	GCA_900010285.1	1.98311	41.2	222538	2&1/2	Netherlands	Scaffold	2016/6/30	
C187	SS346	GCA_900007845.1	2.05713	41.1	222584	1&14	Netherlands	Scaffold	2016/6/30	
GD-0050	SS350	GCA_900007945.1	2.31416	41	151448	3	Netherlands	Contig	2016/6/30	
GD-0002	SS363	GCA_900010085.1	2.28142	41.2	64751	9	Netherlands	Contig	2016/6/30	
2000343	SS379	GCA_900013145.1	1.99361	41.3	170420	2&1/2	Netherlands	Contig	2016/6/30	
GD-0077	SS388	GCA_900007995.1	2.03643	41.2	223079	2&1/2	Netherlands	Contig	2016/6/30	

Strains	ReName	Assembly	Size (Mb)	GC%	N50	Molecular Serotypes	Country	Level	Release Date	Note
GD-0004	SS431	GCA_900011035.1	2.34175	41.2	81649	9	Netherlands	Scaffold	2016/6/30	
GD-0010	SS454	GCA_900009885.1	2.04831	41.2	223079	2&1/2	Netherlands	Contig	2016/6/30	
GD-0073	SS471	GCA_900011465.1	2.11441	41.2	111867	4	Netherlands	Contig	2016/6/30	
GD-0067	SS473	GCA_900011045.1	2.05743	41.1	43826	7	Netherlands	Contig	2016/6/30	
2011423	SS475	GCA_900007895.1	2.0358	41.2	223156	2&1/2	Netherlands	Contig	2016/6/30	
GD-0108	SS482	GCA_900013575.1	2.01178	41.4	56843	1&14	Netherlands	Contig	2016/6/30	
2051729	SS496	GCA_900010255.1	2.09858	41.1	77174	2&1/2	Netherlands	Contig	2016/6/30	
GD-0063	SS513	GCA_900011455.1	2.33762	41	174728	3	Netherlands	Contig	2016/6/30	
9402159	SS531	GCA_900007865.1	2.01434	41.4	67154	1&14	Netherlands	Contig	2016/6/30	
950136	SS537	GCA_900009745.1	2.10874	41.1	77079	2&1/2	Netherlands	Contig	2016/6/30	
861160	SS552	GCA_900009155.1	2.09846	41.1	86762	2&1/2	Netherlands	Contig	2016/6/30	
870040	SS553	GCA_900010245.1	1.97752	41.3	222891	2&1/2	Netherlands	Contig	2016/6/30	
971344	SS554	GCA_900009865.1	2.04616	41.2	223079	2&1/2	Netherlands	Contig	2016/6/30	
GD-0018	SS556	GCA_900012315.1	2.20234	41.2	68908	9	Netherlands	Contig	2016/6/30	
OV585	SS558	GCA_900015085.1	1.98212	41.4	54213	1&14	Netherlands	Contig	2016/6/30	
12V457	SS565	GCA_900010565.1	2.14326	41.1	46333	7	Netherlands	Contig	2016/6/30	
2032008	SS572	GCA_900009295.1	2.09442	41.1	70976	2&1/2	Netherlands	Contig	2016/6/30	
2061238	SS581	GCA_900010735.1	2.048	41.2	222780	2&1/2	Netherlands	Contig	2016/6/30	
GD-0008	SS594	GCA_900007905.1	2.12463	41.1	80660	2&1/2	Netherlands	Contig	2016/6/30	

Strains	ReName	Assembly	Size (Mb)	GC%	N50	Molecular Serotypes	Country	Level	Release Date	Note
GD-0023	SS627	GCA_900009165.1	1.99218	41.4	189444	1&14	Netherlands	Contig	2016/6/30	
901131	SS648	GCA_900007875.1	2.02498	41.3	223068	2&1/2	Netherlands	Contig	2016/6/30	
902409	SS654	GCA_900011765.1	1.98498	41.2	223380	2&1/2	Netherlands	Contig	2016/6/30	
GD-0001	SS664	GCA_900007835.1	2.08432	41.2	80511	2&1/2	Netherlands	Contig	2016/6/30	
9406160	SS667	GCA_900011445.1	2.32286	41.1	75480	9	Netherlands	Contig	2016/6/30	
GD-0106	SS668	GCA_900008035.1	2.28385	41.2	75193	9	Netherlands	Scaffold	2016/6/30	
941372	SS671	GCA_900013565.1	2.10534	41.2	69870	2&1/2	Netherlands	Contig	2016/6/30	
GD-0080	SS685	GCA_900009635.1	2.15045	41.2	45617	9	Netherlands	Contig	2016/6/30	
GD-0111	SS693	GCA_900008045.1	2.14633	41.2	59224	9	Netherlands	Contig	2016/6/30	
920694	SS722	GCA_900007885.1	2.10248	41.2	86763	2&1/2	Netherlands	Contig	2016/6/30	
GD-0054	SS726	GCA_900007965.1	2.23703	41.2	64888	9	Netherlands	Contig	2016/6/30	
S10	SS736	GCA_900009205.1	2.01937	41.3	170426	2&1/2	Netherlands	Contig	2016/6/30	
GD-0088	SS739	GCA_900011475.1	2.23698	41.2	64785	9	Netherlands	Contig	2016/6/30	
GD-0021	SS743	GCA_900010455.1	2.15714	41.2	54980	9	Netherlands	Scaffold	2016/6/30	
GD-0031	SS744	GCA_900009765.1	2.05033	41.1	41496	7	Netherlands	Contig	2016/6/30	
GD-0113	SS745	GCA_900008055.1	2.09883	41.2	218876	1&14	Netherlands	Scaffold	2016/6/30	
GD-0006	SS748	GCA_900010265.1	2.3205	41.1	52090	9	Netherlands	Scaffold	2016/6/30	
GD-0079	SS750	GCA_900009785.1	2.27839	41.2	69330	9	Netherlands	Contig	2016/6/30	
GD-0117	SS789	GCA_900013315.1	2.27831	41.2	62947	9	Netherlands	Contig	2016/6/30	

Strains	ReName	Assembly	Size (Mb)	GC%	N50	Molecular Serotypes	Country	Level	Release Date	Note
GD-0112	SS797	GCA_900008065.1	2.26426	41.1	62143	8	Netherlands	Scaffold	2016/6/30	
GD-0100	SS807	GCA_900008025.1	2.14848	41.2	62138	9	Netherlands	Contig	2016/6/30	
GD-0072	SS815	GCA_900009625.1	2.27647	41.1	71687	9	Netherlands	Contig	2016/6/30	
GD-0053	SS819	GCA_900010745.1	2.00902	41.4	67154	1&14	Netherlands	Contig	2016/6/30	
8067	SS831	GCA_900009305.1	2.2525	41.1	71687	9	Netherlands	Contig	2016/6/30	
GD-0061	SS850	GCA_900011215.1	2.15529	41	41534	7	Netherlands	Scaffold	2016/6/30	
2001946	SS859	GCA_900007815.1	2.0375	41.2	223079	2&1/2	Netherlands	Contig	2016/6/30	
GD-0090	SS861	GCA_900011775.1	2.08343	41.2	223456	2&1/2	Netherlands	Contig	2016/6/30	
GD-0052	SS869	GCA_900009615.1	2.1897	41.2	62141	9	Netherlands	Contig	2016/6/30	
GD-0028	SS882	GCA_900009595.1	2.20625	41.1	68716	9	Netherlands	Contig	2016/6/30	
GD-0096	SS887	GCA_900008005.1	2.09792	41.1	54451	2&1/2	Netherlands	Scaffold	2016/6/30	
GD-0049	SS888	GCA_900007955.1	2.02559	41.2	223079	2&1/2	Netherlands	Contig	2016/6/30	
GD-0036	SS920	GCA_900009775.1	2.05384	41.2	223377	1&14	Netherlands	Contig	2016/6/30	
2071319	SS925	GCA_900013295.1	2.04858	41.2	222856	2&1/2	Netherlands	Contig	2016/6/30	
GD-0119	SS928	GCA_900011555.1	2.15454	41	64252	2&1/2	Netherlands	Scaffold	2016/6/30	
971724	SS931	GCA_900009575.1	2.04839	41.2	223379	2&1/2	Netherlands	Contig	2016/6/30	
GD-0013	SS961	GCA_900009895.1	2.04799	41.2	223079	2&1/2	Netherlands	Contig	2016/6/30	
GD-0105	SS966	GCA_900009915.1	2.31309	41.3	19650	17	Netherlands	Contig	2016/6/30	
940255	SS975	GCA_900010555.1	2.09117	41.2	77136	2&1/2	Netherlands	Contig	2016/6/30	

Strains	ReName	Assembly	Size (Mb)	GC%	N50	Molecular Serotypes	Country	Level	Release Date	Note
2150651	SS469	GCA_900177235.1	2.03649	41.2	223523	2&1/2	Netherlands	Contig	2017/7/31	
S10	SS1028	GCA_900642965.1	2.01109	41.3	170355	2&1/2	Netherlands	Contig	2019/3/29	
SS29	SS1030	GCA_003933285.1	1.89445	41.5	61323	6	Switzerland	Contig	2018/12/10	
SS8	SS1031	GCA_003933705.1	1.89352	41.5	61323	6	Switzerland	Contig	2018/12/10	
SS470	SS1032	GCA_003933805.1	2.07989	41.1	94915	2&1/2	Switzerland	Contig	2018/12/10	
PP730	SS1033	GCA_003933815.1	1.91246	41.4	61147	1&14	Switzerland	Contig	2018/12/10	
PP463	SS1034	GCA_003933825.1	2.13545	41.1	94915	2&1/2	Switzerland	Contig	2018/12/10	
SS1014	SS1035	GCA_003933835.1	2.50449	41.2	19299	NCL1	Switzerland	Contig	2018/12/10	
PP425	SS1036	GCA_003933905.1	1.88124	41.5	62213	6	Switzerland	Contig	2018/12/10	
PP203	SS1037	GCA_003933915.1	2.12706	41.4	99943	9	Switzerland	Contig	2018/12/10	
PP269	SS1038	GCA_003933945.1	1.96815	41.3	59708	1&14	Switzerland	Contig	2018/12/10	
PP464	SS1039	GCA_003933955.1	2.34045	41.5	41640	NCL3	Switzerland	Contig	2018/12/10	
PP386	SS1041	GCA_003934345.1	1.87885	41.5	60777	6	Switzerland	Contig	2018/12/10	
PP423	SS1042	GCA_003934375.1	2.06834	41.2	74703	2&1/2	Switzerland	Contig	2018/12/10	
PP735	SS1043	GCA_003934385.1	1.91263	41.4	72418	1&14	Switzerland	Contig	2018/12/10	
PP536	SS1044	GCA_003933845.1	2.12216	41.4	191877	9	Switzerland	Contig	2018/12/10	
P1/7	SS425	GCA_000091905.1	2.00749	41.3	2007491	2&1/2	UK	Complete Genome	2009/7/7	
LL-U	SS021	GCA_000944455.1	2.17698	41	59083	4	UK	Contig	2015/2/14	
LS4M	SS032	GCA_000942215.1	2.14676	41	59312	15	UK	Contig	2015/2/14	

Strains	ReName	Assembly	Size (Mb)	GC%	N50	Molecular Serotypes	Country	Level	Release Date	Note
S11P	SS042	GCA_000945075.1	2.1038	41.1	203654	2&1/2	UK	Scaffold	2015/2/14	
LS0L	SS051	GCA_000938955.1	2.26726	41.2	81553	31	UK	Contig	2015/2/14	
LS6K	SS066	GCA_000937815.1	2.15671	41.3	69373	9	UK	Contig	2015/2/14	
type strain: LS0	SS104	GCA_000946175.1	2.03564	41.3	92786	2&1/2	UK	Contig	2015/2/14	
LS6G	SS107	GCA_000941875.1	2.12415	41.1	57882	2&1/2	UK	Contig	2015/2/14	
LS5Y	SS133	GCA_000945975.1	2.15684	41.4	141968	9	UK	Contig	2015/2/14	
LS3H	SS135	GCA_000939095.1	2.03838	41.2	87995	2&1/2	UK	Contig	2015/2/14	
LS6Z	SS137	GCA_000940415.1	2.15888	41.1	37099	7	UK	Contig	2015/2/14	
type strain: LS4	SS170	GCA_000937395.1	2.15556	41	64537	3	UK	Contig	2015/2/14	
LS1C	SS173	GCA_000936855.1	2.23147	41.4	117441	31	UK	Contig	2015/2/14	
LS1N	SS177	GCA_000945535.1	2.08208	41.1	81683	2&1/2	UK	Contig	2015/2/14	
LL-R	SS199	GCA_000939235.1	2.04745	41.1	92792	1&14	UK	Contig	2015/2/14	
LS3W	SS201	GCA_000877055.1	2.31398	41.3	17250	21	UK	Contig	2015/2/14	
LS6R	SS210	GCA_000946915.1	2.18173	41.1	72283	11	UK	Contig	2015/2/14	
LS1J	SS224	GCA_000877015.1	2.33133	41.3	30154	21	UK	Contig	2015/2/14	
LS4P	SS251	GCA_000937535.1	2.28767	40.8	102569	4	UK	Contig	2015/2/14	
LS5R	SS277	GCA_000946255.1	2.12043	41.1	54568	2&1/2	UK	Contig	2015/2/14	
LS5F	SS291	GCA_000936675.1	2.15885	41.1	38271	7	UK	Contig	2015/2/14	
LS4X	SS310	GCA_000947235.1	2.42688	41.1	29984	NCL10	UK	Contig	2015/2/14	

Strains	ReName	Assembly	Size (Mb)	GC%	N50	Molecular Serotypes	Country	Level	Release Date	Note
LS4Q	SS313	GCA_000942875.1	2.12222	41.1	56795	2&1/2	UK	Contig	2015/2/14	
LS1M	SS321	GCA_000936695.1	1.99261	41.3	50743	6	UK	Contig	2015/2/14	
LS2K	SS351	GCA_000945995.1	2.11621	41.2	66636	16	UK	Contig	2015/2/14	
LS0X	SS355	GCA_000946935.1	2.29128	41.2	92967	31	UK	Contig	2015/2/14	
LS3A	SS377	GCA_000946715.1	2.08096	41.3	175445	4	UK	Contig	2015/2/14	
LS5U	SS386	GCA_000937315.1	2.24172	41.3	153216	31	UK	Contig	2015/2/14	
LS4F	SS398	GCA_000877035.1	2.04974	41.1	91107	1&14	UK	Contig	2015/2/14	
LS1B	SS410	GCA_000938455.1	2.22158	40.9	64856	15	UK	Contig	2015/2/14	
LS7H	SS420	GCA_000942095.1	2.18658	41	53845	2&1/2	UK	Contig	2015/2/14	
S9A	SS438	GCA_000941855.1	2.07135	41.2	170361	2&1/2	UK	Scaffold	2015/2/14	
LS6S	SS446	GCA_000942035.1	2.1218	41.1	41567	2&1/2	UK	Contig	2015/2/14	
LS0C	SS452	GCA_000938075.1	2.14662	41.1	79339	15	UK	Contig	2015/2/14	
LS2N	SS488	GCA_000936655.1	2.14616	41.3	79131	9	UK	Contig	2015/2/14	
LS4B	SS503	GCA_000941115.1	2.1699	41	73791	8	UK	Contig	2015/2/14	
LS2C	SS504	GCA_000945775.1	2.12575	41.3	89874	16	UK	Contig	2015/2/14	
LS2R	SS508	GCA_000945355.1	2.12298	41.1	65025	2&1/2	UK	Contig	2015/2/14	
LS0B	SS525	GCA_000941375.1	2.1825	41.1	71870	NCL4	UK	Contig	2015/2/14	
LS6V	SS527	GCA_000938835.1	2.16943	41	132649	8	UK	Contig	2015/2/14	
LL-S	SS538	GCA_000940675.1	2.24392	41.1	51454	3	UK	Contig	2015/2/14	

Strains	ReName	Assembly	Size (Mb)	GC%	N50	Molecular Serotypes	Country	Level	Release Date	Note
LS2Z	SS563	GCA_000937615.1	2.47058	41.4	39693	24	UK	Contig	2015/2/14	
LS6D	SS566	GCA_000944335.1	2.42965	41.6	38787	NCL7	UK	Contig	2015/2/14	
LS2O	SS595	GCA_000939075.1	2.08276	41.1	144712	2&1/2	UK	Contig	2015/2/14	
LS3E	SS617	GCA_000944375.1	2.15048	41.1	66964	16	UK	Contig	2015/2/14	
LL-Q	SS626	GCA_000937295.1	2.00377	41.3	135491	1&14	UK	Contig	2015/2/14	
LS2V	SS630	GCA_000941215.1	2.47669	41.3	27780	19	UK	Contig	2015/2/14	
LS7A	SS637	GCA_000937935.1	2.25626	41.1	59727	NCL4	UK	Contig	2015/2/14	
LS5J	SS650	GCA_000936615.1	2.12805	41.2	112009	10	UK	Contig	2015/2/14	
LL-Z	SS661	GCA_000942315.1	2.04855	41.1	91476	1&14	UK	Contig	2015/2/14	
LS5C	SS670	GCA_000940735.1	2.03631	41.3	84589	2&1/2	UK	Contig	2015/2/14	
LS3P	SS673	GCA_000937035.1	2.33399	41.2	57193	24	UK	Contig	2015/2/14	
LS2D	SS729	GCA_000941715.1	2.168	41	47928	25	UK	Contig	2015/2/14	
LS5Q	SS771	GCA_000942375.1	2.17685	41	46640	11	UK	Contig	2015/2/14	
LS0I	SS778	GCA_000941315.1	2.05328	41.3	68443	6	UK	Contig	2015/2/14	
LS0E	SS792	GCA_000946095.1	2.15651	41.2	66648	31	UK	Contig	2015/2/14	
LS3S	SS794	GCA_000876795.1	2.15427	41	85541	3	UK	Contig	2015/2/14	
LS2G	SS806	GCA_000945695.1	2.04237	41.2	131413	2&1/2	UK	Contig	2015/2/14	
LS0A	SS812	GCA_000946655.1	2.26764	41.3	72046	31	UK	Contig	2015/2/14	
LS3D	SS832	GCA_000936735.1	2.15858	41.1	38428	7	UK	Contig	2015/2/14	

Strains	ReName	Assembly	Size (Mb)	GC%	N50	Molecular Serotypes	Country	Level	Release Date	Note
LS0M	SS856	GCA_000943075.1	2.27286	40.8	82020	4	UK	Contig	2015/2/14	
LS4I	SS865	GCA_000941495.1	2.37139	41.3	27796	21	UK	Contig	2015/2/14	
type strain: LL-	SS897	GCA_000876755.1	2.16634	41.1	62044	3	UK	Contig	2015/2/14	
LS4E	SS901	GCA_000945795.1	2.15843	41.1	33984	7	UK	Contig	2015/2/14	
LL-P	SS902	GCA_000938915.1	2.03634	41.3	94097	2&1/2	UK	Contig	2015/2/14	
S12Z	SS904	GCA_000877075.1	2.05179	41.1	170354	2&1/2	UK	Scaffold	2015/2/14	
LL-Y	SS908	GCA_000877155.1	2.0529	41.1	80413	1&14	UK	Contig	2015/2/14	
LS1Q	SS922	GCA_000941235.1	2.12427	41.1	65041	2&1/2	UK	Contig	2015/2/14	
LS1F	SS934	GCA_000945875.1	2.12318	41.1	56427	2&1/2	UK	Contig	2015/2/14	
LL-X	SS935	GCA_000942515.1	2.09301	41.1	37144	7	UK	Contig	2015/2/14	
LS0P	SS942	GCA_000945215.1	2.28895	40.9	91231	9	UK	Contig	2015/2/14	
LS1U	SS952	GCA_000944495.1	2.03817	41.2	94097	2&1/2	UK	Contig	2015/2/14	
LS1Y	SS954	GCA_000942015.1	2.16252	41.5	94125	24	UK	Contig	2015/2/14	
LL-W	SS962	GCA_000944755.1	2.19499	41	34781	7	UK	Contig	2015/2/14	
S13G	SS002	GCA_000942335.1	2.22307	41	91241	8	UK	Contig	2015/2/18	
S98Y	SS008	GCA_000939175.1	1.97482	41.3	92782	2&1/2	UK	Contig	2015/2/18	
LS9V	SS013	GCA_000936955.1	2.12026	41.1	40072	2&1/2	UK	Contig	2015/2/18	
S13A	SS027	GCA_000942835.1	2.16515	41	45323	3	UK	Contig	2015/2/18	
S91R	SS028	GCA_000936575.1	2.13516	40.7	92774	2&1/2	UK	Contig	2015/2/18	

Strains	ReName	Assembly	Size (Mb)	GC%	N50	Molecular Serotypes	Country	Level	Release Date	Note
S11K	SS059	GCA_000946235.1	2.16476	40.9	60172	3	UK	Contig	2015/2/18	
S90C	SS063	GCA_000947555.1	2.08298	41.1	144728	2&1/2	UK	Contig	2015/2/18	
LS8Q	SS070	GCA_000944615.1	2.28187	41.2	110552	31	UK	Contig	2015/2/18	
S12R	SS078	GCA_000938155.1	1.96968	41.3	124510	7	UK	Contig	2015/2/18	
S10F	SS089	GCA_000940335.1	2.12414	41.1	66902	2&1/2	UK	Contig	2015/2/18	
S16Z	SS093	GCA_000940575.1	2.11298	41.1	144838	8	UK	Contig	2015/2/18	
LS8F	SS105	GCA_000945455.1	2.34587	41.5	22994	NCL12	UK	Contig	2015/2/18	
S99B	SS116	GCA_000941815.1	2.03909	41.2	122503	2&1/2	UK	Contig	2015/2/18	
S10E	SS118	GCA_000946155.1	2.03051	41.2	88374	2&1/2	UK	Contig	2015/2/18	
S10B	SS140	GCA_000945555.1	2.15876	41	45414	3	UK	Contig	2015/2/18	
S12Y	SS145	GCA_000947775.1	2.08515	41.2	91789	1&14	UK	Contig	2015/2/18	
LS9N	SS153	GCA_000937695.1	2.46722	41.3	38882	31	UK	Contig	2015/2/18	
S17I	SS159	GCA_000946595.1	1.95753	41.3	126235	2&1/2	UK	Contig	2015/2/18	
S12X	SS168	GCA_000938795.1	2.14469	41.3	186458	4	UK	Contig	2015/2/18	
LS7P	SS179	GCA_000944715.1	2.00955	41.4	84114	16	UK	Contig	2015/2/18	
type strain: LS7	SS190	GCA_000941555.1	2.00838	41.5	166790	10	UK	Contig	2015/2/18	
LS9Y	SS195	GCA_000943175.1	2.2184	40.9	54997	15	UK	Contig	2015/2/18	
S15S	SS196	GCA_000945415.1	2.25229	41	118912	3	UK	Contig	2015/2/18	
S11G	SS217	GCA_000938095.1	2.03706	41.2	130825	2&1/2	UK	Contig	2015/2/18	

Strains	ReName	Assembly	Size (Mb)	GC%	N50	Molecular Serotypes	Country	Level	Release Date	Note
S13D	SS227	GCA_000947715.1	2.12921	41.1	66351	2&1/2	UK	Contig	2015/2/18	
LS9U	SS229	GCA_000945515.1	2.39794	41.5	30976	30	UK	Contig	2015/2/18	
S12S	SS244	GCA_000942595.1	2.01044	41.2	170230	2&1/2	UK	Contig	2015/2/18	
S92S	SS246	GCA_000944195.1	2.04207	41.2	94103	2&1/2	UK	Contig	2015/2/18	
S97A	SS283	GCA_000942935.1	2.19459	41	134752	4	UK	Contig	2015/2/18	
S10Y	SS287	GCA_000941655.1	2.03706	41.2	130825	2&1/2	UK	Contig	2015/2/18	
S12W	SS289	GCA_000943855.1	2.04904	41.1	111874	1&14	UK	Contig	2015/2/18	
S13B	SS311	GCA_000938135.1	1.96936	41.3	92782	2&1/2	UK	Contig	2015/2/18	
S14M	SS314	GCA_000947495.1	2.04783	41.2	54215	2&1/2	UK	Contig	2015/2/18	
S13F	SS319	GCA_000938195.1	2.05699	41.1	130824	2&1/2	UK	Contig	2015/2/18	
type strain: LS8	SS331	GCA_000943255.1	2.02777	41.2	87107	2&1/2	UK	Contig	2015/2/18	
S14L	SS334	GCA_000938215.1	2.16567	41	75742	4	UK	Contig	2015/2/18	
S10W	SS336	GCA_000935945.1	2.13884	41	35791	7	UK	Contig	2015/2/18	
S96O	SS354	GCA_000943215.1	2.18983	41	62831	12	UK	Contig	2015/2/18	
S15U	SS357	GCA_000936385.1	1.98057	41.2	88815	2&1/2	UK	Contig	2015/2/18	
S94U	SS361	GCA_000946215.1	1.96761	41.3	88374	2&1/2	UK	Contig	2015/2/18	
LS7W	SS378	GCA_000942135.1	2.12235	41.1	60110	2&1/2	UK	Contig	2015/2/18	
LSK	SS414	GCA_000937555.1	2.15609	41.2	64339	16	UK	Contig	2015/2/18	
S12U	SS422	GCA_000942995.1	2.03703	41.2	92766	2&1/2	UK	Contig	2015/2/18	

Strains	ReName	Assembly	Size (Mb)	GC%	N50	Molecular Serotypes	Country	Level	Release Date	Note
LS8X	SS427	GCA_000940275.1	2.11669	41.2	68003	12	UK	Contig	2015/2/18	
LSS	SS428	GCA_000937215.1	2.0297	41.3	96988	2&1/2	UK	Contig	2015/2/18	
S15V	SS432	GCA_000947415.1	2.03701	41.2	92762	2&1/2	UK	Contig	2015/2/18	
S10D	SS447	GCA_000936325.1	2.12639	41	37120	7	UK	Contig	2015/2/18	
S11N	SS448	GCA_000943115.1	2.08296	41.1	98056	2&1/2	UK	Contig	2015/2/18	
S10A	SS458	GCA_000938415.1	2.03136	41.3	131078	2&1/2	UK	Contig	2015/2/18	
S11H	SS463	GCA_000936815.1	2.04855	41.1	170328	1&14	UK	Contig	2015/2/18	
S10X	SS470	GCA_000942795.1	2.03738	41.2	83766	2&1/2	UK	Contig	2015/2/18	
LS8I	SS486	GCA_000947535.1	2.38894	41.4	31962	NCL3	UK	Contig	2015/2/18	
S16A	SS491	GCA_000946835.1	2.14586	41	53511	15	UK	Contig	2015/2/18	
LS7E	SS499	GCA_000936025.1	2.33848	41.1	70653	24	UK	Contig	2015/2/18	
type strain: S93	SS505	GCA_000943055.1	2.29001	41.5	36825	NCL11	UK	Contig	2015/2/18	
S96H	SS522	GCA_000941295.1	2.04346	41.2	92766	2&1/2	UK	Contig	2015/2/18	
LS9R	SS523	GCA_000943315.1	2.24223	41.2	97885	NCL20	UK	Contig	2015/2/18	
S93E	SS524	GCA_000946495.1	2.03777	41.2	91543	2&1/2	UK	Contig	2015/2/18	
S97P	SS539	GCA_000942635.1	2.051	41.1	130821	1&14	UK	Contig	2015/2/18	
S99Z	SS545	GCA_000945495.1	2.26037	41	82937	3	UK	Contig	2015/2/18	
S13C	SS559	GCA_000942075.1	2.04933	41.1	170227	1&14	UK	Contig	2015/2/18	
S91K	SS567	GCA_000942655.1	2.02018	41.5	97214	9	UK	Contig	2015/2/18	

Strains	ReName	Assembly	Size (Mb)	GC%	N50	Molecular Serotypes	Country	Level	Release Date	Note
S15X	SS576	GCA_000946135.1	2.15611	41	40834	3	UK	Contig	2015/2/18	
S92D	SS583	GCA_000946695.1	2.04285	41.2	130821	2&1/2	UK	Contig	2015/2/18	
LSV	SS586	GCA_000937335.1	2.087	41.3	50912	10	UK	Contig	2015/2/18	
LS8J	SS587	GCA_000938555.1	1.99757	41.4	60495	16	UK	Contig	2015/2/18	
S16B	SS605	GCA_000945595.1	2.03681	41.2	130821	2&1/2	UK	Contig	2015/2/18	
LS9G	SS609	GCA_000946615.1	2.21029	41.3	53551	31	UK	Contig	2015/2/18	
S14K	SS612	GCA_000947055.1	2.16588	41	100466	4	UK	Contig	2015/2/18	
S11M	SS651	GCA_000944535.1	2.19496	40.9	172029	8	UK	Contig	2015/2/18	
S96W	SS658	GCA_000945155.1	2.1631	41.1	66898	2&1/2	UK	Contig	2015/2/18	
LSL	SS665	GCA_000938495.1	2.12216	41.1	65021	2&1/2	UK	Contig	2015/2/18	
S16E	SS682	GCA_000936045.1	1.96454	41.3	110942	2&1/2	UK	Contig	2015/2/18	
S97X	SS687	GCA_000940175.1	2.17058	41.4	85017	8	UK	Contig	2015/2/18	
LS8B	SS697	GCA_000944175.1	2.21894	41.2	119997	31	UK	Contig	2015/2/18	
S14J	SS703	GCA_000937175.1	2.16619	41	75133	4	UK	Contig	2015/2/18	
LS7L	SS704	GCA_000937655.1	2.22268	41	114808	8	UK	Contig	2015/2/18	
S95V	SS708	GCA_000945035.1	2.05057	41.1	130825	1&14	UK	Contig	2015/2/18	
S99I	SS717	GCA_000947435.1	2.05607	41.1	94101	1&14	UK	Contig	2015/2/18	
S12Q	SS725	GCA_000937875.1	2.07765	41.2	49207	3	UK	Contig	2015/2/18	
LS9C	SS735	GCA_000937915.1	2.12939	41.3	128567	10	UK	Contig	2015/2/18	

Strains	ReName	Assembly	Size (Mb)	GC%	N50	Molecular Serotypes	Country	Level	Release Date	Note
S17G	SS747	GCA_000942055.1	2.04014	41.2	88845	2&1/2	UK	Contig	2015/2/18	
S94F	SS749	GCA_000944215.1	2.16081	41.1	74642	2&1/2	UK	Contig	2015/2/18	
LS8M	SS756	GCA_000939915.1	2.22183	41	105931	8	UK	Contig	2015/2/18	
LSW	SS757	GCA_000939375.1	2.13365	41.2	84594	15	UK	Contig	2015/2/18	
S17H	SS769	GCA_000941515.1	1.98119	41.2	92782	2&1/2	UK	Contig	2015/2/18	
S95G	SS774	GCA_000947455.1	2.27983	41.2	76518	NCL20	UK	Contig	2015/2/18	
LS8U	SS781	GCA_000936515.1	2.03769	41.2	91551	2&1/2	UK	Contig	2015/2/18	
S95N	SS782	GCA_000937775.1	2.15582	41.1	54095	NCL6	UK	Contig	2015/2/18	
S16C	SS784	GCA_000945135.1	2.06341	41.1	27567	2&1/2	UK	Contig	2015/2/18	
S12V	SS800	GCA_000943295.1	2.03704	41.2	130821	2&1/2	UK	Contig	2015/2/18	
type strain: S12	SS801	GCA_000937735.1	1.99727	41.3	94100	2&1/2	UK	Contig	2015/2/18	
S16D	SS830	GCA_000939015.1	2.03783	41.2	92184	2&1/2	UK	Contig	2015/2/18	
S11I	SS833	GCA_000942855.1	2.07746	41.1	97246	2&1/2	UK	Contig	2015/2/18	
S15W	SS839	GCA_000941275.1	2.09241	41.5	168281	8	UK	Contig	2015/2/18	
LS7S	SS841	GCA_000944895.1	2.02577	41.2	92774	2&1/2	UK	Contig	2015/2/18	
S11J	SS842	GCA_000943955.1	2.42334	41.4	30817	not hit	UK	Contig	2015/2/18	
LSH	SS854	GCA_000946195.1	2.12347	41.1	74646	2&1/2	UK	Contig	2015/2/18	
LSD	SS857	GCA_000945675.1	2.03067	41.3	101805	2&1/2	UK	Contig	2015/2/18	
S13H	SS870	GCA_000945015.1	2.06336	41.1	92764	1&14	UK	Contig	2015/2/18	

Strains	ReName	Assembly	Size (Mb)	GC%	N50	Molecular Serotypes	Country	Level	Release Date	Note
S11L	SS878	GCA_000937075.1	2.20664	40.9	65989	3	UK	Contig	2015/2/18	
S15Y	SS889	GCA_000938675.1	1.98734	41.3	170227	1&14	UK	Contig	2015/2/18	
S13E	SS892	GCA_000938115.1	2.10787	41.1	58411	8	UK	Contig	2015/2/18	
S10C	SS911	GCA_000943035.1	2.03763	41.2	130833	2&1/2	UK	Contig	2015/2/18	
S14O	SS917	GCA_000947695.1	2.15415	40.9	114804	4	UK	Contig	2015/2/18	
S16F	SS924	GCA_000936285.1	2.10146	41.1	72897	16	UK	Contig	2015/2/18	
S93M	SS945	GCA_000936305.1	2.02799	41.2	92766	1&14	UK	Contig	2015/2/18	
S13I	SS948	GCA_000936795.1	2.04823	41.1	130952	1&14	UK	Contig	2015/2/18	
type strain: S15	SS957	GCA_000944115.1	2.06676	41.1	30475	2&1/2	UK	Contig	2015/2/18	
S99Q	SS973	GCA_000945935.1	2.12349	41.1	52090	2&1/2	UK	Contig	2015/2/18	
S11O	SS067	GCA_000939715.1	2.04677	41.1	30192	7	UK	Contig	2015/2/20	
S15R	SS297	GCA_000936145.1	2.08513	41.1	28464	7	UK	Contig	2015/2/20	
S92L	SS844	GCA_000940755.1	2.04857	41.1	92758	1&14	UK	Contig	2015/2/20	
LS9J	SS896	GCA_000936915.1	2.09594	41.3	155306	8	UK	Contig	2015/2/24	
LOLA-SS003	SS003	GCA_900065705.1	2.04169	41.1	167760	1&14	UK	Scaffold	2016/2/10	
LSS1	SS007	GCA_900048825.1	2.11432	41.1	74701	2&1/2	UK	Contig	2016/2/10	
LSS8	SS010	GCA_900052525.1	2.14941	41.2	70677	16	UK	Contig	2016/2/10	
LSS41	SS014	GCA_900065745.1	2.31794	41.2	49151	21	UK	Contig	2016/2/10	
SS1026	SS015	GCA_900065765.1	2.03172	41.1	170245	2&1/2	UK	Contig	2016/2/10	

Strains	ReName	Assembly	Size (Mb)	GC%	N50	Molecular Serotypes	Country	Level	Release Date	Note
LSS80	SS016	GCA_900057045.1	2.25646	41.2	107540	31	UK	Contig	2016/2/10	
LSS85	SS023	GCA_900060955.1	2.15435	41	39425	7	UK	Contig	2016/2/10	
LSS68	SS025	GCA_900052825.1	2.21589	41	140264	8	UK	Contig	2016/2/10	
LSS67	SS026	GCA_900056495.1	2.21912	41	181574	8	UK	Contig	2016/2/10	
LSS90	SS037	GCA_900062275.1	2.26058	41.2	162315	31	UK	Contig	2016/2/10	
LSS82	SS039	GCA_900056525.1	2.11681	41.3	139043	16	UK	Contig	2016/2/10	
LSS23	SS040	GCA_900056165.1	2.32272	41.2	67073	24	UK	Contig	2016/2/10	
LSS60	SS043	GCA_900048835.1	2.14875	41.2	143800	31	UK	Contig	2016/2/10	
LSS42	SS046	GCA_900049345.1	2.1068	41.2	104045	16	UK	Contig	2016/2/10	
SS981	SS050	GCA_900052535.1	2.0021	41.4	111300	9	UK	Contig	2016/2/10	
LSS65	SS054	GCA_900050815.1	2.12126	41.2	151599	10	UK	Contig	2016/2/10	
SS1010	SS061	GCA_900059135.1	2.03214	41.2	233641	2&1/2	UK	Contig	2016/2/10	
LSS32	SS065	GCA_900063575.1	2.45035	41.4	67436	24	UK	Scaffold	2016/2/10	
SS1004	SS068	GCA_900062845.1	2.02344	41.3	222443	2&1/2	UK	Contig	2016/2/10	
SS1033	SS072	GCA_900049975.1	2.04259	41.1	233805	1&14	UK	Contig	2016/2/10	
LSS37	SS079	GCA_900063595.1	2.33017	41.1	148011	24	UK	Contig	2016/2/10	
SS1015	SS081	GCA_900055125.1	2.19359	40.9	53722	3	UK	Contig	2016/2/10	
LSS81	SS087	GCA_900058005.1	2.21537	40.9	102765	15	UK	Contig	2016/2/10	
LSS28	SS095	GCA_900060325.1	2.03479	41.2	170261	2&1/2	UK	Contig	2016/2/10	

Strains	ReName	Assembly	Size (Mb)	GC%	N50	Molecular Serotypes	Country	Level	Release Date	Note
LSS86	SS110	GCA_900058705.1	2.11848	41.1	95379	2&1/2	UK	Contig	2016/2/10	
LSS63	SS115	GCA_900059895.1	2.03214	41.2	130954	2&1/2	UK	Contig	2016/2/10	
LSS59	SS124	GCA_900058255.1	2.12156	41.3	164538	10	UK	Contig	2016/2/10	
LSS55	SS128	GCA_900053135.1	2.14802	41.4	177979	9	UK	Contig	2016/2/10	
SS1055	SS129	GCA_900051265.1	2.03092	41.2	222522	2&1/2	UK	Scaffold	2016/2/10	
SS975	SS130	GCA_900061355.1	2.24947	41.2	88210	NCL20	UK	Scaffold	2016/2/10	
SS1013	SS136	GCA_900054825.1	2.366	41.3	25756	not hit	UK	Scaffold	2016/2/10	
SS1043	SS148	GCA_900062095.1	2.0362	41.2	68417	2&1/2	UK	Contig	2016/2/10	
SS999	SS152	GCA_900064025.1	2.24764	41	85540	3	UK	Scaffold	2016/2/10	
LSS78	SS154	GCA_900056505.1	2.11086	41.2	104396	12	UK	Contig	2016/2/10	
SS1027	SS156	GCA_900065025.1	2.04147	41.1	130987	1&14	UK	Contig	2016/2/10	
SS972	SS164	GCA_900065785.1	2.03159	41.2	170180	2&1/2	UK	Contig	2016/2/10	
LSS10	SS180	GCA_900057495.1	2.17021	41.1	92065	NCL4	UK	Scaffold	2016/2/10	
SS1029	SS182	GCA_900050405.1	2.07757	41.2	170274	1&14	UK	Contig	2016/2/10	
SS1036	SS183	GCA_900059445.1	2.05111	41.1	170334	2&1/2	UK	Contig	2016/2/10	
LSS84	SS186	GCA_900058025.1	2.15485	41.1	45478	7	UK	Contig	2016/2/10	
LSS14	SS187	GCA_900057985.1	2.04746	41.1	222084	1&14	UK	Contig	2016/2/10	
SS1034	SS188	GCA_900056175.1	2.11734	41.1	82467	2&1/2	UK	Contig	2016/2/10	
LSS40	SS191	GCA_900064015.1	2.04796	41.2	91362	6	UK	Contig	2016/2/10	

Strains	ReName	Assembly	Size (Mb)	GC%	N50	Molecular Serotypes	Country	Level	Release Date	Note
LSS2	SS192	GCA_900062245.1	2.12744	41.2	126349	15	UK	Contig	2016/2/10	
LSS72	SS202	GCA_900049945.1	2.11647	41.1	82401	2&1/2	UK	Contig	2016/2/10	
SS1064	SS204	GCA_900051585.1	2.04763	41.1	30168	2&1/2	UK	Contig	2016/2/10	
SS991	SS206	GCA_900061385.1	2.12831	40.7	160571	2&1/2	UK	Contig	2016/2/10	
LSS83	SS208	GCA_900058015.1	2.15671	41	46530	7	UK	Contig	2016/2/10	
LSS50	SS209	GCA_900054555.1	2.02616	41.2	222078	2&1/2	UK	Contig	2016/2/10	
SS1021	SS215	GCA_900056855.1	2.06282	41.1	64509	3	UK	Contig	2016/2/10	
SS976	SS216	GCA_900061365.1	2.03085	41.2	153580	2&1/2	UK	Contig	2016/2/10	
SS992	SS222	GCA_900050435.1	2.03335	41.2	98875	2&1/2	UK	Contig	2016/2/10	
LSS5	SS230	GCA_900054545.1	2.02598	41.3	222461	2&1/2	UK	Contig	2016/2/10	
LSS75	SS249	GCA_900049955.1	2.23207	41.3	164576	31	UK	Contig	2016/2/10	
SS970	SS252	GCA_900056535.1	2.07639	41.1	93621	2&1/2	UK	Contig	2016/2/10	
LSS30	SS260	GCA_900063555.1	2.28195	41.2	185295	31	UK	Contig	2016/2/10	
SS1001	SS261	GCA_900064665.1	2.0313	41.2	116216	2&1/2	UK	Contig	2016/2/10	
SS1030	SS265	GCA_900049965.1	2.15278	41	50197	3	UK	Contig	2016/2/10	
SS989	SS272	GCA_900050855.1	2.11198	41.1	64512	2&1/2	UK	Contig	2016/2/10	
LOLA-SS001	SS275	GCA_900054285.1	2.02421	41.2	221961	2&1/2	UK	Scaffold	2016/2/10	
SS987	SS276	GCA_900050845.1	2.04711	41.1	170271	1&14	UK	Scaffold	2016/2/10	
SS1008	SS285	GCA_900049645.1	2.02325	41.2	171400	2&1/2	UK	Contig	2016/2/10	

Strains	ReName	Assembly	Size (Mb)	GC%	N50	Molecular Serotypes	Country	Level	Release Date	Note
SS1053	SS293	GCA_900063275.1	2.05248	41.1	32380	2&1/2	UK	Contig	2016/2/10	
SS1005	SS298	GCA_900059125.1	2.13827	41	49027	3	UK	Contig	2016/2/10	
LSS71	SS301	GCA_900051575.1	2.11927	41.1	95400	2&1/2	UK	Contig	2016/2/10	
LSS58	SS312	GCA_900058695.1	2.20633	41.2	126823	31	UK	Scaffold	2016/2/10	
LSS35	SS320	GCA_900051935.1	2.0254	41.2	234070	2&1/2	UK	Contig	2016/2/10	
LSS34	SS323	GCA_900063585.1	2.16074	41	206765	8	UK	Contig	2016/2/10	
LSS92	SS328	GCA_900064165.1	2.13842	41.3	113772	9	UK	Contig	2016/2/10	
LSS61	SS333	GCA_900059095.1	2.11545	41.1	82355	2&1/2	UK	Contig	2016/2/10	
SS1067	SS339	GCA_900052235.1	2.09582	41.1	111390	16	UK	Contig	2016/2/10	
LSS47	SS342	GCA_900055485.1	1.99792	41.4	109839	16	UK	Contig	2016/2/10	
SS973	SS348	GCA_900056545.1	2.03192	41.2	187511	2&1/2	UK	Contig	2016/2/10	
LSS44	SS349	GCA_900053595.1	2.13481	41	76961	15	UK	Contig	2016/2/10	
LOLA-SS009	SS353	GCA_900058685.1	2.08163	41.1	38465	7	UK	Contig	2016/2/10	
SS1011	SS366	GCA_900054105.1	2.04392	41.1	223844	1&14	UK	Contig	2016/2/10	
LSS69	SS384	GCA_900060645.1	2.44853	41.3	63483	31	UK	Contig	2016/2/10	
LSS100	SS401	GCA_900059075.1	2.12986	41.1	81672	15	UK	Contig	2016/2/10	
LSS26	SS407	GCA_900061895.1	2.11417	41.1	74687	2&1/2	UK	Contig	2016/2/10	
SS1037	SS409	GCA_900059455.1	2.21335	41	137336	8	UK	Contig	2016/2/10	
LSS97	SS418	GCA_900062285.1	2.01966	41.2	169676	2&1/2	UK	Contig	2016/2/10	

Strains	ReName	Assembly	Size (Mb)	GC%	N50	Molecular Serotypes	Country	Level	Release Date	Note
LSS57	SS426	GCA_900058475.1	2.24455	41.1	104731	NCL4	UK	Contig	2016/2/10	
SS979	SS433	GCA_900051595.1	2.04431	41.1	222526	1&14	UK	Contig	2016/2/10	
SS997	SS435	GCA_900053165.1	2.13797	41.3	186798	8	UK	Scaffold	2016/2/10	
LOLA-SS004	SS436	GCA_900065715.1	2.22624	41	57087	3	UK	Contig	2016/2/10	
SS1057	SS439	GCA_900052225.1	2.13728	41	44314	3	UK	Contig	2016/2/10	
SS1041	SS453	GCA_900059155.1	2.15606	41	120681	4	UK	Contig	2016/2/10	
SS1032	SS457	GCA_900063615.1	1.96337	41.3	185487	2&1/2	UK	Contig	2016/2/10	
SS1028	SS466	GCA_900050395.1	2.11879	41.3	177652	4	UK	Contig	2016/2/10	
LSS25	SS468	GCA_900059755.1	2.11461	41.1	95281	2&1/2	UK	Contig	2016/2/10	
SS998	SS474	GCA_900053175.1	1.96227	41.3	135152	2&1/2	UK	Contig	2016/2/10	
SS1016	SS483	GCA_900056845.1	2.18354	40.9	188670	8	UK	Contig	2016/2/10	
SS967	SS493	GCA_900049985.1	2.18499	41	130646	4	UK	Contig	2016/2/10	
SS1023	SS500	GCA_900058035.1	2.00312	41.2	170271	2&1/2	UK	Contig	2016/2/10	
SS1062	SS501	GCA_900050835.1	2.11969	41	85386	15	UK	Contig	2016/2/10	
SS1040	SS507	GCA_900059145.1	2.15885	41	100051	4	UK	Scaffold	2016/2/10	
SS1039	SS512	GCA_900059775.1	2.04113	41.1	170817	1&14	UK	Contig	2016/2/10	
SS1065	SS514	GCA_900064675.1	2.03168	41.2	171217	2&1/2	UK	Contig	2016/2/10	
LSS98	SS515	GCA_900062075.1	2.01933	41.2	169682	2&1/2	UK	Contig	2016/2/10	
SS1014	SS516	GCA_900055115.1	2.15091	40.9	61094	3	UK	Scaffold	2016/2/10	

Strains	ReName	Assembly	Size (Mb)	GC%	N50	Molecular Serotypes	Country	Level	Release Date	Note
SS1038	SS518	GCA_900059765.1	2.05428	41.1	108676	1&14	UK	Contig	2016/2/10	
SS995	SS519	GCA_900054845.1	2.04585	41.1	117267	1&14	UK	Contig	2016/2/10	
SS1012	SS528	GCA_900054815.1	2.07092	41.1	170351	2&1/2	UK	Contig	2016/2/10	
SS1025	SS530	GCA_900054575.1	2.03264	41.2	170171	2&1/2	UK	Contig	2016/2/10	
LSS53	SS532	GCA_900057995.1	2.28444	41.2	35461	21	UK	Contig	2016/2/10	
SS1070	SS543	GCA_900065045.1	2.03165	41.2	130977	2&1/2	UK	Scaffold	2016/2/10	
SS1002	SS546	GCA_900059115.1	2.03227	41.2	130836	2&1/2	UK	Contig	2016/2/10	
LSS39	SS551	GCA_900057675.1	2.19512	41.2	88665	31	UK	Contig	2016/2/10	
LSS73	SS570	GCA_900064985.1	2.16053	40.9	94035	3	UK	Contig	2016/2/10	
LSS89	SS577	GCA_900059105.1	2.09233	41.3	179760	8	UK	Contig	2016/2/10	
SS993	SS579	GCA_900055865.1	2.24466	41.4	38289	NCL11	UK	Scaffold	2016/2/10	
LSS70	SS580	GCA_900055855.1	2.28541	40.9	112436	9	UK	Contig	2016/2/10	
SS996	SS582	GCA_900053155.1	2.15053	41.1	64156	2&1/2	UK	Contig	2016/2/10	
LSS4	SS584	GCA_900063605.1	2.02575	41.3	233709	2&1/2	UK	Contig	2016/2/10	
LSS74	SS592	GCA_900064995.1	2.14982	41	125781	3	UK	Contig	2016/2/10	
LSS38	SS606	GCA_900056835.1	2.31851	41.5	48174	NCL12	UK	Scaffold	2016/2/10	
SS1018	SS614	GCA_900065015.1	2.03313	41.1	32030	7	UK	Contig	2016/2/10	
SS969	SS619	GCA_900054835.1	2.0316	41.1	153585	2&1/2	UK	Contig	2016/2/10	
SS1073	SS620	GCA_900053145.1	1.95198	41.3	130778	2&1/2	UK	Contig	2016/2/10	

Strains	ReName	Assembly	Size (Mb)	GC%	N50	Molecular Serotypes	Country	Level	Release Date	Note
LSS51	SS636	GCA_900054565.1	2.02855	41.2	222146	2&1/2	UK	Contig	2016/2/10	
LSS36	SS639	GCA_900049625.1	2.40554	41.6	47243	NCL7	UK	Contig	2016/2/10	
SS1054	SS645	GCA_900050825.1	1.97394	41.2	223535	2&1/2	UK	Contig	2016/2/10	
SS982	SS646	GCA_900050425.1	2.04253	41.1	118089	1&14	UK	Contig	2016/2/10	
LSS22	SS652	GCA_900050785.1	2.07655	41.1	144742	2&1/2	UK	Contig	2016/2/10	
SS1022	SS653	GCA_900057055.1	1.96335	41.3	160074	7	UK	Contig	2016/2/10	
LSS91	SS657	GCA_900061905.1	1.98086	41.3	76024	6	UK	Contig	2016/2/10	
LSS9	SS672	GCA_900058485.1	2.08025	41.2	82352	10	UK	Scaffold	2016/2/10	
SS974	SS677	GCA_900061345.1	2.15152	41.1	80476	2&1/2	UK	Contig	2016/2/10	
LSS94	SS678	GCA_900064295.1	2.28388	40.8	123925	4	UK	Contig	2016/2/10	
SS1000	SS686	GCA_900065235.1	2.1223	41	40065	7	UK	Contig	2016/2/10	
LSS99	SS688	GCA_900062835.1	2.36056	41.4	34609	30	UK	Scaffold	2016/2/10	
SS1035	SS691	GCA_900058495.1	2.09817	41.1	73586	8	UK	Contig	2016/2/10	
LSS3	SS695	GCA_900063545.1	2.11822	41.1	89187	2&1/2	UK	Contig	2016/2/10	
LSS76	SS699	GCA_900060655.1	2.16601	41	196373	8	UK	Contig	2016/2/10	
LSS95	SS700	GCA_900062065.1	2.16656	41	72284	11	UK	Contig	2016/2/10	
SS1058	SS705	GCA_900051955.1	1.98004	41.3	170257	1&14	UK	Contig	2016/2/10	
LSS77	SS707	GCA_900065755.1	2.116	41.1	81800	2&1/2	UK	Contig	2016/2/10	
SS1006	SS710	GCA_900049635.1	2.03342	41.2	168149	2&1/2	UK	Contig	2016/2/10	

Strains	ReName	Assembly	Size (Mb)	GC%	N50	Molecular Serotypes	Country	Level	Release Date	Note
LSS48	SS711	GCA_900055495.1	2.27286	41.2	151335	31	UK	Contig	2016/2/10	
LSS29	SS712	GCA_900052515.1	2.11248	41.1	80470	2&1/2	UK	Contig	2016/2/10	
LSS18	SS727	GCA_900062235.1	1.99035	41.4	110161	16	UK	Scaffold	2016/2/10	
LSS27	SS730	GCA_900062265.1	2.00224	41.5	309469	10	UK	Contig	2016/2/10	
LSS88	SS741	GCA_900058715.1	2.36735	41.3	39019	NCL3	UK	Scaffold	2016/2/10	
LSS33	SS763	GCA_900051565.1	2.07531	41.3	495337	4	UK	Contig	2016/2/10	
SS1063	SS768	GCA_900050415.1	2.03262	41.1	156128	2&1/2	UK	Contig	2016/2/10	
LSS13	SS776	GCA_900059425.1	2.13684	41.1	98534	16	UK	Contig	2016/2/10	
LOLA-SS010	SS780	GCA_900065735.1	2.04717	41.1	222092	1&14	UK	Contig	2016/2/10	
SS1042	SS787	GCA_900062085.1	2.15956	41	86421	4	UK	Contig	2016/2/10	
LSS87	SS788	GCA_900048845.1	2.18041	41	81843	2&1/2	UK	Contig	2016/2/10	
LSS96	SS790	GCA_900058725.1	2.17299	41.1	80919	11	UK	Contig	2016/2/10	
SS1007	SS795	GCA_900051255.1	2.10826	41	34026	7	UK	Contig	2016/2/10	
SS1066	SS808	GCA_900065775.1	1.95772	41.3	221977	2&1/2	UK	Contig	2016/2/10	
LOLA-SS002	SS811	GCA_900054295.1	1.99352	41.3	222083	1&14	UK	Contig	2016/2/10	
SS1024	SS817	GCA_900059435.1	1.98523	41.3	170237	2&1/2	UK	Contig	2016/2/10	
LSS62	SS818	GCA_900058265.1	2.03346	41.2	233999	2&1/2	UK	Contig	2016/2/10	
SS1072	SS822	GCA_900059165.1	1.97694	41.2	130767	2&1/2	UK	Contig	2016/2/10	
LSS12	SS829	GCA_900059415.1	2.16132	41	58674	25	UK	Contig	2016/2/10	

Strains	ReName	Assembly	Size (Mb)	GC%	N50	Molecular Serotypes	Country	Level	Release Date	Note
SS1051	SS834	GCA_900065035.1	2.07236	41.1	32031	7	UK	Contig	2016/2/10	
LSS66	SS848	GCA_900056485.1	2.15038	41.3	135990	9	UK	Contig	2016/2/10	
SS983	SS849	GCA_900056555.1	2.02003	41.2	130697	1&14	UK	Contig	2016/2/10	
SS1048	SS855	GCA_900062295.1	2.14373	40.9	146261	4	UK	Contig	2016/2/10	
SS1009	SS858	GCA_900058735.1	2.11279	41.1	80506	2&1/2	UK	Contig	2016/2/10	
LSS56	SS863	GCA_900059085.1	2.15594	41	46611	7	UK	Contig	2016/2/10	
LOLA-SS005	SS864	GCA_900057485.1	2.15939	41	67171	3	UK	Contig	2016/2/10	
LOLA-SS008	SS867	GCA_900065725.1	2.18123	41	38456	7	UK	Contig	2016/2/10	
LSS11	SS871	GCA_900060635.1	2.22587	41.3	153744	31	UK	Contig	2016/2/10	
LSS52	SS873	GCA_900058245.1	2.45826	41.3	38787	19	UK	Contig	2016/2/10	
LSS21	SS885	GCA_900062255.1	2.07691	41.1	146406	2&1/2	UK	Contig	2016/2/10	
LSS24	SS891	GCA_900050795.1	2.11691	41.1	95312	2&1/2	UK	Contig	2016/2/10	
SS994	SS900	GCA_900054585.1	1.96115	41.3	233644	2&1/2	UK	Contig	2016/2/10	
LSS31	SS906	GCA_900063565.1	2.15427	41.4	159507	24	UK	Contig	2016/2/10	
SS986	SS915	GCA_900056565.1	2.16835	41	70328	12	UK	Scaffold	2016/2/10	
SS1052	SS916	GCA_900065395.1	2.22843	41	119393	3	UK	Contig	2016/2/10	
SS1017	SS923	GCA_900065005.1	2.07707	41.1	98069	2&1/2	UK	Contig	2016/2/10	
LSS79	SS926	GCA_900056515.1	2.20889	40.9	78683	15	UK	Contig	2016/2/10	
SS985	SS927	GCA_900061375.1	2.14017	41.1	85402	NCL6	UK	Scaffold	2016/2/10	

Strains	ReName	Assembly	Size (Mb)	GC%	N50	Molecular Serotypes	Country	Level	Release Date	Note
LOLA-SS006	SS929	GCA_900056825.1	2.16256	41	64520	4	UK	Contig	2016/2/10	
LSS49	SS930	GCA_900055105.1	2.23507	41.2	147612	NCL20	UK	Contig	2016/2/10	
LSS54	SS933	GCA_900053125.1	2.40191	41.1	44846	NCL10	UK	Contig	2016/2/10	
LOLA-SS011	SS941	GCA_900054535.1	2.04387	41.1	305870	1&14	UK	Contig	2016/2/10	
LSS64	SS953	GCA_900050805.1	2.3417	41.3	32447	21	UK	Contig	2016/2/10	
SS1056	SS967	GCA_900051945.1	2.06569	41.4	170239	8	UK	Scaffold	2016/2/10	
LSS20	SS969	GCA_900065385.1	2.26341	40.8	123207	4	UK	Contig	2016/2/10	
SS1061	SS972	GCA_900051275.1	2.10233	41.1	138030	8	UK	Contig	2016/2/10	
LS9N	SS828	GCA_900143575.1	2.56164	41.4	2561645	31	UK	Complete Genome	2016/12/9	
NCTC10234	SS1053	GCA_900475585.1	1.98438	41.4	1984375	2&1/2	UK	Complete Genome	2018/6/17	
ISU2912	SS212	GCA_001293335.1	2.61962	41.3	24258	NCL1	USA	Contig	2015/9/17	
ISU2660	SS322	GCA_001293355.1	2.10208	41.2	39581	2&1/2	USA	Contig	2015/9/17	
ISU2414	SS460	GCA_001293235.1	2.07523	41.3	35355	2&1/2	USA	Contig	2015/9/17	
ISU2714	SS599	GCA_001293295.1	2.0562	41.2	45174	2&1/2	USA	Contig	2015/9/17	
ISU2514	SS608	GCA_001293255.1	2.10236	41.3	25753	2&1/2	USA	Contig	2015/9/17	
ISU2614	SS623	GCA_001293215.1	2.11254	41.3	25453	not hit	USA	Contig	2015/9/17	
ISU1606	SS746	GCA_001293245.1	2.05843	41.1	58225	2&1/2	USA	Contig	2015/9/17	
ISU2812	SS866	GCA_001293305.1	2.25935	41.5	15144	not hit	USA	Contig	2015/9/17	
BM407	SS715	GCA_000026745.1	2.17081	41.05	2146229	2&1/2	Viet Nam	Complete Genome	2009/7/7	

Strains	ReName	Assembly	Size (Mb)	GC%	N50	Molecular Serotypes	Country	Level	Release Date	Note
S6O	SS012	GCA_000942415.1	2.0464	41.2	170384	2&1/2	Viet Nam	Scaffold	2015/2/14	
S7P	SS017	GCA_000938375.1	2.06999	41.1	170354	2&1/2	Viet Nam	Scaffold	2015/2/14	
V22C	SS022	GCA_000947335.1	2.1322	41.1	170360	2&1/2	Viet Nam	Scaffold	2015/2/14	
F4V	SS024	GCA_000946535.1	2.08348	41.2	170386	2&1/2	Viet Nam	Scaffold	2015/2/14	
E58D	SS029	GCA_000876835.1	2.04244	41.2	170389	2&1/2	Viet Nam	Scaffold	2015/2/14	
B11G	SS031	GCA_000937255.1	1.9813	41.2	170362	2&1/2	Viet Nam	Scaffold	2015/2/14	
F7L	SS034	GCA_000939315.1	2.04772	41.2	170367	2&1/2	Viet Nam	Scaffold	2015/2/14	
B23J	SS035	GCA_000937675.1	2.05971	41.1	170400	2&1/2	Viet Nam	Scaffold	2015/2/14	
F1N	SS036	GCA_000941915.1	2.06255	41.1	168771	2&1/2	Viet Nam	Scaffold	2015/2/14	
type strain: B40	SS038	GCA_000876855.1	2.10203	41.1	207288	2&1/2	Viet Nam	Scaffold	2015/2/14	
B48N	SS045	GCA_000877195.1	2.10762	41	170360	2&1/2	Viet Nam	Scaffold	2015/2/14	
S2X	SS047	GCA_000876995.1	2.01666	41.2	170288	2&1/2	Viet Nam	Scaffold	2015/2/14	
B23U	SS048	GCA_000940155.1	2.05766	41.1	170361	2&1/2	Viet Nam	Scaffold	2015/2/14	
S8Z	SS053	GCA_000941415.1	2.06414	41.1	169463	2&1/2	Viet Nam	Scaffold	2015/2/14	
type strain: E24	SS057	GCA_000942435.1	2.03628	41.2	170361	2&1/2	Viet Nam	Scaffold	2015/2/14	
F9O	SS062	GCA_000947395.1	2.06832	41.2	168466	2&1/2	Viet Nam	Scaffold	2015/2/14	
B38A	SS077	GCA_000944655.1	2.05285	41.1	92677	2&1/2	Viet Nam	Scaffold	2015/2/14	
type strain: S7	SS080	GCA_000947475.1	2.05532	41.1	170182	2&1/2	Viet Nam	Scaffold	2015/2/14	
B17H	SS082	GCA_000877135.1	1.96933	41.3	139693	2&1/2	Viet Nam	Scaffold	2015/2/14	

Strains	ReName	Assembly	Size (Mb)	GC%	N50	Molecular Serotypes	Country	Level	Release Date	Note
B23M	SS084	GCA_000938935.1	2.05344	41.1	170354	2&1/2	Viet Nam	Scaffold	2015/2/14	
V36H	SS086	GCA_000938635.1	2.0645	41.1	169463	2&1/2	Viet Nam	Scaffold	2015/2/14	
V30G	SS100	GCA_000936555.1	2.06451	41.1	169462	2&1/2	Viet Nam	Scaffold	2015/2/14	
F36U	SS102	GCA_000941355.1	2.07275	41.2	170389	2&1/2	Viet Nam	Scaffold	2015/2/14	
S8Y	SS111	GCA_000936935.1	2.08716	41.1	170374	2&1/2	Viet Nam	Scaffold	2015/2/14	
V30R	SS112	GCA_000943835.1	2.05909	41.1	169450	2&1/2	Viet Nam	Scaffold	2015/2/14	
F41Z	SS113	GCA_000940375.1	2.042	41.1	170106	2&1/2	Viet Nam	Scaffold	2015/2/14	
F50I	SS114	GCA_000941135.1	2.04666	41.1	130496	2&1/2	Viet Nam	Scaffold	2015/2/14	
B27R	SS119	GCA_000943875.1	2.09065	41.2	151039	2&1/2	Viet Nam	Scaffold	2015/2/14	
B10F	SS121	GCA_000947635.1	2.09236	41.1	170366	2&1/2	Viet Nam	Scaffold	2015/2/14	
V32E	SS126	GCA_000876955.1	2.15228	41	94866	2&1/2	Viet Nam	Scaffold	2015/2/14	
B48Y	SS127	GCA_000944055.1	2.05367	41.2	170386	2&1/2	Viet Nam	Scaffold	2015/2/14	
B46W	SS131	GCA_000940595.1	2.05891	41.1	130849	2&1/2	Viet Nam	Scaffold	2015/2/14	
B27V	SS138	GCA_000937015.1	1.99195	41.2	170093	2&1/2	Viet Nam	Scaffold	2015/2/14	
E56B	SS141	GCA_000939795.1	2.04784	41.2	170381	2&1/2	Viet Nam	Scaffold	2015/2/14	
F57E	SS149	GCA_000947575.1	2.04697	41.1	130578	2&1/2	Viet Nam	Scaffold	2015/2/14	
S7S	SS163	GCA_000945475.1	2.10034	41.1	131447	2&1/2	Viet Nam	Scaffold	2015/2/14	
S3Z	SS165	GCA_000947795.1	2.03685	41.2	170371	2&1/2	Viet Nam	Scaffold	2015/2/14	
S2Q	SS169	GCA_000941175.1	2.07688	41.2	170360	2&1/2	Viet Nam	Scaffold	2015/2/14	

Strains	ReName	Assembly	Size (Mb)	GC%	N50	Molecular Serotypes	Country	Level	Release Date	Note
B33Z	SS184	GCA_000876975.1	2.0483	41.2	169890	2&1/2	Viet Nam	Scaffold	2015/2/14	
B24W	SS189	GCA_000876695.1	2.06097	41.1	170363	2&1/2	Viet Nam	Scaffold	2015/2/14	
S2Y	SS197	GCA_000942975.1	2.08384	41.2	170265	2&1/2	Viet Nam	Scaffold	2015/2/14	
E00F	SS198	GCA_000945295.1	2.08875	41.2	170263	2&1/2	Viet Nam	Scaffold	2015/2/14	
V38Z	SS225	GCA_000939735.1	2.04179	41.2	130856	2&1/2	Viet Nam	Scaffold	2015/2/14	
S2U	SS228	GCA_000937755.1	2.06011	41.1	170361	2&1/2	Viet Nam	Scaffold	2015/2/14	
S1M	SS235	GCA_000936875.1	2.05398	41.2	170360	2&1/2	Viet Nam	Scaffold	2015/2/14	
E62H	SS243	GCA_000942715.1	2.05548	41.2	170334	2&1/2	Viet Nam	Scaffold	2015/2/14	
E05E	SS253	GCA_000946795.1	2.07359	41.2	208007	2&1/2	Viet Nam	Scaffold	2015/2/14	
F20Q	SS257	GCA_000944635.1	2.20333	40.9	125994	2&1/2	Viet Nam	Scaffold	2015/2/14	
S3C	SS259	GCA_000940055.1	2.06416	41.1	170365	2&1/2	Viet Nam	Scaffold	2015/2/14	
S9J	SS266	GCA_000938755.1	2.05204	41.1	170361	2&1/2	Viet Nam	Scaffold	2015/2/14	
S3G	SS271	GCA_000939895.1	2.05857	41.1	170174	2&1/2	Viet Nam	Scaffold	2015/2/14	
E04J	SS273	GCA_000942535.1	2.09603	41.1	170205	2&1/2	Viet Nam	Scaffold	2015/2/14	
type strain: B21	SS290	GCA_000940075.1	2.22856	40.9	119698	2&1/2	Viet Nam	Scaffold	2015/2/14	
V27D	SS292	GCA_000945815.1	2.0642	41.1	169657	2&1/2	Viet Nam	Scaffold	2015/2/14	
B36G	SS294	GCA_000941475.1	2.05892	41.1	130801	2&1/2	Viet Nam	Scaffold	2015/2/14	
0Z-B	SS295	GCA_000938815.1	2.05341	41.2	170261	2&1/2	Viet Nam	Scaffold	2015/2/14	
V36U	SS302	GCA_000944775.1	2.05335	41.2	145876	2&1/2	Viet Nam	Scaffold	2015/2/14	

Strains	ReName	Assembly	Size (Mb)	GC%	N50	Molecular Serotypes	Country	Level	Release Date	Note
F53J	SS304	GCA_000943195.1	2.08311	41.1	123959	2&1/2	Viet Nam	Scaffold	2015/2/14	
B21Q	SS305	GCA_000944815.1	2.21062	41.1	116747	2&1/2	Viet Nam	Scaffold	2015/2/14	
E57C	SS306	GCA_000944975.1	2.0964	41.1	170361	2&1/2	Viet Nam	Scaffold	2015/2/14	
V37W	SS307	GCA_000947595.1	2.06563	41.1	170358	2&1/2	Viet Nam	Scaffold	2015/2/14	
B41M	SS308	GCA_000944315.1	2.05422	41.1	170359	2&1/2	Viet Nam	Scaffold	2015/2/14	
S8U	SS309	GCA_000936595.1	2.04913	41.2	170264	2&1/2	Viet Nam	Scaffold	2015/2/14	
E11O	SS315	GCA_000936535.1	2.03933	41.2	130502	2&1/2	Viet Nam	Scaffold	2015/2/14	
S3D	SS317	GCA_000937995.1	2.04175	41.2	170362	2&1/2	Viet Nam	Scaffold	2015/2/14	
E14M	SS335	GCA_000944915.1	2.03241	41.2	170385	2&1/2	Viet Nam	Scaffold	2015/2/14	
F49X	SS341	GCA_000946015.1	2.04234	41.1	170105	2&1/2	Viet Nam	Scaffold	2015/2/14	
E55E	SS344	GCA_000947615.1	2.03879	41.2	170373	2&1/2	Viet Nam	Scaffold	2015/2/14	
B44O	SS359	GCA_000877115.1	2.04803	41.2	170297	2&1/2	Viet Nam	Scaffold	2015/2/14	
F19J	SS362	GCA_000944675.1	2.03645	41.2	170384	2&1/2	Viet Nam	Scaffold	2015/2/14	
B28Y	SS365	GCA_000942735.1	2.0338	41.1	170366	2&1/2	Viet Nam	Scaffold	2015/2/14	
B41Q	SS371	GCA_000942675.1	2.05718	41.1	170259	2&1/2	Viet Nam	Scaffold	2015/2/14	
V31J	SS373	GCA_000943975.1	2.0605	41.1	170360	2&1/2	Viet Nam	Scaffold	2015/2/14	
S3E	SS381	GCA_000947835.1	2.04814	41.2	170358	2&1/2	Viet Nam	Scaffold	2015/2/14	
E00C	SS383	GCA_000946875.1	2.05744	41.1	130801	2&1/2	Viet Nam	Scaffold	2015/2/14	
B22X	SS390	GCA_000938615.1	2.10794	41.2	170390	2&1/2	Viet Nam	Scaffold	2015/2/14	

Strains	ReName	Assembly	Size (Mb)	GC%	N50	Molecular Serotypes	Country	Level	Release Date	Note
CU2A	SS394	GCA_000942295.1	2.0424	41.2	131449	2&1/2	Viet Nam	Scaffold	2015/2/14	
F25R	SS397	GCA_000942555.1	2.05167	41.1	130067	2&1/2	Viet Nam	Scaffold	2015/2/14	
S2V	SS399	GCA_000941595.1	2.04743	41.2	170378	2&1/2	Viet Nam	Scaffold	2015/2/14	
E21U	SS403	GCA_000942355.1	2.0528	41.2	170279	2&1/2	Viet Nam	Scaffold	2015/2/14	
B44R	SS405	GCA_000942455.1	2.04794	41.2	168760	2&1/2	Viet Nam	Scaffold	2015/2/14	
V39Q	SS412	GCA_000937195.1	2.03796	41.2	131449	2&1/2	Viet Nam	Scaffold	2015/2/14	
S6L	SS415	GCA_000941955.1	2.06469	41.1	170353	2&1/2	Viet Nam	Scaffold	2015/2/14	
E23S	SS419	GCA_000939855.1	2.08453	41.2	170376	2&1/2	Viet Nam	Scaffold	2015/2/14	
S1N	SS440	GCA_000942475.1	2.05944	41.1	170392	2&1/2	Viet Nam	Scaffold	2015/2/14	
E01D	SS441	GCA_000943895.1	2.05923	41.1	130856	2&1/2	Viet Nam	Scaffold	2015/2/14	
F43B	SS443	GCA_000945575.1	2.05139	41.2	52897	2&1/2	Viet Nam	Scaffold	2015/2/14	
F2S	SS461	GCA_000939875.1	2.08415	41.2	170384	2&1/2	Viet Nam	Scaffold	2015/2/14	
V35S	SS464	GCA_000944075.1	2.05898	41.1	169448	2&1/2	Viet Nam	Scaffold	2015/2/14	
F21O	SS476	GCA_000946075.1	2.1136	41	170264	2&1/2	Viet Nam	Scaffold	2015/2/14	
E01I	SS477	GCA_000936975.1	2.03674	41.2	170368	2&1/2	Viet Nam	Scaffold	2015/2/14	
E21R	SS485	GCA_000877215.1	2.12333	41.1	92716	2&1/2	Viet Nam	Scaffold	2015/2/14	
E63F	SS489	GCA_000940095.1	2.05197	41.2	170152	2&1/2	Viet Nam	Scaffold	2015/2/14	
F5F	SS490	GCA_000940715.1	2.08158	41.1	123953	2&1/2	Viet Nam	Scaffold	2015/2/14	
F5K	SS492	GCA_000937595.1	2.06459	41.1	170399	2&1/2	Viet Nam	Scaffold	2015/2/14	

Strains	ReName	Assembly	Size (Mb)	GC%	N50	Molecular Serotypes	Country	Level	Release Date	Note
S8W	SS497	GCA_000941795.1	2.12928	41.1	131049	2&1/2	Viet Nam	Scaffold	2015/2/14	
E36X	SS502	GCA_000946515.1	2.03501	41.2	170391	2&1/2	Viet Nam	Scaffold	2015/2/14	
E11Q	SS509	GCA_000946395.1	2.04226	41.2	135916	1&14	Viet Nam	Scaffold	2015/2/14	
E03B	SS526	GCA_000946995.1	2.05964	41.1	170359	2&1/2	Viet Nam	Scaffold	2015/2/14	
0Z-D	SS529	GCA_000938475.1	2.05333	41.2	170362	2&1/2	Viet Nam	Scaffold	2015/2/14	
S7Q	SS536	GCA_000937375.1	2.04697	41.2	170265	2&1/2	Viet Nam	Scaffold	2015/2/14	
B28P	SS540	GCA_000940635.1	2.05039	41.2	169951	2&1/2	Viet Nam	Scaffold	2015/2/14	
B29K	SS549	GCA_000876775.1	1.99071	41.2	130638	2&1/2	Viet Nam	Scaffold	2015/2/14	
B39D	SS550	GCA_000941675.1	2.03779	41.2	170265	2&1/2	Viet Nam	Scaffold	2015/2/14	
F1L	SS555	GCA_000945335.1	2.05818	41.1	156400	2&1/2	Viet Nam	Scaffold	2015/2/14	
B37H	SS560	GCA_000943935.1	2.05921	41.1	127823	2&1/2	Viet Nam	Scaffold	2015/2/14	
E60I	SS561	GCA_000938435.1	2.03838	41.2	131449	2&1/2	Viet Nam	Scaffold	2015/2/14	
E30Z	SS562	GCA_000944035.1	2.07055	41.2	170389	2&1/2	Viet Nam	Scaffold	2015/2/14	
F17K	SS564	GCA_000937155.1	2.09881	41.1	130849	2&1/2	Viet Nam	Scaffold	2015/2/14	
S2W	SS568	GCA_000940015.1	2.09538	41.1	137040	2&1/2	Viet Nam	Scaffold	2015/2/14	
V34P	SS569	GCA_000936835.1	2.07828	41.1	169582	2&1/2	Viet Nam	Scaffold	2015/2/14	
E39A	SS575	GCA_000941255.1	2.14161	41	130863	2&1/2	Viet Nam	Scaffold	2015/2/14	
E10N	SS578	GCA_000945275.1	2.06498	41.1	170379	2&1/2	Viet Nam	Scaffold	2015/2/14	
E17P	SS589	GCA_000937055.1	2.08202	41.1	170361	2&1/2	Viet Nam	Scaffold	2015/2/14	

Strains	ReName	Assembly	Size (Mb)	GC%	N50	Molecular Serotypes	Country	Level	Release Date	Note
F2P	SS601	GCA_000939055.1	2.0702	41.2	170362	2&1/2	Viet Nam	Scaffold	2015/2/14	
V36M	SS607	GCA_000938775.1	2.05885	41.1	169459	2&1/2	Viet Nam	Scaffold	2015/2/14	
S1L	SS611	GCA_000938055.1	2.04779	41.2	170154	2&1/2	Viet Nam	Scaffold	2015/2/14	
V38Y	SS615	GCA_000945635.1	2.08293	41.2	170409	2&1/2	Viet Nam	Scaffold	2015/2/14	
V38X	SS621	GCA_000947515.1	2.05946	41.1	169447	2&1/2	Viet Nam	Scaffold	2015/2/14	
S6M	SS622	GCA_000943235.1	2.07039	41.3	130849	2&1/2	Viet Nam	Scaffold	2015/2/14	
B18E	SS624	GCA_000876935.1	2.10126	41	170154	2&1/2	Viet Nam	Scaffold	2015/2/14	
E21V	SS625	GCA_000945375.1	2.06994	41.2	170355	2&1/2	Viet Nam	Scaffold	2015/2/14	
S2P	SS628	GCA_000945055.1	2.04813	41.2	170386	2&1/2	Viet Nam	Scaffold	2015/2/14	
V34L	SS632	GCA_000940475.1	2.05865	41.1	169618	2&1/2	Viet Nam	Scaffold	2015/2/14	
S3A	SS638	GCA_000876875.1	2.05946	41.1	170355	2&1/2	Viet Nam	Scaffold	2015/2/14	
S4K	SS640	GCA_000941775.1	2.07877	41.2	170282	2&1/2	Viet Nam	Scaffold	2015/2/14	
E04H	SS643	GCA_000936995.1	2.06456	41.1	170391	2&1/2	Viet Nam	Scaffold	2015/2/14	
B45V	SS649	GCA_000944555.1	2.04208	41.2	170354	2&1/2	Viet Nam	Scaffold	2015/2/14	
B41U	SS666	GCA_000943915.1	2.05776	41.2	170361	2&1/2	Viet Nam	Scaffold	2015/2/14	
S4H	SS674	GCA_000939995.1	2.10896	41	142565	2&1/2	Viet Nam	Scaffold	2015/2/14	
B24O	SS676	GCA_000945855.1	2.1155	41	170372	2&1/2	Viet Nam	Scaffold	2015/2/14	
S4I	SS679	GCA_000947735.1	2.07658	41.1	170438	2&1/2	Viet Nam	Scaffold	2015/2/14	
F51C	SS690	GCA_000942615.1	2.0537	41.1	170398	2&1/2	Viet Nam	Scaffold	2015/2/14	

Strains	ReName	Assembly	Size (Mb)	GC%	N50	Molecular Serotypes	Country	Level	Release Date	Note
E03G	SS694	GCA_000944575.1	2.0484	41.2	170361	2&1/2	Viet Nam	Scaffold	2015/2/14	
B35B	SS696	GCA_000876715.1	2.04722	41.2	170387	2&1/2	Viet Nam	Scaffold	2015/2/14	
type strain: F38	SS698	GCA_000942115.1	2.07718	41.2	170387	2&1/2	Viet Nam	Scaffold	2015/2/14	
S1J	SS701	GCA_000876735.1	2.08566	41.2	170267	2&1/2	Viet Nam	Scaffold	2015/2/14	
0Z-C	SS702	GCA_000942575.1	2.05331	41.2	170362	2&1/2	Viet Nam	Scaffold	2015/2/14	
B18I	SS714	GCA_000937235.1	2.05725	41.1	130865	2&1/2	Viet Nam	Scaffold	2015/2/14	
B21L	SS718	GCA_000945255.1	2.03723	41.2	170206	2&1/2	Viet Nam	Scaffold	2015/2/14	
B36K	SS723	GCA_000941695.1	2.09434	41.1	170361	2&1/2	Viet Nam	Scaffold	2015/2/14	
B38I	SS731	GCA_000944415.1	2.04024	41.2	130503	2&1/2	Viet Nam	Scaffold	2015/2/14	
S2R	SS732	GCA_000943275.1	2.04142	41.2	130494	2&1/2	Viet Nam	Scaffold	2015/2/14	
S7R	SS734	GCA_000946575.1	2.06291	41.1	170231	2&1/2	Viet Nam	Scaffold	2015/2/14	
E08L	SS740	GCA_000942895.1	2.04848	41.2	170354	2&1/2	Viet Nam	Scaffold	2015/2/14	
B26N	SS742	GCA_000937635.1	1.98272	41.2	170374	2&1/2	Viet Nam	Scaffold	2015/2/14	
E66G	SS751	GCA_000946115.1	2.09639	41.1	130849	2&1/2	Viet Nam	Scaffold	2015/2/14	
S6N	SS752	GCA_000876915.1	2.04354	41.3	10592	2&1/2	Viet Nam	Scaffold	2015/2/14	
V38O	SS753	GCA_000876815.1	2.08241	41.2	170150	2&1/2	Viet Nam	Scaffold	2015/2/14	
V20B	SS760	GCA_000939935.1	2.0582	41.1	169413	2&1/2	Viet Nam	Scaffold	2015/2/14	
F5D	SS761	GCA_000946675.1	2.06033	41.1	168775	2&1/2	Viet Nam	Scaffold	2015/2/14	
F14M	SS762	GCA_000941755.1	2.09994	41	131440	2&1/2	Viet Nam	Scaffold	2015/2/14	

Strains	ReName	Assembly	Size (Mb)	GC%	N50	Molecular Serotypes	Country	Level	Release Date	Note
CU2Z	SS767	GCA_000945095.1	2.04291	41.2	131463	2&1/2	Viet Nam	Scaffold	2015/2/14	
0Z-A	SS775	GCA_000946355.1	2.05991	41.1	170376	2&1/2	Viet Nam	Scaffold	2015/2/14	
V39F	SS785	GCA_000877095.1	2.06487	41.1	169568	2&1/2	Viet Nam	Scaffold	2015/2/14	
B35F	SS786	GCA_000945395.1	2.00526	41.2	170365	2&1/2	Viet Nam	Scaffold	2015/2/14	
E00K	SS793	GCA_000947255.1	2.07726	41.2	170367	2&1/2	Viet Nam	Scaffold	2015/2/14	
S3F	SS796	GCA_000946555.1	2.06301	41.1	170258	2&1/2	Viet Nam	Scaffold	2015/2/14	
S1K	SS813	GCA_000947095.1	2.04226	41.2	170360	2&1/2	Viet Nam	Scaffold	2015/2/14	
B42S	SS814	GCA_000877235.1	2.08881	41.2	170357	2&1/2	Viet Nam	Scaffold	2015/2/14	
S4J	SS823	GCA_000943335.1	2.05784	41.1	170386	2&1/2	Viet Nam	Scaffold	2015/2/14	
S2S	SS825	GCA_000942175.1	2.08811	41.2	170293	2&1/2	Viet Nam	Scaffold	2015/2/14	
B46P	SS826	GCA_000945435.1	2.0646	41.1	170359	2&1/2	Viet Nam	Scaffold	2015/2/14	
B31C	SS827	GCA_000943995.1	2.03837	41.2	131449	2&1/2	Viet Nam	Scaffold	2015/2/14	
B41X	SS836	GCA_000942195.1	2.04732	41.2	168838	2&1/2	Viet Nam	Scaffold	2015/2/14	
E34W	SS843	GCA_000942235.1	2.04609	41.2	135941	1&14	Viet Nam	Scaffold	2015/2/14	
V37N	SS846	GCA_000940195.1	2.0599	41.1	169482	2&1/2	Viet Nam	Scaffold	2015/2/14	
type strain: S2	SS847	GCA_000944935.1	2.05361	41.1	170354	2&1/2	Viet Nam	Scaffold	2015/2/14	
B21S	SS862	GCA_000942755.1	2.19563	41	131016	2&1/2	Viet Nam	Scaffold	2015/2/14	
S8X	SS868	GCA_000941735.1	2.08429	41.2	170358	2&1/2	Viet Nam	Scaffold	2015/2/14	
S8V	SS874	GCA_000946955.1	2.05039	41.2	171056	2&1/2	Viet Nam	Scaffold	2015/2/14	

Strains	ReName	Assembly	Size (Mb)	GC%	N50	Molecular Serotypes	Country	Level	Release Date	Note
B32J	SS877	GCA_000944515.1	2.01615	41.2	170265	2&1/2	Viet Nam	Scaffold	2015/2/14	
S1O	SS886	GCA_000947755.1	2.03677	41.2	170360	2&1/2	Viet Nam	Scaffold	2015/2/14	
V36V	SS890	GCA_000876895.1	2.10967	41.2	170274	2&1/2	Viet Nam	Scaffold	2015/2/14	
F48Y	SS905	GCA_000944875.1	2.0419	41.1	140742	2&1/2	Viet Nam	Scaffold	2015/2/14	
F48A	SS907	GCA_000940395.1	2.03774	41.2	131440	2&1/2	Viet Nam	Scaffold	2015/2/14	
S3B	SS909	GCA_000938655.1	2.17675	40.9	130863	2&1/2	Viet Nam	Scaffold	2015/2/14	
F7N	SS919	GCA_000944135.1	2.05287	41.2	170409	2&1/2	Viet Nam	Scaffold	2015/2/14	
E30Y	SS921	GCA_000945735.1	2.04845	41.2	170362	2&1/2	Viet Nam	Scaffold	2015/2/14	
V31I	SS932	GCA_000947675.1	2.03614	41.2	170360	2&1/2	Viet Nam	Scaffold	2015/2/14	
B34E	SS937	GCA_000938595.1	2.05255	41.2	170368	2&1/2	Viet Nam	Scaffold	2015/2/14	
V33K	SS939	GCA_000940495.1	2.07001	41.1	170362	2&1/2	Viet Nam	Scaffold	2015/2/14	
F7M	SS943	GCA_000936895.1	2.0599	41.1	223370	2&1/2	Viet Nam	Scaffold	2015/2/14	
type strain: V30	SS946	GCA_000877175.1	2.05958	41.1	169450	2&1/2	Viet Nam	Scaffold	2015/2/14	
F57G	SS956	GCA_000946035.1	2.04633	41.1	130495	2&1/2	Viet Nam	Scaffold	2015/2/14	
F55H	SS958	GCA_000942495.1	2.04551	41.1	130592	2&1/2	Viet Nam	Scaffold	2015/2/14	
F45W	SS959	GCA_000947075.1	2.04167	41.1	170115	2&1/2	Viet Nam	Scaffold	2015/2/14	
B33L	SS974	GCA_000939115.1	2.0593	41.1	130787	2&1/2	Viet Nam	Scaffold	2015/2/14	
FX543_1	SS001	GCA_900062305.1	2.08194	41.1	176249	2&1/2	Viet Nam	Contig	2016/2/10	
VS3865	SS004	GCA_900058065.1	2.11029	41.2	228986	2&1/2	Viet Nam	Contig	2016/2/10	

Strains	ReName	Assembly	Size (Mb)	GC%	N50	Molecular Serotypes	Country	Level	Release Date	Note
BM321C	SS005	GCA_900053795.1	2.03855	41.2	171186	2&1/2	Viet Nam	Contig	2016/2/10	
BM362a	SS009	GCA_900059175.1	2.01598	41.2	222114	2&1/2	Viet Nam	Contig	2016/2/10	
VS3888	SS011	GCA_900061075.1	2.04123	41.2	170309	2&1/2	Viet Nam	Contig	2016/2/10	
VS3709	SS019	GCA_900055895.1	2.05752	41.1	170505	2&1/2	Viet Nam	Contig	2016/2/10	
BM213a	SS020	GCA_900049655.1	2.05419	41.1	170454	2&1/2	Viet Nam	Contig	2016/2/10	
EN508	SS030	GCA_900059925.1	2.04219	41.2	170436	2&1/2	Viet Nam	Contig	2016/2/10	
FX478	SS033	GCA_900058315.1	2.03695	41.2	171994	2&1/2	Viet Nam	Contig	2016/2/10	
BM178a	SS041	GCA_900060965.1	2.11075	41.1	170509	2&1/2	Viet Nam	Contig	2016/2/10	
BM231a	SS052	GCA_900059465.1	2.0512	41.2	222587	2&1/2	Viet Nam	Contig	2016/2/10	
BM228C	SS055	GCA_900049125.1	2.04924	41.1	236163	2&1/2	Viet Nam	Contig	2016/2/10	
BM465	SS058	GCA_900059785.1	2.03693	41.2	170757	2&1/2	Viet Nam	Contig	2016/2/10	
EN660	SS071	GCA_900058505.1	2.03771	41.2	171220	2&1/2	Viet Nam	Contig	2016/2/10	
BM315C	SS074	GCA_900049145.1	2.04907	41.2	172158	2&1/2	Viet Nam	Contig	2016/2/10	
VS3309	SS075	GCA_900058775.1	2.06204	41.1	169767	2&1/2	Viet Nam	Contig	2016/2/10	
FX483_3	SS083	GCA_900058075.1	2.04677	41.1	58264	2&1/2	Viet Nam	Contig	2016/2/10	
SS26	SS090	GCA_900058285.1	2.05396	41.2	172156	2&1/2	Viet Nam	Contig	2016/2/10	
EN633	SS094	GCA_900054305.1	2.05187	41.2	223106	2&1/2	Viet Nam	Contig	2016/2/10	
SS25	SS096	GCA_900056585.1	2.05339	41.1	171832	2&1/2	Viet Nam	Contig	2016/2/10	
BM460	SS106	GCA_900059915.1	2.09521	41.1	208209	2&1/2	Viet Nam	Contig	2016/2/10	

Strains	ReName	Assembly	Size (Mb)	GC%	N50	Molecular Serotypes	Country	Level	Release Date	Note
VS2979	SS109	GCA_900051995.1	2.06306	41.1	169910	2&1/2	Viet Nam	Contig	2016/2/10	
SS19	SS120	GCA_900065095.1	2.04178	41.2	171436	2&1/2	Viet Nam	Contig	2016/2/10	
01ZN-ST222	SS132	GCA_900061405.1	2.06876	41.1	239376	2&1/2	Viet Nam	Contig	2016/2/10	
EN010	SS142	GCA_900057075.1	2.05684	41.1	222206	2&1/2	Viet Nam	Contig	2016/2/10	
BM418a	SS143	GCA_900063625.1	2.11208	41	187741	2&1/2	Viet Nam	Contig	2016/2/10	
BM424a	SS144	GCA_900061025.1	2.04736	41.2	171234	2&1/2	Viet Nam	Contig	2016/2/10	
FX59_4	SS146	GCA_900049675.1	2.05885	41.1	233662	2&1/2	Viet Nam	Contig	2016/2/10	
SS78	SS147	GCA_900063295.1	2.09748	41.1	131486	2&1/2	Viet Nam	Contig	2016/2/10	
BM466	SS155	GCA_900053185.1	2.05852	41.1	159727	2&1/2	Viet Nam	Contig	2016/2/10	
BM224C	SS157	GCA_900050445.1	2.05881	41.1	170426	2&1/2	Viet Nam	Contig	2016/2/10	
EN044	SS158	GCA_900063285.1	2.10188	41.1	222199	2&1/2	Viet Nam	Contig	2016/2/10	
EN124	SS161	GCA_900061395.1	2.03757	41.2	170438	2&1/2	Viet Nam	Contig	2016/2/10	
BM441a	SS175	GCA_900050035.1	2.0666	41.1	200557	2&1/2	Viet Nam	Contig	2016/2/10	
EN068	SS181	GCA_900058755.1	2.03745	41.1	170458	2&1/2	Viet Nam	Contig	2016/2/10	
BM257a	SS185	GCA_900059485.1	1.99228	41.2	236281	2&1/2	Viet Nam	Contig	2016/2/10	
FX90_2	SS194	GCA_900049175.1	2.06704	41.2	236339	2&1/2	Viet Nam	Contig	2016/2/10	
VS3569	SS203	GCA_900055515.1	2.05728	41.1	171112	2&1/2	Viet Nam	Contig	2016/2/10	
EN171	SS205	GCA_900065795.1	2.03863	41.2	163524	2&1/2	Viet Nam	Contig	2016/2/10	
SS17	SS213	GCA_900060355.1	2.05887	41.1	170460	2&1/2	Viet Nam	Contig	2016/2/10	

Strains	ReName	Assembly	Size (Mb)	GC%	N50	Molecular Serotypes	Country	Level	Release Date	Note
SS47	SS214	GCA_900058295.1	2.07361	41.2	171444	2&1/2	Viet Nam	Contig	2016/2/10	
FX211	SS218	GCA_900065825.1	2.10635	41	222199	2&1/2	Viet Nam	Contig	2016/2/10	
SS44	SS220	GCA_900050885.1	2.06535	41.1	170426	2&1/2	Viet Nam	Contig	2016/2/10	
SS40	SS223	GCA_900052855.1	2.10839	41	142612	2&1/2	Viet Nam	Contig	2016/2/10	
EN271	SS226	GCA_900058045.1	2.05358	41.2	326348	2&1/2	Viet Nam	Contig	2016/2/10	
SS27	SS234	GCA_900059525.1	2.09573	41.1	170702	2&1/2	Viet Nam	Contig	2016/2/10	
EN314	SS236	GCA_900050875.1	2.04674	41.2	136066	1&14	Viet Nam	Contig	2016/2/10	
FX50_2	SS238	GCA_900049665.1	2.05821	41.1	223647	2&1/2	Viet Nam	Contig	2016/2/10	
BM471	SS239	GCA_900053195.1	2.04821	41.2	171914	2&1/2	Viet Nam	Contig	2016/2/10	
BM452C	SS240	GCA_900054855.1	2.08272	41.2	170427	2&1/2	Viet Nam	Contig	2016/2/10	
VS3517	SS247	GCA_900055135.1	2.06022	41.1	170451	2&1/2	Viet Nam	Contig	2016/2/10	
SS66	SS248	GCA_900059535.1	2.0376	41.2	13306	2&1/2	Viet Nam	Scaffold	2016/2/10	
EN370	SS256	GCA_900051985.1	2.07129	41.2	170461	2&1/2	Viet Nam	Contig	2016/2/10	
SS97	SS264	GCA_900065255.1	2.05208	41.1	222754	2&1/2	Viet Nam	Contig	2016/2/10	
BM264a	SS269	GCA_900065415.1	2.05481	41.1	222292	2&1/2	Viet Nam	Contig	2016/2/10	
BM346B	SS274	GCA_900057065.1	2.0635	41.1	222198	2&1/2	Viet Nam	Contig	2016/2/10	
FX540_1	SS279	GCA_900061435.1	2.04733	41.1	170744	2&1/2	Viet Nam	Contig	2016/2/10	
EN507	SS281	GCA_900057515.1	2.10257	41.1	234044	2&1/2	Viet Nam	Contig	2016/2/10	
SS36	SS282	GCA_900052845.1	2.04842	41.2	171312	2&1/2	Viet Nam	Contig	2016/2/10	

Strains	ReName	Assembly	Size (Mb)	GC%	N50	Molecular Serotypes	Country	Level	Release Date	Note
FX184_2	SS286	GCA_900049185.1	2.10436	41.1	171870	2&1/2	Viet Nam	Contig	2016/2/10	
VS3587	SS300	GCA_900055885.1	2.08337	41.2	170217	2&1/2	Viet Nam	Contig	2016/2/10	
BM197a	SS303	GCA_900065065.1	1.97423	41.3	198341	2&1/2	Viet Nam	Contig	2016/2/10	
VS3537	SS316	GCA_900057715.1	2.06955	41.1	171315	2&1/2	Viet Nam	Contig	2016/2/10	
SS24	SS326	GCA_900054315.1	2.05356	41.1	171268	2&1/2	Viet Nam	Contig	2016/2/10	
FX18	SS327	GCA_900054605.1	2.05794	41.1	236399	2&1/2	Viet Nam	Contig	2016/2/10	
SS73	SS330	GCA_900059205.1	2.06553	41.1	170709	2&1/2	Viet Nam	Contig	2016/2/10	
SS83	SS332	GCA_900052865.1	2.05348	41.1	222104	2&1/2	Viet Nam	Contig	2016/2/10	
EN191	SS337	GCA_900064035.1	2.04394	41.2	172156	1&14	Viet Nam	Contig	2016/2/10	
FX73_1	SS338	GCA_900065265.1	2.04693	41.2	170723	2&1/2	Viet Nam	Contig	2016/2/10	
SS81	SS340	GCA_900065245.1	2.05059	41.2	224930	2&1/2	Viet Nam	Contig	2016/2/10	
01ZN-TG183	SS343	GCA_900061415.1	2.04933	41.2	172153	2&1/2	Viet Nam	Contig	2016/2/10	
BM478	SS347	GCA_900055875.1	2.04759	41.2	234078	2&1/2	Viet Nam	Contig	2016/2/10	
SS28	SS352	GCA_900053205.1	2.01594	41.2	222111	2&1/2	Viet Nam	Contig	2016/2/10	
EN652	SS358	GCA_900063645.1	2.04657	41.2	238001	2&1/2	Viet Nam	Contig	2016/2/10	
BM345B	SS360	GCA_900057505.1	2.0048	41.2	235346	2&1/2	Viet Nam	Contig	2016/2/10	
BM251C	SS364	GCA_900059475.1	2.07061	41	234133	2&1/2	Viet Nam	Contig	2016/2/10	
EN0114	SS367	GCA_900058745.1	2.06117	41.1	170424	2&1/2	Viet Nam	Contig	2016/2/10	
01ZN-TG187	SS369	GCA_900061425.1	2.04889	41.2	234039	2&1/2	Viet Nam	Scaffold	2016/2/10	

Strains	ReName	Assembly	Size (Mb)	GC%	N50	Molecular Serotypes	Country	Level	Release Date	Note
01ZN-TG189	SS370	GCA_900056605.1	2.0534	41.2	170712	2&1/2	Viet Nam	Contig	2016/2/10	
BM357B	SS372	GCA_900059905.1	2.05952	41.1	223494	2&1/2	Viet Nam	Contig	2016/2/10	
SS99	SS374	GCA_900049155.1	2.07115	41.2	234029	2&1/2	Viet Nam	Contig	2016/2/10	
FX328_4	SS376	GCA_900050905.1	2.07172	41.2	170481	2&1/2	Viet Nam	Contig	2016/2/10	
SS23	SS380	GCA_900060675.1	2.08833	41.2	198521	2&1/2	Viet Nam	Contig	2016/2/10	
EN264	SS385	GCA_900050455.1	2.04317	41.2	170716	2&1/2	Viet Nam	Contig	2016/2/10	
BM366a	SS389	GCA_900053605.1	2.09313	41.1	172156	2&1/2	Viet Nam	Contig	2016/2/10	
FX74	SS391	GCA_900049365.1	2.06378	41.1	239706	2&1/2	Viet Nam	Contig	2016/2/10	
BM272C	SS395	GCA_900050025.1	2.10802	41.2	208396	2&1/2	Viet Nam	Contig	2016/2/10	
FX147_3	SS396	GCA_900050065.1	2.11032	41.1	184047	2&1/2	Viet Nam	Contig	2016/2/10	
BM334B	SS400	GCA_900054125.1	2.04805	41.2	234029	2&1/2	Viet Nam	Scaffold	2016/2/10	
VS3575	SS404	GCA_900055155.1	2.05876	41.1	223111	2&1/2	Viet Nam	Contig	2016/2/10	
SS79	SS406	GCA_900064695.1	2.06128	41.1	176276	2&1/2	Viet Nam	Contig	2016/2/10	
SS29	SS408	GCA_900065815.1	2.08802	41.2	170617	2&1/2	Viet Nam	Contig	2016/2/10	
FX51_2	SS411	GCA_900058785.1	2.08181	41.1	222759	2&1/2	Viet Nam	Contig	2016/2/10	
SS101	SS413	GCA_900053225.1	2.10254	41.1	204089	2&1/2	Viet Nam	Contig	2016/2/10	
FX535_2	SS417	GCA_900058325.1	2.04609	41.1	172905	2&1/2	Viet Nam	Contig	2016/2/10	
SS38	SS423	GCA_900057695.1	2.0583	41.1	171087	2&1/2	Viet Nam	Contig	2016/2/10	
SS15	SS424	GCA_900059195.1	2.04715	41.2	223402	2&1/2	Viet Nam	Contig	2016/2/10	

Strains	ReName	Assembly	Size (Mb)	GC%	N50	Molecular Serotypes	Country	Level	Release Date	Note
VS3886	SS430	GCA_900058525.1	2.0839	41.2	233779	2&1/2	Viet Nam	Contig	2016/2/10	
FX28_2	SS434	GCA_900064705.1	2.08333	41.2	170468	2&1/2	Viet Nam	Contig	2016/2/10	
BM329B	SS442	GCA_900058275.1	2.03838	41.2	223115	2&1/2	Viet Nam	Contig	2016/2/10	
SS32	SS450	GCA_900063655.1	2.1785	40.9	226113	2&1/2	Viet Nam	Contig	2016/2/10	
SS84	SS451	GCA_900056595.1	2.08914	41.2	239707	2&1/2	Viet Nam	Contig	2016/2/10	
EN251	SS455	GCA_900065805.1	2.12207	41.1	222194	2&1/2	Viet Nam	Contig	2016/2/10	
BM444a	SS456	GCA_900061035.1	2.04729	41.2	222104	2&1/2	Viet Nam	Contig	2016/2/10	
SS74	SS459	GCA_900059545.1	2.04598	41.2	170310	2&1/2	Viet Nam	Contig	2016/2/10	
EN020	SS462	GCA_900061045.1	2.09357	41.2	172058	2&1/2	Viet Nam	Contig	2016/2/10	
SS69	SS467	GCA_900057705.1	2.0528	41.2	170440	2&1/2	Viet Nam	Contig	2016/2/10	
BM278a	SS478	GCA_900054115.1	2.03314	41.1	222212	2&1/2	Viet Nam	Contig	2016/2/10	
EN399	SS479	GCA_900052835.1	2.13339	41	142374	2&1/2	Viet Nam	Contig	2016/2/10	
FX109	SS480	GCA_900050055.1	2.0368	41.2	170469	2&1/2	Viet Nam	Contig	2016/2/10	
SS87	SS481	GCA_900051285.1	2.06317	41.1	169902	2&1/2	Viet Nam	Contig	2016/2/10	
EN326	SS484	GCA_900052245.1	2.03426	41.2	170716	2&1/2	Viet Nam	Contig	2016/2/10	
FX22	SS495	GCA_900062115.1	2.07664	41.2	234049	2&1/2	Viet Nam	Contig	2016/2/10	
EN024	SS498	GCA_900061065.1	2.06987	41.1	170460	2&1/2	Viet Nam	Contig	2016/2/10	
SS61	SS510	GCA_900056875.1	2.06558	41.1	222818	2&1/2	Viet Nam	Contig	2016/2/10	
EN281	SS520	GCA_900058055.1	2.06945	41.2	234037	2&1/2	Viet Nam	Contig	2016/2/10	

Strains	ReName	Assembly	Size (Mb)	GC%	N50	Molecular Serotypes	Country	Level	Release Date	Note
VS3543	SS521	GCA_900055145.1	2.06272	41.1	223111	2&1/2	Viet Nam	Contig	2016/2/10	
VS2923	SS533	GCA_900049165.1	2.13199	41.1	234161	2&1/2	Viet Nam	Contig	2016/2/10	
BM253a	SS534	GCA_900065405.1	2.05659	41.1	233686	2&1/2	Viet Nam	Contig	2016/2/10	
SS82	SS535	GCA_900058515.1	2.06115	41.1	171203	2&1/2	Viet Nam	Scaffold	2016/2/10	
BM237a	SS542	GCA_900050015.1	2.09121	41.2	218658	2&1/2	Viet Nam	Contig	2016/2/10	
FX14	SS547	GCA_900062105.1	2.057	41.1	223121	2&1/2	Viet Nam	Contig	2016/2/10	
FX77_1	SS557	GCA_900065275.1	2.04581	41.2	170431	2&1/2	Viet Nam	Contig	2016/2/10	
CTU02SS-DN025	SS585	GCA_900063015.1	2.03813	41.2	131494	2&1/2	Viet Nam	Contig	2016/2/10	
FX336_2	SS588	GCA_900053805.1	2.07749	41.2	239913	2&1/2	Viet Nam	Contig	2016/2/10	
VS329	SS591	GCA_900055505.1	2.057	41.1	169977	2&1/2	Viet Nam	Contig	2016/2/10	
BM191a	SS593	GCA_900060975.1	1.98173	41.2	222121	2&1/2	Viet Nam	Contig	2016/2/10	
VS3804	SS596	GCA_900054345.1	2.06282	41.1	169959	2&1/2	Viet Nam	Contig	2016/2/10	
EN506	SS600	GCA_900056575.1	2.04726	41.2	170437	2&1/2	Viet Nam	Contig	2016/2/10	
BM461	SS603	GCA_900059185.1	2.04863	41.2	199032	2&1/2	Viet Nam	Contig	2016/2/10	
SS33	SS613	GCA_900053635.1	2.05801	41.1	170433	2&1/2	Viet Nam	Contig	2016/2/10	
FX507_1	SS616	GCA_900053235.1	2.04634	41.1	184497	2&1/2	Viet Nam	Contig	2016/2/10	
FX431	SS629	GCA_900056625.1	2.04057	41.1	236341	2&1/2	Viet Nam	Scaffold	2016/2/10	
SS31	SS633	GCA_900053215.1	2.05922	41.1	170450	2&1/2	Viet Nam	Contig	2016/2/10	
BM358a	SS634	GCA_900059495.1	2.04037	41.2	170750	2&1/2	Viet Nam	Contig	2016/2/10	

Strains	ReName	Assembly	Size (Mb)	GC%	N50	Molecular Serotypes	Country	Level	Release Date	Note
EN170	SS635	GCA_900051965.1	2.0603	41.1	234034	2&1/2	Viet Nam	Contig	2016/2/10	
VS2903	SS641	GCA_900054325.1	2.05725	41.1	169698	2&1/2	Viet Nam	Contig	2016/2/10	
EN636	SS642	GCA_900053615.1	2.09543	41.1	170256	2&1/2	Viet Nam	Contig	2016/2/10	
BM436a	SS644	GCA_900063635.1	2.05915	41.1	171311	2&1/2	Viet Nam	Contig	2016/2/10	
VS3756	SS655	GCA_900050895.1	2.06767	41.1	237239	2&1/2	Viet Nam	Contig	2016/2/10	
BM216a	SS656	GCA_900049355.1	1.98593	41.3	222188	2&1/2	Viet Nam	Contig	2016/2/10	
BM209a	SS659	GCA_900060995.1	1.99149	41.2	222214	2&1/2	Viet Nam	Contig	2016/2/10	
CTU02SS-DN026	SS660	GCA_900056615.1	2.03727	41.2	228443	2&1/2	Viet Nam	Contig	2016/2/10	
SS65	SS675	GCA_900058305.1	2.06956	41.3	233889	2&1/2	Viet Nam	Contig	2016/2/10	
FX419	SS680	GCA_900054365.1	2.04191	41.1	222807	2&1/2	Viet Nam	Contig	2016/2/10	
SS30	SS681	GCA_900064685.1	2.0428	41.2	234080	2&1/2	Viet Nam	Contig	2016/2/10	
EN177	SS683	GCA_900065425.1	2.08156	41.1	234084	2&1/2	Viet Nam	Contig	2016/2/10	
SS20	SS689	GCA_900060135.1	2.0534	41.2	234072	2&1/2	Viet Nam	Contig	2016/2/10	
SS85	SS709	GCA_900060145.1	2.08693	41.1	238022	2&1/2	Viet Nam	Contig	2016/2/10	
VS3640	SS716	GCA_900055525.1	2.05779	41.1	169493	2&1/2	Viet Nam	Contig	2016/2/10	
FX527_2	SS721	GCA_900057725.1	2.04644	41.1	221625	2&1/2	Viet Nam	Contig	2016/2/10	
VS3878	SS724	GCA_900055535.1	2.05865	41.1	170459	2&1/2	Viet Nam	Contig	2016/2/10	
BM198a	SS728	GCA_900049995.1	2.05655	41.1	222214	2&1/2	Viet Nam	Contig	2016/2/10	
EN023	SS737	GCA_900061055.1	2.04811	41.2	224858	2&1/2	Viet Nam	Contig	2016/2/10	

Strains	ReName	Assembly	Size (Mb)	GC%	N50	Molecular Serotypes	Country	Level	Release Date	Note
FX275	SS758	GCA_900049685.1	2.0514	41.1	158990	2&1/2	Viet Nam	Contig	2016/2/10	
EN515	SS759	GCA_900056865.1	2.03657	41.2	170718	2&1/2	Viet Nam	Contig	2016/2/10	
VS3699	SS765	GCA_900052005.1	2.04324	41.2	170318	2&1/2	Viet Nam	Contig	2016/2/10	
SS37	SS773	GCA_900057685.1	2.06738	41.1	237753	2&1/2	Viet Nam	Contig	2016/2/10	
SS13	SS777	GCA_900059505.1	2.08629	41.2	198292	2&1/2	Viet Nam	Contig	2016/2/10	
SS16	SS779	GCA_900059515.1	2.04136	41.2	170426	2&1/2	Viet Nam	Scaffold	2016/2/10	
EN031	SS791	GCA_900060335.1	2.0425	41.2	198291	2&1/2	Viet Nam	Contig	2016/2/10	
BM241a	SS799	GCA_900050005.1	2.09283	41.1	223106	2&1/2	Viet Nam	Contig	2016/2/10	
EN330	SS802	GCA_900051975.1	2.04793	41.2	194318	2&1/2	Viet Nam	Contig	2016/2/10	
FX230	SS803	GCA_900065075.1	2.1998	40.9	170720	2&1/2	Viet Nam	Contig	2016/2/10	
BM211a	SS805	GCA_900049115.1	2.03691	41.2	320240	2&1/2	Viet Nam	Contig	2016/2/10	
SS35	SS809	GCA_900063665.1	2.03701	41.2	172157	2&1/2	Viet Nam	Contig	2016/2/10	
EN015	SS810	GCA_900060125.1	2.07934	41.2	223422	2&1/2	Viet Nam	Scaffold	2016/2/10	
EN003	SS816	GCA_900054865.1	2.05896	41.1	189307	2&1/2	Viet Nam	Contig	2016/2/10	
SS21	SS821	GCA_900058765.1	2.07665	41.2	171315	2&1/2	Viet Nam	Contig	2016/2/10	
VS3466	SS824	GCA_900054595.1	2.05799	41.1	169675	2&1/2	Viet Nam	Contig	2016/2/10	
FX428_1	SS838	GCA_900057535.1	2.04046	41.1	214770	2&1/2	Viet Nam	Scaffold	2016/2/10	
SS102	SS851	GCA_900054335.1	2.05095	41.1	223118	2&1/2	Viet Nam	Contig	2016/2/10	
FX40_1	SS852	GCA_900064305.1	2.08259	41.2	170467	2&1/2	Viet Nam	Contig	2016/2/10	

Strains	ReName	Assembly	Size (Mb)	GC%	N50	Molecular Serotypes	Country	Level	Release Date	Note
SS76	SS860	GCA_900056885.1	2.06242	41.1	234114	2&1/2	Viet Nam	Contig	2016/2/10	
FX501	SS872	GCA_900059215.1	2.05837	41.1	170410	2&1/2	Viet Nam	Contig	2016/2/10	
FX415_6	SS875	GCA_900055905.1	2.04083	41.1	200872	2&1/2	Viet Nam	Contig	2016/2/10	
BM303a	SS879	GCA_900049135.1	2.04105	41.2	234075	2&1/2	Viet Nam	Contig	2016/2/10	
BM190a	SS884	GCA_900065055.1	2.0914	41.1	222121	2&1/2	Viet Nam	Contig	2016/2/10	
BM373a	SS893	GCA_900061005.1	2.05983	41.1	170437	2&1/2	Viet Nam	Contig	2016/2/10	
EN253	SS898	GCA_900050865.1	2.08433	41.2	170716	2&1/2	Viet Nam	Contig	2016/2/10	
VS3881	SS903	GCA_900060155.1	2.05805	41.1	169902	2&1/2	Viet Nam	Contig	2016/2/10	
BM203a	SS914	GCA_900060985.1	2.05824	41.1	172754	2&1/2	Viet Nam	Contig	2016/2/10	
VS3861	SS918	GCA_900054355.1	2.05356	41.2	146599	2&1/2	Viet Nam	Contig	2016/2/10	
VS3513	SS938	GCA_900050045.1	2.04653	41.2	170699	2&1/2	Viet Nam	Contig	2016/2/10	
EN050	SS944	GCA_900060345.1	2.07607	41.2	170456	2&1/2	Viet Nam	Contig	2016/2/10	
SS14	SS951	GCA_900053625.1	2.04384	41.2	199279	2&1/2	Viet Nam	Contig	2016/2/10	
SS22	SS963	GCA_900060665.1	2.03903	41.2	223344	2&1/2	Viet Nam	Contig	2016/2/10	
BM411a	SS968	GCA_900061015.1	2.05312	41.1	171844	2&1/2	Viet Nam	Contig	2016/2/10	
VS3121	SS970	GCA_900054875.1	2.05517	41.1	170335	2&1/2	Viet Nam	Contig	2016/2/10	
SS46	SS971	GCA_900057525.1	2.0566	41.1	238294	2&1/2	Viet Nam	Contig	2016/2/10	

Table S3 The performance of 81 binary similarity and distance measures

Methods	AUROC (%)	AUPR (%)	Overall score	No
$S_{JACCARD} = \frac{a}{a+b+c}$	78.81	38.31	109.40	(1)
$S_{DICE} = \frac{2a}{2a+b+c}$	78.81	38.31	109.40	(2)
$S_{CZEKANOWSKI} = \frac{2a}{2a+b+c}$	78.81	38.31	109.40	(3)
$S_{3w-JACCARD} = \frac{3a}{3a+b+c}$	78.81	38.31	109.40	(4)
$S_{NEI\&LI} = \frac{2a}{(a+b)+(a+c)}$	78.81	38.31	109.40	(5)
$S_{SOKAL\&SNEATH-I} = \frac{a}{a+2b+2c}$	78.81	38.31	109.40	(6)
$S_{SOKAL\&MICHENER} = \frac{a+d}{a+b+c+d}$	79.70	39.66	117.40	(7)
$S_{SOKAL\&SNEATH-II} = \frac{2(a+d)}{a+2b+2c}$	79.70	39.66	117.40	(8)
$S_{ROGER\&TANIMOTO} = \frac{2(a+d)}{a+2b+2c}$	79.70	39.66	117.40	(9)
$S_{FAITH} = \frac{a+0.5d}{a+b+c+d}$	68.93	26.85	46.60	(10)
$S_{GOWER\&LEGENDRE} = \frac{a+d}{a+0.5(b+c)+d}$	79.70	39.66	117.40	(11)
$S_{INTERSECTION} = a$	57.23	18.98	12.15	(12)
$S_{INNERPRODUCT} = a+d$	79.70	39.66	117.40	(13)
$S_{RUSSELL\&RAO} = \frac{a}{a+b+c+d}$	57.23	18.98	12.15	(14)
$D_{HAMMING} = b+c$	79.70	39.66	117.40	(15)
$D_{EUCLID} = \sqrt{b+c}$	79.70	39.66	117.40	(16)
$D_{SQUARED-EUCLID} = \sqrt{(b+c)^2}$	79.70	39.66	117.40	(17)
$D_{CANBERRA} = (b+c)^{\frac{2}{2}}$	79.70	39.66	117.40	(18)
$D_{MANHATTAN} = b+c$	79.70	39.66	117.40	(19)
$D_{MEAN-MANHATTAN} = \frac{b+c}{a+b+c+d}$	79.70	39.66	117.40	(20)
$D_{CITYBLOCK} = b+c$	79.70	39.66	117.40	(21)

$D_{MINKOWSKI} = (b+c)^{\frac{1}{1}}$	79.70	39.66	117.40	(22)
$D_{VARI} = \frac{(b+c)}{4(a+b+c+d)}$	79.70	39.66	117.40	(23)
$D_{SIZEDIFFERENCE} = \frac{(\mathrm{b}+\mathrm{c})^2}{(a+b+c+d)^2}$	79.70	39.66	117.40	(24)
$D_{SHAPEDIFFERENCE} = \frac{n(b+c)+(\mathrm{b}-\mathrm{c})^2}{(a+b+c+d)^2}$	78.27	39.62	112.81	(25)
$D_{PATTERNDIFFERENCE} = \frac{4bc}{(a+b+c+d)^2}$	73.50	24.96	51.91	(26)
$D_{LANCE\&WILLIAMS} = \frac{b+c}{(2a+b+c)}$	78.81	38.31	109.40	(27)
$D_{BRAY\&CURTIS} = \frac{b+c}{(2a+b+c)}$	78.81	38.31	109.40	(28)
$D_{HELLINGER} = 2\sqrt{(1-\frac{a}{\sqrt{(a+b)(a+c)}})}$	78.93	38.39	110.07	(29)
$D_{CHORD} = \sqrt{2(1-\frac{a}{\sqrt{(a+b)(a+c)}})}$	78.93	38.39	110.07	(30)
$S_{COSINE} = \frac{a}{\sqrt{(a+b)(a+c)}^2}$	68.99	23.41	37.92	(31)
$S_{GILBERT\&WELLS} = \log a - \log n - \log\left(\frac{a+b}{n}\right) - \log\left(\frac{a+c}{n}\right)$	68.99	23.40	37.88	(32)
$S_{OCHIAI-I} = \frac{a}{\sqrt{(a+b)(a+c)}}$	78.93	38.39	110.07	(33)
$S_{FORBESI} = \frac{na}{(a+b)(a+c)}$	68.99	23.43	37.97	(34)
$S_{Fossum} = \frac{n(a-0.5)^2}{(a+b)(a+c)}$	78.88	38.13	108.95	(35)
$S_{SORGENFREI} = \frac{a^2}{(a+b)(a+c)}$	78.93	38.39	110.07	(36)
$S_{MOUNTFORD} = \frac{a}{0.5(ab+ac)+bc}$	79.76	39.65	117.53	(37)
$S_{OTSUKA} = \frac{a}{((a+b)(a+c))^{0.5}}$	78.93	38.39	110.07	(38)
$S_{MCCONNAUGHEY} = \frac{a^2-bc}{(a+b)(a+c)}$	78.93	38.46	110.34	(39)
$S_{TARWID} = \frac{na-(a+b)(a+c)}{na+(a+b)(a+c)}$	68.99	23.43	37.97	(40)

$S_{KULCZYNSKI-II} = \frac{\frac{a}{2}(2a+b+c)}{(a+b)(a+c)}$	78.93	38.46	110.34	(41)
$S_{DRIVER\&KROEBER} = \frac{a}{2}(\frac{1}{a+b}+\frac{1}{a+c})$	78.93	38.51	110.55	(42)
$S_{JOHNSON} = \frac{a}{a+b}+\frac{a}{a+c}$	78.93	38.46	110.34	(43)
$S_{DENNIS} = \frac{ad-bc}{\sqrt{n(a+b)(a+c)}}$	72.11	29.23	60.39	(44)
$S_{SIMPSON} = \frac{a}{\min(a+b,a+c)}$	73.32	24.42	50.09	(45)
$S_{BRAUN\&BANQUET} = \frac{a}{\max(a+b,a+c)}$	77.82	37.30	102.63	(46)
$S_{FAGER\&McGOWAN} = \frac{a}{\sqrt{(a+b)(a+c)}} - \frac{\max(a+b,a+c)}{2}$	64.18	20.56	23.14	(47)
$S_{FORBES-II} = \frac{na-(a+b)(a+c)}{n\min(a+b,a+c)-(a+b)(a+c)}$	75.05	25.16	56.44	(48)
$S_{SOKAL\&SNEATH-IV} = \frac{\frac{a}{(a+b)}+\frac{a}{(a+c)}+\frac{d}{(b+d)}+\frac{d}{(b+d)}}{4}$	79.88	39.38	116.85	(49)
$S_{GOWER} = \frac{a+d}{\sqrt{(a+b)(a+c)(b+d)(c+d)}}$	60.11	15.15	8.93	(50)
$S_{PEARSON-1} = x^2$ *where* $x^2 = \frac{n(ad-bc)^2}{(a+b)(a+c)(c+d)(b+d)}$	80.00	39.34	117.06	(51)
$S_{PEARSON-II} = (\frac{x^2}{n+x^2})^{1/2}$	80.00	39.34	117.06	(52)
$S_{PEARSON-III} = (\frac{\rho}{n+\rho})^{1/2}$ where $\rho = \frac{ad-bc}{\sqrt{(a+b)(a+c)(b+d)(c+d)}}$	80.37	39.38	118.40	(53)
$S_{PEARSON\&HERON-I} = \frac{ad-bc}{\sqrt{(a+b)(a+c)(b+d)(c+d)}}$	80.41	39.39	118.59	(54)
$S_{PEARSON\&HERON-II} = Cos(\frac{\pi\sqrt{bc}}{\sqrt{ad}+\sqrt{bc}})$	66.28	21.35	28.18	(55)
$S_{SOKAL\&SNEATH-III} = \frac{a+d}{b+d}$	79.70	39.66	117.40	(56)

$S_{SOKAL\&SNEATH-V} = \frac{ad}{(a+b)(a+c)(b+d)(c+d)^{0.5}}$	79.32	36.12	102.88	(57)
$S_{COLE} = \frac{\sqrt{2}(ad-bc)}{\sqrt{(ad-bc)^2-(a+b)(a+c)(b+d)(c+d)}}$	53.12	14.71	3.33	(58)
$S_{STILES} = \log_{10}\frac{n(\lvert ad-bc\rvert-\frac{n}{2})^2}{(a+b)(a+c)(b+d)(c+d)}$	79.97	39.79	118.77	(59)
$S_{OCHIAI-II} = \frac{ad}{\sqrt{(a+b)(a+c)(b+d)(c+d)}}$	80.80	39.39	119.88	(60)
$S_{YULEQ} = \frac{ad-bc}{ad+bc}$	75.24	25.30	57.28	(61)
$D_{YULEQ} = \frac{2bc}{ad+bc}$	75.24	25.30	57.28	(62)
$S_{YULEw} = \frac{\sqrt{ad}-\sqrt{bc}}{\sqrt{ad}+\sqrt{bc}}$	75.24	25.30	57.28	(63)
$S_{KULCZYNSKI-I} = \frac{a}{b+c}$	74.58	36.95	92.28	(64)
$S_{TANIMOTO} = \frac{a}{(a+b)+(a+c)-a}$	78.81	38.31	109.40	(65)
$S_{DISPERSON} = \frac{ad-bc}{(a+b+c+d)^2}$	75.79	33.19	82.34	(66)
$S_{HAMANN} = \frac{(a+d)-(b+c)}{a+b+c+d}$	79.70	39.66	117.40	(67)
$S_{MICHAEL} = \frac{4(ad-bc)}{(a+d)^2+(b+c)^2}$	71.56	27.47	53.98	(68)
$S_{GOODMAN\&KRUSKAL} = \frac{\sigma-\sigma'}{2n-\sigma'} where$ $\sigma = \max(a,b)+\max(c,d)+\max(a,c)+\max(b,d)$, $\sigma' = \max(a+c,b+d)+\max(a+b,c+d)$	79.87	38.76	114.44	(69)
$S_{ANDERBERG} = \frac{\sigma-\sigma'}{2n}$	76.95	32.80	84.28	(70)
$S_{BARONI-URBANI\&BUSER-I} = \frac{\sqrt{ad}+a}{\sqrt{ad}+a+b+c}$	79.95	39.22	116.45	(71)
$S_{BARONI-URBANI\&BUSER-II} = \frac{\sqrt{ad}+a-(b+c)}{\sqrt{ad}+a+b+c}$	79.95	39.22	116.45	(72)
$S_{PEIRCE} = \frac{ab+bc}{ab+2bc+cd}$	50.90	16.95	5.69	(73)
$S_{EYRAUD} = \frac{n^2(na-(a+b)(a+c))}{(a+b)(a+c)(b+d)(c+d)}$	78.65	36.54	102.32	(74)

$S_{TARANTULA}=\frac{\frac{a}{(a+b)}}{\frac{c}{(c+d)}}=\frac{a(c+d)}{c(a+b)}$	59.17	26.12	30.04	(75)
$S_{AMPLE}=\left\|\frac{\frac{a}{(a+b)}}{\frac{c}{(c+d)}}\right\|=\left\|\frac{a(c+d)}{c(a+b)}\right\|$	59.17	26.12	30.04	(76)
$S_{T1}=\frac{\log(1+a+d)}{\log(1+n)}$	79.70	39.66	117.40	(77)
$S_{T2}=\frac{\log(1+n)-\log(1+b+c)}{\log(1+n)}$	79.70	39.66	117.40	(78)
$S_{T3}=\frac{\log(1+a)}{\log(1+n)}$	57.23	18.98	12.15	(79)
$S_{T4}=\frac{\log(1+a)}{\log(1+a+b+c)}$	78.47	37.98	107.11	(80)
$S_{T5}=\frac{\log(1+ad)-\log(1+bc)}{\log(1+n^2/4)}$	78.05	35.15	95.54	(81)

Table S4 *Streptococcus suis* serotype reference strains used in this study

Serotype	Serotype reference strain	Rename	Reference
1	5428	SS755	1
1/2	2651	SS487	1
2	R735	SS910	1
3	4961	SS820	1
4	6407	SS245	1
5	11538	SS770	1
6	2524	SS947	1
7	8074	SS912	1
8	14636	SS602	1
9	22083	SS610	2
10	4417	SS099	2
11	12814	SS880	2
12	8830	SS193	2
13	10581	SS845	2
14	13730	SS101	2
15	NCTC 10446	SS837	2
16	2726	SS949	2
17	93A	SS088	2
18	NT77	SS329	2
19	42A	SS598	2
21	14A	SS392	2
23	89-2479	SS684	3
24	88-5299A	SS278	3
25	89-3576-3	SS108	3
27	89-5259	SS835	3
28	89-590	SS207	3
29	92-1191	SS211	4
30	92-1400	SS881	4
31	92-4172	SS738	4

REFERENCES

1. Perch, B., Pedersen, K.B. and Henrichsen, J. 1983. Serology of capsulated streptococci pathogenic for pigs: six new serotypes of *Streptococcus suis*. J. Clin. Microbiol. 17:993-6.

2. Gottschalk, M., Higgins, R., Jacques, M., Mittal, K.R. and Henrichsen, J. 1986. Description of 14 new capsular types of *Streptococcus suis*. J. Clin. Microbiol. 27:2633-6.

3. Gottschalk, M., Higgins, R., Jacques, M., Beaudoin, M. and Henrichsen, J. 1991. Characterization of six new capsular types (23 through 28) of *Streptococcus suis*. J.

Clin. Microbiol. 29:2590-4.
4. Higgins, R., Gottschalk, M., Boudreau, M., Lebrun, A. and Henrichsen, J. 1995. Description of Six New Capsular Types (29-34) of *Streptococcus Suis.* J. Vet. Diagnostic Investig. 7:405-6.

Table S5 Molecular serotype specific genes of *Streptococcus suis* used in this study

Serotypes	GenBank accession no.	Gene name	Gene length
1&14	JX986790	*cps1I*	1167
2&1/2	KC537364	*cps2I*	1233
3	KC537365	*cps3L*	1038
4	KC537366	*cps4K*	1158
5	KC537367	*cps5L*	1236
6	KC537368	*cps6I*	1194
7	KC537369	*cps7L*	1323
8	JX986797	*cps8K*	1242
9	KC537370	*cps9J*	858
10	JX986799	*cps10M*	1476
11	KC537371	*cps11N*	1140
12	KC537372	*cps12J*	1479
13	JX961643	*cps13L*	1071
15	JX961644	*cps15K*	1107
16	KC537373	*cps16I*	1089
17	KC537374	*cps17O*	1308
18	KC537375	*cps18N*	1329
19	KC537376	*cps19L*	1317
20	KC537377	*cps20I*	1218
21	KC537378	*cps21P*	1092
22	KC537379	*cps22K*	1356
23	JX986802	*cps23J*	1203
24	KC537380	*cps24M*	1281
25	JX986803	*cps25M*	1227
26	KC537381	*cps26P*	1116
27	JX961652	*cps27K*	1137
28	JX961653	*cps28L*	1197
29	JX961654	*cps29L*	1386
30	KC537382	*cps30I*	1356
31	JX961656	*cps31L*	1203
33	KC537383	*cps33K*	1311
Chz	KJ669337.1: 15227-16414	*chzM*	1188
NCL1	KM972281.1: 12825-14072	*cpsM*	1248
NCL2	KM972261.1: 11868-13376	*cpsM*	1509
NCL3	KM972262.1: 9885-11114	*cpsK*	1230
NCL4	KM972264.1: 10732-12030	*cpsL*	1299
NCL5	KM972265.1: 10133-11521	*cpsK*	1389
NCL6	KM972270.1: 10297-11355	*cpsK*	1059

Serotypes	GenBank accession no.	Gene name	Gene length
NCL7	KM972274.1: 14735-15850	*cpsO*	1116
NCL8	KM972289.1: 10185-11390	*cpsM*	1206
NCL9	KT163363.1: 14049-15293	*cpsQ*	1245
NCL10	KT163364.1: 13536-14657	*cpsO*	1122
NCL11	KT163366.1: 11723-12994	*cpsL*	1272
NCL12	KT163368.1: 12364-13188	*cpsM*	825
NCL13	KU665267.1: 13754-15196	*cpsO*	1443
NCL14	KU665270.1: 13018-14280	*cpsN*	1263
NCL15	KU665272.1: 9888-11321	*cpsM*	1434
NCL16	KU665281.1: 9843-11060	*cpsM*	1218
NCL17	KX870054.1: 13090-14382	*cpsM*	1293
NCL18	KX870050.1: 13029-14291	*cpsO*	1263
NCL19	KX870060.1: 15434-16813	*cpsP*	1380
NCL20	KX870075.1: 9661-10752	*cpsK*	1092

Table S6 Strains and plasmids used in by bacterial two-hybrid analyses in this study

Strain or Plasmid	Relevant genotype or description	Source or reference
***S. suis* strain**		
SC19		Reference[1]
***E. coli* strains**		
BTH101	F^-, *cya-99*, *araD139*, *galE15*, *galK16*, *rpsL1* (Str^r), *hsdR2*, *mcrA1*, *mcrB1*	Reference[2]
**Plasmids**		
pUT18	Vector encoding T18 fragment derived from the high copy number vector pUC19; Amp^r	Reference[2]
pKT25	Vector encoding T25 fragment derived from the low copy-number plasmid pSU40; Kan^r	Reference[2]
pUT18-zip	Vector encoding T18 fragment fused in frame with the leucine zipper of GCN4; Amp^r	Reference[3]
pKT25-zip	Vector encoding T25 fragment fused in frame with the leucine zipper of GCN4; Kan^r	Reference[3]
pUT18-gene2-1-1	pUT18 carrying gene2-1-1	This work
pKT25-gene2-1-2	pKT25 carrying gene2-1-2	This work
pUT18-gene2-2-1	pUT18 carrying gene2-2-1	This work
pKT25-gene2-2-2	pKT25 carrying gene2-2-2	This work
pKT25-gene2-3-1	pKT25 carrying gene2-3-1	This work
pUT18-gene2-3-2	pUT18 carrying gene2-3-2	This work
pKT25-gene2-4-1	pKT25 carrying gene2-4-1	This work
pUT18-gene2-4-2	pUT18 carrying gene2-4-2	This work
pKT25-gene2-5-1	pKT25 carrying gene2-5-1	This work
pUT18-gene2-5-2	pUT18 carrying gene2-5-2	This work
pKT25-gene2-6-1	pKT25 carrying gene2-6-1	This work
pUT18-gene2-6-2	pUT18 carrying gene2-6-2	This work
pKT25-gene2-7-1	pKT25 carrying gene2-7-1	This work
pUT18-gene2-7-2	pUT18 carrying gene2-7-2	This work
pKT25-gene2-8-1	pKT25 carrying gene2-8-1	This work

Strain or Plasmid	Relevant genotype or description	Source or reference
pUT18-gene2-8-2	pUT18 carrying gene2-8-2	This work
pKT25-gene2-9-1	pKT25 carrying gene2-9-1	This work
pUT18-gene2-9-2	pUT18 carrying gene2-9-2	This work
pKT25-gene2-10-1	pKT25 carrying gene2-10-1	This work
pUT18-gene2-10-2	pUT18 carrying gene2-10-2	This work
pKT25-gene2-11-1	pKT25 carrying gene2-11-1	This work
pUT18-gene2-11-2	pUT18 carrying gene2-11-2	This work
pUT18-gene2-12-1	pUT18 carrying gene2-12-2	This work
pKT25-gene2-12-2	pKT25 carrying gene2-12-1	This work
pKT25-gene2-13-1	pKT25 carrying gene2-13-1	This work
pUT18-gene2-13-2	pUT18 carrying gene2-13-2	This work
pUT18-gene2-14-1	pUT18 carrying gene2-14-1	This work
pKT25-gene2-14-2	pKT25 carrying gene2-14-2	This work
pUT18-gene3-1-1	pUT18 carrying gene3-1-1	This work
pKT25-gene3-1-2	pKT25 carrying gene3-1-2	This work
pUT18-gene3-1-2	pUT18 carrying gene3-1-2	This work
pKT25-gene3-1-3	pKT25 carrying gene3-1-3	This work
pKT25-gene3-2-1	pKT25 carrying gene3-2-1	This work
pUT18-gene3-2-2	pUT18 carrying gene3-2-2	This work
pUT18-gene3-2-3	pUT18 carrying gene3-2-3	This work
pUT18-gene3-3-1	pUT18 carrying gene3-3-1	This work
pKT25-gene3-3-2	pKT25 carrying gene3-3-2	This work
pUT18-gene3-3-3	pUT18 carrying gene3-3-3	This work
pKT25-gene3-3-3	pKT25 carrying gene3-3-3	This work
pKT25-gene3-4-1	pKT25 carrying gene3-4-1	This work
pUT18-gene3-4-2	pUT18 carrying gene3-4-2	This work
pKT25-gene3-4-3	pKT25 carrying gene3-4-3	This work
pUT18-gene3-4-3	pUT18 carrying gene3-4-3	This work
pKT25-gene3-5-1	pKT25 carrying gene3-5-1	This work
pKT25-gene3-5-2	pKT25 carrying gene3-5-2	This work
pUT18-gene3-5-2	pUT18 carrying gene3-5-2	This work
pUT18-gene3-5-3	pUT18 carrying gene3-5-3	This work
pKT25-gene3-6-1	pKT25 carrying gene3-6-1	This work
pUT18-gene3-6-2	pUT18 carrying gene3-6-2	This work
pKT25-gene3-6-3	pKT25 carrying gene3-6-3	This work
pUT18-gene3-6-3	pUT18 carrying gene3-6-3	This work

Strain or Plasmid	Relevant genotype or description	Source or reference
pKT25-gene3-7-1	pKT25 carrying gene3-7-1	This work
pUT18-gene3-7-2	pUT18 carrying gene3-7-2	This work
pKT25-gene3-7-2	pKT25 carrying gene3-7-2	This work
pUT18-gene3-7-3	pUT18 carrying gene3-7-3	This work
pUT18-gene3-8-1	pUT18 carrying gene3-8-1	This work
pKT25-gene3-8-2	pKT25 carrying gene3-8-2	This work
pUT18-gene3-8-2	pUT18 carrying gene3-8-2	This work
pKT25-gene3-8-3	pKT25 carrying gene3-8-3	This work

References

1. *Bacterial Adenylate Cyclase Two-Hybrid System Kit* manual (Euromedex).
2. Teng L, Dong X, Zhou Y, Li Z, Deng L, Chen H, Wang X, Li J. 2019. Draft genome sequence of hypervirulent and vaccine candidate *Streptococcus suis* strain SC19. Genome Announc. 5:e01484-16.
3. Karimova, G., J. Pidoux, A. Ullmann and D. Ladant. 1998. A bacterial two-hybrid system based on a reconstituted signal transduction pathway. Proc. Natl. Acad. Sci. 95: 5752-6.
